# Genetic Variants Associated With Response to Platinum-Based Chemotherapy in Non-Small Cell Lung Cancer Patients: A Field Synopsis and Meta‐Analysis

**DOI:** 10.3389/bjbs.2024.11835

**Published:** 2024-02-21

**Authors:** Hilary Sito, Mohamad Ayub Khan Sharzehan, Md Asiful Islam, Shing Cheng Tan

**Affiliations:** ^1^ UKM Medical Molecular Biology Institute, Universiti Kebangsaan Malaysia, Kuala Lumpur, Malaysia; ^2^ WHO Collaborating Centre for Global Women’s Health, Institute of Metabolism and Systems Research, College of Medical and Dental Sciences, University of Birmingham, Birmingham, United Kingdom

**Keywords:** platinum-based chemotherapy, non-small cell lung cancer, polymorphism, systematic review, meta-analysis

## Abstract

**Background:** Publications on the associations of genetic variants with the response to platinum-based chemotherapy (PBC) in NSCLC patients have surged over the years, but the results have been inconsistent. Here, a comprehensive meta-analysis was conducted to combine eligible studies for a more accurate assessment of the pharmacogenetics of PBC in NSCLC patients.

**Methods:** Relevant publications were searched in PubMed, Scopus, and Web of Science databases through 15 May 2021. Inclusion criteria for eligible publications include studies that reported genotype and allele frequencies of NSCLC patients treated with PBC, delineated by their treatment response (sensitive vs. resistant). Publications on cell lines or animal models, duplicate reports, and non-primary research were excluded. Epidemiological credibility of cumulative evidence was assessed using the Newcastle-Ottawa Scale (NOS) and Venice criteria. Begg’s and Egger’s tests were used to assess publication bias. Cochran’s Q-test and I^2^ test were used to calculate the odds ratio and heterogeneity value to proceed with the random effects or fixed-effects method. Venice criteria were used to assess the strength of evidence, replication methods and protection against bias in the studies.

**Results:** A total of 121 publications comprising 29,478 subjects were included in this study, and meta-analyses were performed on 184 genetic variants. Twelve genetic variants from 10 candidate genes showed significant associations with PBC response in NSCLC patients with strong or moderate cumulative epidemiological evidence (increased risk: *ERCC1* rs3212986, *ERCC2* rs1799793, *ERCC2* rs1052555, and *CYP1A1* rs1048943; decreased risk: *GSTM1* rs36631, *XRCC1* rs1799782 and rs25487, *XRCC3* rs861539, *XPC* rs77907221, *ABCC2* rs717620, *ABCG2* rs2231142, and *CDA* rs1048977). Bioinformatics analysis predicted possible damaging or deleterious effects for *XRCC1* rs1799782 and possible low or medium functional impact for *CYP1A1* rs1048943.

**Conclusion:** Our results provide an up-to-date summary of the association between genetic variants and response to PBC in NSCLC patients.

## Introduction

Lung cancer is the leading cause of cancer-related deaths worldwide, accounting for 18.0% of all cancer deaths in 2020 [1]. Non-small cell lung cancer (NSCLC), which constitutes 85% of all lung cancer cases, is the major subtype of the cancer [2]. Platinum-based chemotherapy (PBC) is commonly used as first-line treatment and also as adjuvants to radiotherapy or surgery in patients with late-stage NSCLC [3–5]. However, only 30%–40% of patients show a good response to PBC, and tests for extreme drug resistance have shown high variability in chemoresistance (24%–88%) [6, 7].

The high variability in patient response to PBC has been attributed to individual genetic variants. In recent years, there has been a surge of publications on the pharmacogenetics of PBC in NSCLC patients. Many studies have reported that the genetic variants associated with platinum chemoresistance are involved in the DNA repair pathway, cellular trafficking and drug transport, and metabolic pathways [8–10]. However, the results of published studies are mostly inconsistent and inconclusive. The main reasons for the inconsistency of results are small sample sizes leading to low statistical power, heterogeneity of ethnicities in the different studies due to population stratification, or differences between histological subtypes [11]. A comprehensive meta-analysis is therefore necessary to yield a more precise estimate of the association between genetic variants and PBC responses and also to provide a field synopsis of research in this area. To our knowledge, the most recent comprehensive meta-analysis reporting the association of genetic variants with response to PBC was published in 2017, and only included studies up to 31 January 2016 [12]. As there have been numerous new studies published after January 2016, the inclusion of these studies may yield different results than the previous meta-analysis [13].

Here, we aim to identify, strengthen and interpret the associations of genetic variants with response to PBC in patients with NSCLC patients using a comprehensive systematic review and meta-analysis. We used the standardized guidelines including the Venice criteria to systematically assess the credibility of all relevant studies from publications [14–17]. In this work, a total of 184 genetic variants were examined based on the reported genotype frequencies for each variant corresponding to either chemoresistance or good response to PBC in NSCLC patients [18]. Results were also stratified by ethnicity to provide greater insight into the underlying factors that influence response to PBC.

## Materials and Methods

### Search Strategy, Eligibility Criteria and Data Extraction

The meta-analysis was reported in accordance with the PRISMA guidelines [16]. A comprehensive literature search was performed on PubMed, Scopus, and Web of Science (WoS) databases using combinations of three groups of keywords: platinum OR cisplatin OR carboplatin OR oxaliplatin OR nedaplatin; polymorphism OR SNP OR variant OR mutation; NSCLC OR non-small cell lung cancer up to 15 May 2021. We then used alternative wording for the above terms for a supplemental search. No language restrictions were applied to the literature search, and languages other than English, Malay and Chinese were translated using a professional translation service. Authors were contacted by email to obtain the missing full text publications. Two investigators (HS and SCT) independently selected eligible studies based on the predefined inclusion and exclusion criteria. Disagreements between the two investigators were resolved through a process of discussion and mutual consensus. The inclusion criteria were: 1) included patients should be confirmed as having NSCLC, 2) PBC was administered for treatment, 3) contained data on genotype and allele frequencies (or sufficient data to derive this information) and on the treatment response. The following studies were excluded: 1) they were performed in cell lines or animals, 2) duplicate reports, 3) non-original research (e.g., reviews, case reports, and meta-analyses). If it was ambiguous whether two or more studies contained overlapping data, we contacted the study author(s) by email to verify. The study protocol was prospectively registered at PROSPERO (registration number: CRD42021254570).

Relevant data were extracted independently by two investigators (HS and SCT), and discrepancies were resolved by discussion and mutual consensus. The following information was extracted from each eligible study: first author, publication year, ethnicity, genotyping methods, gene and variant information, chemotherapeutic agents, SNPs, and disease stage. The ethnicity of study participants was broadly divided into European and Asian. The quality of eligible studies was independently assessed by two investigators (HS and SCT) using the Modified Newcastle-Ottawa Scale for Case-Control Studies of Genetic Association [19], and discrepancies between the investigators were resolved by discussion and mutual consensus. A study was considered to be of good quality if it had 6 or more stars [14].

### Data Management and Abstraction

Two studies by Mlak et al. [20, 21] had overlapping datasets, so only the more recent dataset with the larger population data was used, as recommended by Little et al. [22]. To ensure that the nomenclature of genetic variants was consistent, the “rs” number identifiers from the public single nucleotide polymorphism (SNP) database (dbSNP) [23] were used to designate shortlisted genetic variants. For the remaining variants without “rs” numbers, the nomenclature described in the original articles was used.

### Statistical Analysis

We used genotype frequencies of NSCLC patients classified into the non-responding and responding groups as indicators of PBC response. In the included studies which adhered to RECIST criteria, patients were divided as follows: the non-responding group comprised patients with stable or progressive diseases (SD and PD), while the responding group comprised complete and partial responders (CR and PR) [18]. Meta-analyses were performed under five genetic models: 1) homozygous model (homozygous variant genotype versus wild type genotype), 2) heterozygous model (heterozygous genotype versus wild type genotype), 3) dominant model (heterozygous and homozygous variant genotypes versus wild type genotype), 4) recessive model (homozygous variant versus wild type and heterozygous genotypes), and 5) allele model (variant allele versus wild type allele). If a study reported the frequency of the homozygous and heterozygous genotypes but not that of the allele, we derived the allele model frequency by calculating the sum of the frequencies of the individual genotypes. In contrast, if a study reported the allele frequency but did not distinguish between homozygous and heterozygous genotypes, the data analysis was performed according to the allele model but not other models. This led to variations in the number of studies included in our meta-analysis for the different genetic models. This methodology is consistent with standard practices in the field of meta-analysis of genetic association studies [24]. Conventional comparisons from publications were used to evaluate the effects of genetic variants that were not single nucleotide polymorphisms (e.g., *GSTM1* [null vs. present]).

Heterogeneity among studies was assessed by using Cochran’s Q test and the I^2^ test. Studies with a Q test *p*-value of <0.10 and and I^2^ heterogeneity value of >50% were considered highly heterogeneous. The random-effects method was used to calculate the pooled odds ratio (OR) and the corresponding 95% confidence interval (CI) in studies with high heterogeneity to estimate the association between genetic variants and response to platinum-based therapy; otherwise, a fixed-effects method was used. Statistical assessment of publication bias was performed using Begg’s rank correlation and Egger’s linear regression tests, followed by visual inspection of the funnel plot for asymmetry. The significance level was set at 0.05 unless otherwise stated. The strength of epidemiological evidence was assessed using the Venice criteria [15]. Subgroup analysis was performed based on the ethnicity of the patients and methodological quality of the studies. Sensitivity analyses were performed by iteratively omitting one study at a time to determine the stability and robustness of the results. All statistical analyses were performed using STATA/S.E 14.0 (StataCorp, College Station, TX).

### Functional Annotations

We performed further genomic annotations for genetic variants that showed significant associations with PBC response with moderate or strong epidemiological evidence. We used Ensembl Variation Pathogenicity Predictions [25] which includes a wide range of algorithms such as SIFT and PolyPhen-2 for variations leading to amino acid substitutions, CADD to measure variant deleteriousness, and REVEL, MutationAssessor and MetaLR for human missense variant pathogenicity scores.

## Results

### Study Selection and Characteristics

**FIGURE 1:**
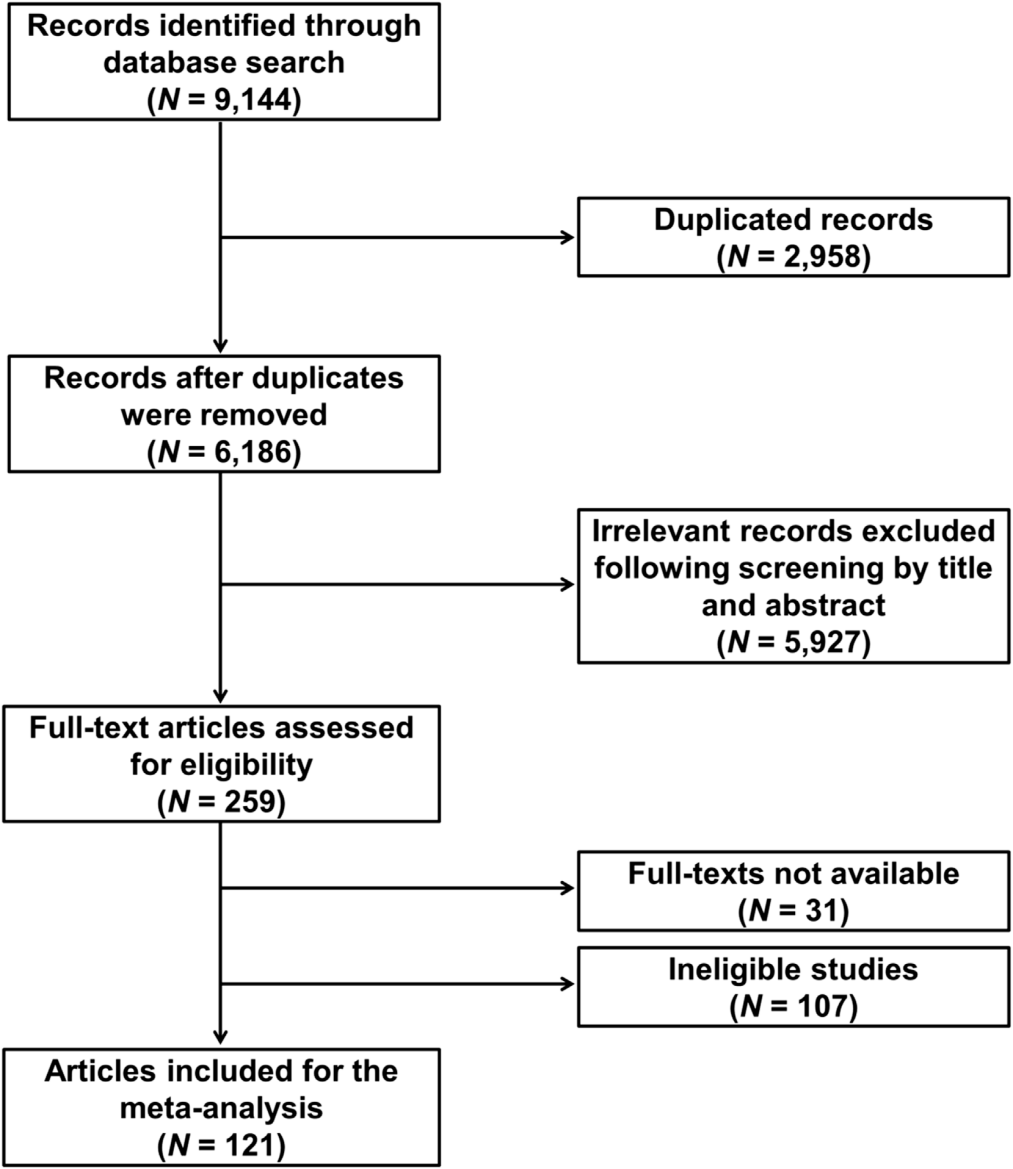
Flowchart of literature search and selection.

The flowchart of the study selection is shown in Figure 1. The initial comprehensive literature search for relevant publications in the PubMed, Scopus, and Web of Science databases yielded 9,144 results, which were subjected to deduplication and screening to exclude publications that did not contain genotype information or were irrelevant. The selection process identified 121 eligible publications (Supplementary Table S1). The eligible studies involved 29,478 NSCLC patients and reported a total of 184 genetic variants from 95 genes. The vast majority (∼85%) of the studies were conducted in Asians. More than 80% of the publications focused only on advanced NSCLC (stages III–IV), while the remainder included early stages too.

### Overall Meta-Analysis Findings

Among the 184 genetic variants analyzed, a total of 67 genetic variants in 49 candidate genes showed statistically significant associations with response to platinum chemotherapy in at least one genetic model (*p* < 0.05) (Supplementary Table S2). With the exception of *BAX* rs4645878 (G/A) and *XRCC1* rs25487 (G/A), all other genetic variants were consistent in the magnitude of association across different genetic models (either OR>1 or OR<1). Of the remaining 66 genetic variants, 32 were putative “risk” variants that are significantly associated with increased risk of chemoresistance, while 33 were “protective” variants associated with increased sensitivity to PBC.

Credibility assessment using the Venice criteria revealed that the association of one genetic variant (*ERCC2* rs1799793) had strong cumulative epidemiological evidence, whereas 12 variants (*ABCC2* rs717620, *ABCG2* rs2231142, *CDA* rs1048977, *CYP1A1* rs1048943, *ERCC1* rs3212986, *ERCC2* rs1799793 and rs1052555, *GSTM1* rs36631, *XPC* rs77907221, *XRCC1* rs1799782 and rs25487, and *XRCC3* rs861539) showed moderate evidence in at least one genetic model (it should be noted that the cumulative epidemiological evidence of *ERCC2* rs1799793 was strong under the dominant genetic model and moderate under the allele model) (Supplementary Table S2; Supplementary Figures S1–S12). Under the dominant model, *ERCC2* rs1799793 was associated with an increased risk of PBC chemoresistance (OR = 1.186, 95% CI = 1.000–1.407), although the association was at borderline significance (*p* = 0.049). Similarly, an increased risk of chemoresistance was observed for under the allele model (OR = 1.311, 95% CI = 1.082–1.590, *p* < 0.01), as well as for *CYP1A1* rs1048943 (dominant model, OR = 2.593, 95% CI = 1.535–4.381, *p* < 0.01; heterozygous model, OR = 2.512, 95% CI = 1.437–4.392, *p* < 0.01; allele model, OR = 1.851, 95% CI = 1.303–2.631, *p* < 0.01), *ERCC1* rs3212986 (recessive model, OR = 1.685, 95% CI = 1.167–2.433, *p* < 0.01; homozygous model, OR = 1.980, 95% CI = 1.346–2.913, *p* < 0.01; allele model, OR = 1.417, 95% CI = 1.052–1.910; *p* = 0.02), *ERCC2* rs1052555 (dominant model, OR = 1.473, 95% CI = 1.063–2.042, *p* = 0.02), and *XRCC1* rs25487 (recessive model, OR = 1.526, 95% CI = 1.105–2.107, *p* = 0.01). In contrast, significantly decreased risk for chemoresistance was observed for *ABCC2* rs717620 (allele model, OR = 0.044, 95% CI = 0.008–0.241, *p* < 0.01), *ABCG2* rs2231142 (recessive model, OR = 0.480, 95% CI = 0.316–0.727, *p* < 0.01; homozygous model, OR = 0.477, 95% CI = 0.306–0.741, *p* < 0.01; allele model, OR = 0.754, 95% CI = 0.612–0.929, *p* < 0.01), *CDA* rs1048977 (allele model, OR = 0.516, 95% CI = 0.342–0.778, *p* = 0.02), *GSTM1* rs36631 (allele model, OR = 0.531, 95% CI = 0.411–0.687, *p* < 0.01), *XPC* rs77907221 (heterozygous model, OR = 0.369, 95% CI = 0.193–0.703, *p* < 0.01; allele model, OR = 0.639, 95% CI = 0.465–0.877, *p* < 0.01), *XRCC1* rs1799782 (recessive model, OR = 0.611, 95% CI = 0.453–0.825, *p* < 0.01; homozygous 0.494, 95% CI = 0.316–0.773, *p* < 0.01; allele model, OR = 0.625, 95% CI = 0.473–0.825, *p* < 0.01), *XRCC1* rs25487 (homozygous model, OR = 0.647, 95% CI = 0.455–0.920, *p* = 0.02), and *XRCC3* rs861539 (allele model, OR = 0.734, 95% CI = 0.586–0.918, *p* < 0.01; dominant model, OR = 0.686, 95% CI = 0.508–0.925, *p* = 0.02).

### Sensitivity Analysis

Sensitivity analysis of the genetic variants associated with response to PBC that had moderate or high epidemiological evidence showed that the results were not significantly altered when any of the individual studies was omitted from the analysis, suggesting that the associations were robust and not driven by any single study. The sensitivity analysis plots for these variants are provided in Supplementary Figures.

### Subgroup Analysis

Subgroup analysis sorted by ethnicity was performed for *ERCC1* rs11615, *ERCC1* rs3212986 and *ERCC2* rs13181 as only these variants were reported in at least three studies in the Asian and European populations (Supplementary Table S3; Supplementary Figures S13–S15). For *ERCC1* rs11615, an increased risk of PBC chemoresistance was observed in Asians under all genetic models examined (homozygous model, OR = 1.287, 95% CI = 0.729–2.272; heterozygous model, OR = 1.165, 95% CI = 0.854–1.589; dominant model, OR = 1.189, 95% CI = 0.843–1.676; recessive model, OR = 1.287, 95% CI = 0.729–2.272; allele model, OR = 1.112, 95% CI = 0.835–1.481), whereas a decreased risk was observed in Europeans (homozygous model, OR = 0.851, 95% CI = 0.510–1.418; heterozygous model, OR = 0.771, 95% CI = 0.496–1.197; dominant model, OR = 0.789, 95% CI = 0.520–1.197; recessive model, OR = 0.964, 95% CI = 0.659–1.410; allele model, OR = 0.923, 95% CI = 0.729–1.169), although the associations were not statistically significant (*p* > 0.05).

Similarly, *ERCC1* rs3212986 was statistically significantly associated with an increased risk of PBC chemoresistance in Asians (dominant model, OR = 1.556, 95% CI = 1.078–2.244, *p* = 0.018), but a decreased risk was observed in Europeans with borderline lack of significance (OR = 0.718, 95% CI = 0.510–1.010, *p* = 0.057).

*ERCC2* rs13181 also showed a similar result, with OR was <1 for Europeans in the allele, dominant and heterozygous models, but OR > 1 for Asians in the allele and dominant models. Nevertheless, these associations were not statistically significant (*p* > 0.05; Supplementary Table S3).

Subgroup analysis by methodological quality of the studies was not performed because the vast majority of the included studies were of good quality.

### Publication Bias

As shown in Supplementary Table S2, publication bias was observed in several genetic variants (*p* < 0.05 in both Begg’s and Egger’s tests). However, the presence of publication bias would result in the genetic variants being classified as having weak epidemiological evidence according to the Venice criteria. Therefore, none of the shortlisted variants (those significantly associated with PBC response in NSCLC patients with strong or moderate cumulative epidemiological evidence, i.e., *ABCC2* rs717620, *ABCG2* rs2231142, *CDA* rs1048977, *CYP1A1* rs1048943, *ERCC1* rs3212986, *ERCC2* rs1799793 and rs1052555, *GSTM1* rs36631, *XPC* rs77907221, *XRCC1* rs1799782 and rs25487, and *XRCC3* rs861539) showed the presence of publication bias. Nevertheless, it should be noted that the results of *CDA* rs1048977 (allele model), *CYP1A1* rs1048943 (heterozygous, dominant, and allele models), *ERCC2* rs1052555 (dominant model), *GSTM1* rs36631, *XPC* rs77907221 (heterozygous and allele models) and *XRCC1* rs25487 (recessive model) should be interpreted with caution as the publication bias of these genetic variants was either at the borderline of statistical significance in Begg’s or Egger’s test, or could not be analyzed with Egger’s test because only two studies were available for the meta-analysis. Despite this, obvious asymmetry was not observed for any of these variants (Supplementary Figures S16–S27).

### Functional Annotation

Bioinformatics analysis was performed for the shortlisted genetic variants to clarify the functional effects of the variants. The missense variant *XRCC1* rs1799782 could have a deleterious effect or probably damaging effect because it has low SIFT scores of 0.01–0.04 and high PolyPhen-2 scores of 0.393–0.999 for most of the *XRCC1* transcripts. Another missense variant *CYP1A1* rs1048943 has a medium Mutation Assessor score of 0.587 for the prediction of the functional impact of amino acid substitutions. The remaining missense variants for *ERCC2* rs1799793, *XRCC1* rs25487, *XRCC1* rs861539 and *ABCG2* rs2231142 showed scores for benign and tolerant variants. Pathogenicity prediction algorithms were not available for *ERCC1* rs11615, *ERCC1* rs3212986, *XPC* rs77907221, *ERCC2* rs1052555, *CDA* rs1048977 and *ABCC* rs717620 as the transcripts were either synonymous variants, downstream gene variants, 5′-UTR or 3′-UTR variants, nonsense-mediated decay variants, non-coding exon variants, or intron variants.

### Non-Significant Associations

A non-significant association (*p* > 0.05) in at least one genetic model examined was found for 171 genetic variants in 95 genes, many of which overlapped with those showing significant associations. The list of the non-significant associations is shown in Supplementary Table S4.

## Discussion

To our knowledge, the most recent comprehensive meta-analysis examining associations between genetic variants and response to PBC in NSCLC patients was published in 2017, with the literature search last conducted on 31 January 2016 [12]. Since then, a large number of studies reporting the associations of genetic variants to response to PBC have been published. Here, we performed an updated meta-analysis that identified 12 genetic variants in 10 candidate genes that were statistically significantly associated with response to PBC in NSCLC patients with strong or moderate cumulative epidemiological evidence.

A large proportion of the genetic variants are involved in DNA synthesis and repair, consistent with the previous meta-analysis [12]. The mechanism of platinum drugs involves the formation of DNA lesions that lead to apoptosis of cancer cells. Therefore, genetic variants affecting DNA synthesis and repair would influence efficacy of PBC [26, 27]. *ERCC1* is one of the genes involved in nucleotide excision repair (NER). High expression of ERCC1 was found in cisplatin-resistant cancer cells, and can be used to predict low clinical efficacy of cisplatin [28]. However, we demonstrated that the *ERCC1* rs11615 was not significantly associated with PBC response under all genetic models. This is in contrast to with previous meta-analyses and gene association studies which showed significant associations with response to PBC [12, 20, 29–38]. The results may be due to a bias in favor skewed results towards of selected ethnicities, as new studies have been added since the previous meta-analysis. This postulation is supported by the results of our subgroup analysis, which showed that the Asian population has a higher OR compared to the European population. The *ERCC1* rs11615 variant allele has been suggested by several studies to affect NER by reducing *ERCC1* transcripts, which impairs DNA repair and leads to DNA damage accumulation [39, 40], but the functional studies revealed similar results for both the variant and wild type alleles in other studies [41]. The inconsistencies in the functional studies could be due to indirect mechanisms such as linkage disequilibrium or regulation by other factors. The variant allele of *ERCC1* rs3212986 was also found to be associated with PBC response in the current meta-analysis, which is consistent with previous studies [12, 30, 34, 42–46]. Several studies have suggested the *ERCC1* rs3212986 variant may modulate mRNA expression, because it is located in the 3′-UTR, is in linkage disequilibrium with the neighboring *XRCC1* and *XPD* genes, which may affect mRNA stability, and the bioinformatics analysis of *ERCC1* rs32123986 revealed the possible alteration of secondary structure in the 3′-UTR and post-transcriptional regulation via binding miRNAs [40, 47, 48]. The *ERCC1* rs3212986 showed population stratification in the subgroup analysis, with the Asian population having a higher OR (1.556, 95% CI = 1.078–2.244) compared to the European population under the dominant genetic model (OR = 0.718, 95% CI = 0.510–1.010). The underlying mechanisms for the racial differences in PBC response are not clearly defined, but could be due to gene-gene or gene-environment interactions, which include differences in ethnicity or lifestyle [49–51].

*ERCC2 (XPD)* is another gene involved in NER, with XPD serving the DNA helicase subunit in TFIIH [52]. Genetic variants of *ERCC2* have been shown to be associated with impaired DNA repair capacity, leading to accumulation of DNA adducts [53]. The variant allele of *ERCC2* rs1052555 was also identified in this meta-analysis to be associated with response to PBC, consistent with Li et al. and Tan et al. [12, 54]. The *ERCC2* rs1052555 is a silent substitution predicted to form a splicing abolish domain or exon splicing enhancer that affects post-transcriptional mRNA splicing [54]. The altered splicing could result in different expression levels of ERCC2 that contribute to PBC response. On the other hand, the variant allele of *XRCC1* rs1799782 was found to be associated with a protective effect against PBC chemoresistance, which was in agreement with several studies, including a recent meta-analysis by Zhang et al. [12, 55–65]. One possible mechanism by which the variant allele of *XRCC1* rs1799782 protects against PBC chemoresistance is by interfering with the repair of DNA breaks caused by platinum drugs, as the *XRCC1* rs1799782 was predicted to be deleterious or damaging by bioinformatics tools. However, other functional studies suggest that allelic substitution of the variant may indirectly affect the stability of *XRCC1* via interactions with miRNA and polymerases involved in DNA repair [66, 67]. Similarly, the variant allele of *XRCC3* rs861539 was also demonstrated to be associated with a protective effect against chemoresistance, consistent with the meta-analysis by Tan et al. [12]. The protective effect of *XRCC3* rs861539 against chemoresistance is supported by the bioinformatics prediction of a resulting benign and tolerated XRCC3 that contributes to the proper function of DNA repair.

Interestingly, after incorporating data from recent publications [21, 45, 68], we identified several new significant associations that were not found to be statistically significant in the previous meta-analysis by Tan et al. These include variants in genes involved in DNA repair (*ERCC2* rs1799793, *XRCC1* rs25487, and *XPC* rs77907221). The variant allele of *ERCC2* rs1799793 showed associations with PBC chemoresistance, supporting the findings of a previous meta-analysis by Qiu et al. [69]*.* It is unlikely that *ERCC2* rs1799793 causes PBC chemoresistance by affecting DNA repair, as our bioinformatics analysis predicted that the genetic variant is benign and tolerated. Instead, the *ERCC2* rs1799793 may indirectly contribute to platinum chemoresistance by dysregulating apoptosis, as the resulting Asp-to-Asn substitution led to a 2.5-fold increased apoptotic response in a lymphoblastoid cell line [70]. Similarly, the variant allele of *XRCC1* rs25487 also showed associations with PBC chemoresistance, consistent with a recent meta-analysis by Zhang et al. [61]. Bioinformatics analyses predicted that the *XRCC1* rs25487 variants are benign and tolerated, suggesting that the genetic variant also does not directly affect DNA repair to result in PBC chemoresistance. On the other hand, *XPC* rs77907221 insertions were found to be associated with better PBC response here consistent with a few older studies [71, 72]. Since there are only a few studies on the functional effects of *XPC* SNPs*,* the causal mechanism of *XPC* rs77907221 is unclear. However, the strong linkage disequilibrium between *XPC* rs7790722, *XPC* rs2228001 and *XPC* intron 11 may make a synergistic contribution to the PBC response [73, 74].

Besides, whereas Tan et al. previously reported significant associations of *XPA* rs1800975, *ERCC2* rs13181 and *ERCC5* rs2296147 with PBC response, this result was not replicated in our work. For *XPA* rs1800975 and *ERCC5* rs2296147, we could find only two eligible studies and therefore, classified the variants as having poor cumulative epidemiologic evidence. For *ERCC2* rs13181, no statistically significantly association with PBC response was found in our meta-analysis, possibly due to the high heterogeneity score in the homozygous, recessive, dominant and allele models after the addition of recent publications. Subgroup analysis showed that Asians had a higher OR than Europeans, suggesting that population stratification contributes to the high heterogeneity score. The confounding effect of ethnicity is evident because *ERCC2* rs13181 showed significant associations in Europeans but not in Asians in the dominant model.

Metabolic and detoxification regulators also account for a large proportion of the genetic variants identified as having significant associations with PBC responses in this meta-analysis. The cytidine deaminase (CDA) enzyme plays a critical role in the metabolism and inactivation of gemcitabine, which is commonly used in combination with platinum drugs to treat NSCLC [75, 76]. *CDA* rs1048977 is a newly identified genetic variant that shows a significant association with PBC response, which was not reported in the previous meta-analysis by Tan et al. [12] The variant allele of *CDA* rs1048977 showed a significant association with better response to PBC in the allele and recessive models, which is consistent with Hu et al. and Ludovini et al., although in the recessive model it was considered as having weak cumulative epidemiologic evidence [77, 78]. Ludovini et al. suggested that the *CDA* rs1048977 is associated with better response due to lower enzyme activity which results in high drug availability. However, a more recent study by Ciccolini et al. showed that the polymorphism did not alter CDA activity, suggesting indirect regulatory mechanisms [78, 79].

*CYP1A1* is another gene involved in the metabolism of antineoplastic drugs and has been shown to influence responses to PBC [80]. *CYP1A1* rs1048943 is another newly identified genetic variant in this current meta-analysis that shows a significant association with response to PBC. The variant allele of *CYP1A1* rs1048943 was also newly identified in this meta-analysis to be significantly associated with PBC chemoresistance and classified as having moderate cumulative epidemiological evidence in the heterozygous, dominant, and allele models. Interestingly, *CYP1A1* rs1048943 had a slight tendency to deleterious functional effects based on the Mutation Assessor bioinformatics score, which assesses the evolutionary conservation of affected amino acids in protein homologs. The *CYP1A1* rs1048943 is another newly identified genetic variant that has not been reported to show associations with PBC response in any previous meta-analysis, but was previously shown to be significantly associated with lung cancer susceptibility [81]*.* Furthermore, the *CYP1A1* rs1048943 variant allele has previously been shown to have higher enzyme activity that promotes DNA adduct accumulation [82–84].

*ABCC2* and *ABCG2* are drug transporters that play critical roles for the influx and efflux of platinum drugs [85]. Overexpression of ABCC2 during cisplatin treatment has been shown to contribute to cisplatin chemoresistance due to less DNA-cisplatin adduct formation, and lower intracellular accumulation of cisplatin [85–90]. In this meta-analysis, we identified two significant variants in these genes that had not been reported to be associated with response to PBC in previous meta-analyses, i.e., *ABCC2* rs717620 and *ABCG2* rs2231142. The drug transporters *ABCC2* rs717620 variant allele and *ABCG2* rs2231142 variant allele were both found to be significantly associated with protective effects against PBC chemoresistance. The better response of NSCLC patients to the *ABCC2* rs717620 variant allele is consistent with the reports by Han et al. and Qiao et al [91, 92]*.* The *ABCC2* rs717620 may have indirect effects on PBC response, as Zhang et al. reported no effects on mRNA expression or downstream open reading frame translation, whereas Nguyen et al. reported that the *ABCC2* rs717620, together with *ABCC2* rs18885301 and *ABCC2* rs2804402, increased promoter activity by 35% [93, 94]. Similarly, the variant allele of *ABCG2* rs2231142 was also reported to be associated with better PBC response by Qiao et al. [95]. The improved PBC response by *ABCG2* rs2231142 may be due to reduced efflux of platinum drugs from tumor cells using ABCG2. Since *ABCG2* rs2231142 was predicted to be benign or tolerated in our bioinformatics analysis, the decreased ABCG2 activity may occur indirectly via regulation of protein levels. Previous studies have suggested that *ABCG2* rs2231142 elicits its good PBC response by having lower protein levels in the lungs and increased platinum bioavailability in cell lines [96–101]. Previous meta-analyses by Tan et al. and Wei et al. also identified *MTHFR* rs18001133 and *MDR* rs1045642 as genetic variants that showed significant associations with PBC response [12, 33], but no statistically significant association was observed in this current meta-analysis. The lack of significant associations may be due to the significant heterogeneity reported for most genetic models after the addition of new publications.

The current meta-analysis provides the most up-to-date field synopsis and assessment of the associations between genetic variations and response to PBC. In addition, there are sufficient data from recent publications to allow the meta-analyses to be performed for all genetic models (whereas the previous meta-analysis by Tan et al. was limited to the homozygous, heterozygous, and dominant models). The allele model here combined both the homozygous and heterozygous into a single allele model. One advantage provided by the allele model is to distinguish the biological effects when heterozygous and homozygous genotypes are similar. Furthermore, by focusing on allele frequencies rather than genotypes in our meta-analysis, we take advantage of the increased number of observations, essentially doubling our sample size since each individual contributes two alleles. This approach not only increases the statistical power of our study and allows for more precise detection of genetic associations, but also shifts our focus more directly to the individual effects of the alleles, allowing for a clearer understanding of their role in the disease under study. Our study findings provide the impetus for clinical applications in precision medicine, where complementary data on gene-gene, gene-environment, functional studies, and validation in local population of patients will drive the development of potential prognostic biomarkers in the clinical setting. Identification of variants that showed a robust association with response to PBC could contribute to a more accurate assessment of the potential target population for optimizing the efficacy of PBC [102]. These findings could be helpful in the development of genetic tests that can predict the response of patients to PBC and thus enable a more targeted selection of therapy [103]. For example, patients with genotypes associated with improved respond to PBC could be prioritized for the treatment, which would improve treatment efficacy and patient outcomes. In addition, understanding the genetic basis of PBC response may lead to optimization of drug dosing. Personalized dosing based on individual genetic profiles may maximize therapeutic efficacy while minimizing adverse effects, which could significantly improve the quality of life of NSCLC patients undergoing treatment. The identified genetic variants also provide insights into the molecular mechanisms underlying PBC resistance [104]. This knowledge is invaluable for drug development as it can guide the development of new agents that can overcome resistance or improve the efficacy of existing treatments. This genetic knowledge could also facilitate the development of combination therapies. By understanding the genetic profiles that drive resistance to PBC, clinicians can better select complementary treatments that may overcome resistance and improve overall treatment efficacy. In addition, these findings also have implications for health economics [105]. By identifying patients who are more likely to benefit from PBC, healthcare resources can be allocated more efficiently, reducing the cost of ineffective treatments and the management of side effects. The discovery of these genetic variants also opens up opportunities for further research. For example, investigating the interaction between these genetic factors and environmental or lifestyle factors could lead to a more comprehensive understanding of the response to PBC in NSCLC, ultimately leading to improved patient care and better outcomes.

However, there are several limitations to this meta-analysis. First, more than 80% of the genetic polymorphisms identified in the eligible publications were reported in only one or two studies, resulting in small sample sizes and led to having poor cumulative epidemiologic evidence for a number of genetic variations. The limited number of studies on individual variants also prevented us from performing sensitivity analyses, subgroup analyses or tests for publication bias, which may be a source of heterogeneity. We also did not examine the gene-gene or gene-environment interactions that might influence the efficacy of PBC. It is also possible that individual genetic variants identified to be associated with response to PBC here are not the causal variants. The genetic variants could be in linkage disequilibrium with other SNPs or alter the stability of the resulting mRNA. The gene-gene and gene-environment interactions, along with the possible linkage disequilibrium with causal variants, need to be further investigated before the significant variants are used as clinical prognostic biomarkers. For instance, the combined effects of *ERCC1* rs11615, *ERCC1* rs3212986 and *ERCC2* rs1799793 were shown to decrease the overall survival in non-squamous NSCLC patients undergoing pemetrexed/platinum-based chemotherapy but the individual effects of SNPS towards OS were not significant [106]. The OS of the patient decreased with the presence of every unfavorable allele from approximately 30 months with two to three unfavorable alleles to 11.8 months (*p* = 0.01) with four unfavourable alleles [106]. In addition, genome-wide scans should also be conducted in more populations to identify novel genetic variants associated with response to PBC in an unbiased manner. It would also be interesting to investigate whether these genetic variants are associated with lncRNA dysregulations and mitochondrial DNA alterations, which have recently been shown to influence cisplatin response [107, 108]. Finally, functional predictions of significant genetic variants were only made using *in silico* methods, which use different algorithms that assess the degree of conservation of amino acids across different species, physiochemical properties, or the combination of functional data and variant annotations, leading to discrepancies in the predictions [109]. Thus, the results of functional predictions should be interpreted with caution. Despite possible discrepancies in functional prediction, the use of multiple algorithms is advantageous as it allows for a comprehensive analysis, leading to more robust and reliable conclusions [110]. This approach allows for a balanced interpretation of the results and ensures that conclusions are not overly reliant on a single predictive model, but are instead informed by a spectrum of computational insights, which increases confidence in the pathogenicity prediction of each genetic variant. In the future, additional *in vitro* or *in vivo* work is needed to confirm and validate the function of these genetic variants [111].

In conclusion, our meta-analyses identified 12 genetic variants in 10 candidate genes (*ABCC2* rs717620, *ABCG2* rs2231142, *CDA* rs1048977, *CYP1A1* rs1048943, *ERCC1* rs3212986, *ERCC2* rs1799793 and rs1052555, *GSTM1* rs36631, *XPC* rs77907221, *XRCC1* rs1799782 and rs25487, and *XRCC3* rs861539) that showed statistically significant associations with response to PBC in NSCLC patients with strong or moderate cumulative epidemiological evidence. We also identified 172 genetic variants that were not associated with PBC response in at least one genetic model. Our results provide the most up-to-date summary and field synopsis of the genetic variants associated with response to PBC in NSCLC patients.

## Summary Table

### What Is Known About This Subject

• Response to platinum-based chemotherapy (PBC) in patients with non-small cell lung cancer (NSCLC) is genetically influenced.
• Previous studies on genetic variants and response to PBC in NSCLC have led to inconsistent results.
• Several genes have been implicated, but their functional effects are unknown.

### What This Paper Adds

• A comprehensive meta-analysis of 121 publications was performed to summarize and provide the most up-to-date evidence on this topic.
• Twelve genetic variants from 10 candidate genes were identified to be significantly associated with PBC response in NSCLC.
• Bioinformatics analysis predicted potential functional effects for the XRCC1 rs1799782 and CYP1A1 rs1048943 variants.

## Concluding Statement

This work represents an advance in biomedical science because it offers the most comprehensive, up-to-date synthesis of genetic variants influencing platinum-based chemotherapy response in NSCLC patients.

## Data Availability

The original contributions presented in the study are included in the article/Supplementary Material, further inquiries can be directed to the corresponding author.

## Abbreviations

PBC, Platinum-based chemotherapy; SNP, Single nucleotide polymorphism; NSCLC, Non-small cell lung cancer; NOS, Newcastle-Ottawa Scale; RECIST, Response Evaluation Criteria in Solid Tumours; CADD, Combined Annotation Dependent Depletion; SIFTL, Sorting Intolerant From Tolerant; PolyPhen, Polymorphism Phenotyping; REVEL, Rare exome variant ensemble learner; ABCC2, ATP binding cassette Subfamily C Member 2; ABCG2, ATP binding cassette transporter G2; BAX, Bcl-2 associated protein; CDA, cytidine deaminase; CYP1A1, Cytochrome P450 1A1; ERCC1, Excision repair cross complementation group 1; ERCC2, Excision repair cross complementation group 2; ERCC2, Excision repair cross complementation group 5; GSTM1, Glutathione S-transferase 1; MTHFR, Methylenetetrahydrofolate reductase; MDR, Multidrug resistance mutation; XPC, Xeroderma pigmentosum complementation group C; XRCC1, X-ray cross-complementing protein 1; XRCC3, X-ray cross-complementing protein 3; UTR, untranslated region.
