# Supplementary material for: Genetic Variants Associated With Response to Platinum-Based Chemotherapy in Non-Small Cell Lung Cancer Patients: A Field Synopsis and Meta‐Analysis
Source: Br J Biomed Sci. 2024 Feb 21;81:11835. doi: 10.3389/bjbs.2024.11835 (PMC10914946; doi:10.3389/bjbs.2024.11835)
Supplement: Supplementary file 1 [file Table1.docx]

**Supplementary Table 1** Studies included for the meta-analysis and their characteristics

| **No.** | **Country** | **Ethnicity** | **SNPs studied** | **Sample Size** | **Genotyping method** | **Chemotherapy agents** | **Disease stage** | **References** |
| --- | --- | --- | --- | --- | --- | --- | --- | --- |
| 1 | China | Asian | *ERCC1* rs11615 | 142 | PCR + fluorescence probe | Cisplatin, paclitaxel, vinorelbine | III or IV | (122) |
| 2 | China | Asian | *ABCG2* rs2231142  *ABCG2* rs4148157  *ABCG2* rs2231164  *ABCG2* rs1871744 | 1004 | iSelect HD BeadChip | Cisplatin, navelbine, gemcitabine, paclitaxel, doxetaxel, others | III or IV | (123) |
| 3 | China | Asian | *BAG1* C324T  *XPD* rs13181  *XPD* rs1799793 | 142 | PCR-RFLP | Cisplatin, vinorelbine | IIIb or IV | (124) |
| 4 | China | Asian | *ERCC1* rs3212986  *ERCC1* rs11615  *XPD* rs13181 | 115 | 3D- polyacrylamide gel-based DNA microarray | Cisplatin, carboplatin, vinorelbine, gemcitabine | IIIb or IV | (125) |
| 5 | Korea | Asian | *ERCC1* rs11615  *XPD* rs13181  *XPD* rs1305686667 | 109 | PCR and ABI 3700 | Cisplatin, gemcitabine, paclitaxel, docetaxel | IIIb or IV | (126) |
| 6 | Korea | Asian | *ABCC2* rs717620  *ABCC2* rs2273697  *ABCC2* rs3740066 | 445 | Sequenom Mass ARRAY | Cisplatin, carboplatin vinorelbine gemcitabine, paclitaxel, docetaxel | III or IV | (127) |
| 7 | China | Asian | *CDC25* rs1380053  *CDC25* rs3731513 | 663 | iSelect HD Bead-Chip | Cisplatin, carboplatin, navelbine, gemicitabine, paclitaxel, etoposide, bevacizumab | III or IV | (128) |
| 8 | Italy | European | *CDA* rs10458977  *CDA* rs2072671  *RRM1* rs11030813  *ERCC1* rs11615  *XRCC3 rs861539*  *XPD* rs13181  *P53* rs1042522 | 192 | ABI Prism 7300 Sequence Detection System | Cisplatin, gemcitabine, vinorelbine | IIIb or IV | (86) |
| 9 | China | Asian | *OGG1* rs1052133  *APE1* rs1130409  *APE1* T141G  *XRCC1* rs25487 | 487 | PCR and agarose gel electrophoresis | Cisplatin, docetaxel, gemcitabine | III or IV | (129) |
| 10 | Japan | Asian | *TP53* rs1042522 | 640 | Pyrosequencing or Taqman | Cisplatin, carboplatin, paclitaxel, docetaxel, vinorelbine, gemcitabine, irinotecan | III or IV | (130) |
| 11 | China | Asian | *GSTP1* rs1695 | 262 | PCR-RFLP | Cisplatin-based chemotherapy | III or IV | (131) |
| 12 | China | Asian | *GSTP1* rs1695  *RRM1* rs11030813 | 47 | Sanger sequencing | Cisplatin, gemcitabine | III or IV | (132) |
| 13 | China | Asian | *GSTP1* rs1695  *XRCC1* rs1799782  *XRCC1* rs25489  *XRCC1* rs25487 | 325 | PCR-RFLP | Cisplatin, gemcitabine, vinorelbine, paclitaxel, docetaxel | IIIb or IV | (133) |
| 14 | China | Asian | *GSTP1* rs1695  *XRCC1* rs1799782  *XRCC1* rs25487 | 322 | PCR-RFLP | Cisplatin, gemcitabine, vinorelbine, doxetaxel | IIIb or IV | (134) |
| 15 | China | Asian | *GSTM1* rs36631  *GSTT1* rs17856199  *GSTP1* rs1695 | 282 | PCR-RFLP | Cisplatin-based chemotherapy | III or IV | (135) |
| 16 | China | Asian | *ERCC1* rs11615  *ABCB1* rs1045642  *ABCB1* E1/-129(T/C)  *ABCB1* rs2032582 | 95 | Ligase detection reactions using ABI sequencer 377 | Cisplatin, gemcitabine, vinorelbine, taxol | IIIb or IV | (136) |
| 17 | China | Asian | *POLK* rs3213801  *POLK* rs1018119  *POLK* rs10077427  *POLK* rs5744545  *POLK* rs3756558  *POLK* rs449106  *POLK* rs5744653  *POLK* rs3213801 | 663 | iPLEX | Cisplatin, carboplatin, navelbine, gemcitabine, docetaxel, taxol | III or IV | (137) |
| 18 | China | Asian | *CCAT2* rs6983267  *H19* rs2839698  *MALAT1* rs619586  *HOTAIR* rs7958904 | 467 | Sequenom Mass ARRAY | Platinum, gemcitabine, etoposide, docetaxel, paclitaxel, pemetrexed | I - IV | (138) |
| 19 | China | Asian | *XPD* rs1799793  *XPD* rs13181  *CDA* rs2072671 | 93 | PCR-RFLP | Cisplatin, gemcitabine | IIIb or IV | (139) |
| 20 | China | Asian | *ERCC1* rs11615  *ERCC1* rs3212986 | 90 | PCR + gel electrophoresis | Cisplatin-based chemotherapy | IIIb or IV | (140) |
| 21 | Korea | Asian | *ABCB1* rs1128503  *ABCB1* rs2032582  *ABCB1* rs2032582  *ABCC2* rs717620  *ABCC2* rs2273697  *ABCC2* rs3740066 *ABCG2* rs2231137  *ABCG2* rs2231142 | 107 | Taqman Assay | Cisplatin, irinotecan | IIIb or IV | (141) |
| 22 | China | Asian | *CDA* rs2072671  *CDA* rs60369023 | 120 | PCR gel electrophoresis sequencing | Cisplatin, gemcitabine | IIIb or IV | (142) |
| 23 | China | Asian | *OCT2* rs316003  *OCT2* rs316019  *ABCB1* rs1045642  *ABCC2* rs717620  *ABCC2* rs2273697  *ABCC2* rs3740066  *MATE1* rs2289669 | 403 | Sequenom Mass ARRAY | Cisplatin, carboplatin, gemcitabine, pemetrexed, paclitaxel, docetaxel, navelbine | I - IV | (143) |
| 24 | China | Asian | *ATP7B* rs1061472  *ATP7B* rs9535826 | 427 | Allele specific MALDI-ToF mass spectrometry | Cisplatin, carboplatin, paclitaxel, docetaxel, pemetrexed, navelbine | I - IV | (144) |
| 25 | China | Asian | *BMP4 G*5826A  *BMP4* C6007T | 938 | PCR and gel electrophoresis | Cisplatin, carboplatin, taxol, taxetere, docetaxel, gemcitabine, vinorelbine | III or IV | (145) |
| 26 | China | Asian | *GSTP1* rs1695  *ATP7A* rs2227291  *XRCC1* rs25487 | 97 | ABI Prism 3100 | Cisplatin, paclitaxel, vinorelbine, gemcitabine, paclitaxel | IIIb or IV | (146) |
| 27 | China | Asian | *CASC8* rs10505477 | 467 | Sequenom Mass ARRAY | Platinum, gemcitabine, etoposide, pemetrexed | I - IV | (147) |
| 28 | Taiwan | Asian | *ERCC1* rs11615  *ERCC1* rs3212986  *XPD rs13181*  *XPD* rs1799793  *XRCC1* rs25487  *XRCC3* rs861539 | 58 | ABI 7900 Sequence Detection System | Platinum, pemetrexed | IV | (148) |
| 29 | China | Asian | *XPD rs13181*  *XPD* rs1799793  *XPD* rs1052555  *XPD* rs238406 | 496 | Sequenom Mass ARRAY | Cisplatin, carboplatin, gemcitabine, navelbine | III or IV | (149) |
| 30 | China | Asian | *MTHFR* rs1801133 | 51 | PCR | Cisplatin, pemetrexed | IIIb or IV | (150) |
| 31 | Italy | European | *ERCC1* rs111615  *XPD* rs13181  *XPD* rs1799793  *CDA* rs2072671 | 65 | Taqman Assay | Cisplatin, gemcitabine | IIIb or IV | (151) |
| 32 | Italy | European | *CDA* rs2072671  *CDA* rs1048977 | 115 | Taqman Assay | Cisplatin, gemcitabine | IIIb or IV | (152) |
| 33 | Spain | European | *RRM1* rs12806698  *XPD* rs13181  *XPD* rs1799793 | 63 | ABI Prism 7900HT | Cisplatin, gemcitabine, docetaxel | IIIa and IIIb | (153) |
| 34 | China | Asian | *XPC* rs77907221  *XPC* rs13181  *ERCC1* rs3212986 | 200 | PCR-RFLP | Cisplatin, carboplatin, vinorelbine, paclitaxel, docetaxel | I - IV | (154) |
| 35 | China | Asian | *MSH3* rs26279  *MSH3* rs1650697  *MSH3* rs1105524 | 180 | SNaPShot Multiplex | Carboplatin, cisplatin, gemcitabine, navelbine, | IIIb or IV | (155) |
| 36 | China | Asian | *XRCC1* rs1799782  *XRCC1* rs25487 | 164 | PCR-RFLP | Cisplatin, vinorelbine | III or IV | (156) |
| 37 | Switzerland | European | *CCND1* rs9344 | 244 | PCR-RFLP | Cisplatin, carboplatin, paclitaxel, docetaxel, gemcitabine | I - IV | (157) |
| 38 | China | Asian | *CDA* rs1048977 | 163 | ABI 3730 Sequencing | Cisplatin, nedaplatin, gemcitabine | I - IV | (158) |
| 39 | China | Asian | *ERCC1* rs11615  *XPD* rs13181  *XRCC1* rs25487 | 89 | Taqman assay | Cisplatin, docetaxel, gemcitabine, vinorelbine | III or IV | (159) |
| 40 | Greece | European | *ERCC1* rs11615  *ERCC1* rs3212986  *XPD* rs13181  *XPD* rs1799793  *XRCC1* rs25487  *GSTP1* rs1695  *GSTM1* rs36631  *GSTT1* rs17856199 | 119 | PCR-RFLP | Platinum-based chemotherapy | III or IV | (160) |
| 41 | China | Asian | *ERCC1* rs11615  *ERCC1* rs3212986  *ERCC1* rs2298881 | 163 | Sequenom Mass ARRAY | Platinum-based chemotherapy | III or IV | (161) |
| 42 | China | Asian | *XPD* rs13181  *XPD* rs1799793  *XPD* rs1052555  *XPD* rs238406 | 353 | Sequenom Mass ARRAY iPLEX | Cisplatin, carboplatin, gemcitabine, taxol, docetaxel, navelbine | III or IV | (162) |
| 43 | China | Asian | *NF-κB* rs230521  *NF-κB* rs4648068  *PXR* rs3814058 | 262 | Mass ARRAY | Platinum-based chemotherapy | III or IV | (163) |
| 44 | Thailand | Asian | *ERCC1* rs11615 | 26 | Taqman assay | Cisplatin and carboplatin- based therapy | IIIb or IV | (164) |
| 45 | China | Asian | *XRCC1* rs25487  *XRCC1* rs1799782  *XRCC3* rs861539 | 130 | PCR-RFLP | Platinum-based chemotherapy | IIIb or IV | (165) |
| 46 | China | Asian | *XRCC1* rs17997982  *XRCC1* rs25489  *XRCC1* rs25487  *XRCC3* rs861539  *ERCC5* rs1047768  *ERCC5* rs17655 | 378 | PCR-RFLP | Cisplatin, gemcitabine, docetaxel, vinorelbine, paclitaxel | I - iV | (166) |
| 47 | Greece | European | *ERCC1* rs11615  *ERCC1* rs3212986  *XPD* rs13181  *XPD* rs1799793  *XRCC1* rs25487 | 107 | PCR-RFLP | Cisplatin, carboplatin, docetaxel, paclitaxel, gemcitabine | IIIb or IV | (167) |
| 48 | United Kingdom | European | *GSTP1* rs1695  *GSTP1* rs1138272 | 108 | PCR and direct sequencing | Cisplatin, carboplatin, mitomycin, ifosfamide, vinblastine, docetaxel | III or IV | (168) |
| 49 | China | Asian | *EPO* rs1617640 | 437 | Sequenom Mass ARRAY | Cisplatin, carboplatin, gemcitabine, pemetrexed,, paclitaxel, docetaxel navelbine | III or IV | (169) |
| 50 | China | Asian | *FGFR4* rs351855 | 629 | 3130 Sequence Platform | Platinum-based chemotherapy | III or IV | (170) |
| 51 | China | Asian | *LGALS3* rs4644  *LGALS3* rs4652 | 320 | RFLP and SSOPH | Platinum-based chemotherapy | III or IV | (171) |
| 52 | China | Asian | *ATP7B* rs9526814 | 247 | Sequenom Mass ARRAY | Cisplatin, carboplatin, gemcitabine, paclitaxel, navelbine, memetrexed, docetaxel | II - IV | (172) |
| 53 | China | Asian | *CTR1* rs7851395  *CTR1* rs12686377 | 282 | RFLP | Cisplatin, carboplatin, etoposide, vindeside, docetaxel, gemcitabine, vindesine | III or IV | (173) |
| 54 | Taiwan | Asian | *ERCC1* rs11615  *ERCC1* rs3212986  *XPD* rs13181  *XPD* rs1799793  *XRCC1* rs25487  *XRCC3* rs861539  *RRM1* rs9937  *RRM1* rs1042858 | 62 | SNPstream Genotyping System | Cisplatin, carboplatin, oxaliplatin, gemcitabine, bevacizumab | IIIb or IV | (174) |
| 55 | China | Asian | *ATM* rs664143  *ERCC1* rs11615  *APE1* rs1130409  *iASPP* A67T | 230 | Taqman assay | Cisplatin, vinorelbine, gemcitabine, taxol | III or IV | (175) |
| 56 | China | Asian | *GSTP1* rs1695  *XRCC1* rs25487 | 111 | ABI 3730XL Sequencer | Cisplatin, carboplatin, docetaxel, gemcitabine, vinorelbine, pemetrexed | IV | (176) |
| 57 | Bangladesh | Asian | *GSTP1* rs1695  *XRCC1* rs25487  *XPC* rs2228001  *ERCC1* rs11615 | 285 | PCR-RFLP | Cisplatin, carboplatin, gemcitabine, vinorelbine, paclitaxel | IIIb or IV | (177) |
| 58 | China | Asian | *AKT1* rs2498786  *AKT1* rs2494752  *AKT1* rs2494750  *AKT1* rs74090038  *AKT2* rs34716810  *AKT2* rs62107593  *FRAP1* rs12139042 | 199 | TaqMan assays using ABI 7900 | Cisplatin, carboplatin, paclitaxel, tanetere, docetaxel, gemcitabine, vinorelbine | III or IV | (178) |
| 59 | China | Asian | *NBS1* rs1805794  *NBS1* rs13312840 | 147 | TaqMan Assays | Cisplatin, carboplatin, paclitaxel, tanetere, docetaxel, gemcitabine, vinorelbine | IIIb or IV | (179) |
| 60 | China | Asian | *MTHFR* rs1801133  *MTHFR* rs1801131 | 97 | PCR-RFLP | Platinum, vinorelbine, paclitaxel/ docetaxel, gemcitabine | II - IV | (180) |
| 61 | China | Asian | *GSTP1* rs1695  *XRCC1* rs25487  *XRCC1* rs1799782 | 141 | PCR-RFLP | Cisplatin-based chemotherapy | III or IV | (181) |
| 62 | China | Asian | *MTHFR* rs1537514  *MTHFR* rs1801131  *MTHFR* rs1801133 | 976 | iSelect BeadChip | Cisplatin, carboplatin, vinorelbine, gemcitabine, paclitaxel, docetaxel | III or IV | (182) |
| 63 | China | Asian | *ABCB1* rs1045642 | 100 | RT-PCR | Platinum, gemcitabine, docetaxel, paclitaxel, vinorelbine | IIIb or IV | (183) |
| 64 | China | Asian | *MTHFR* rs1801133  *MTR* rs9979 | 438 | RT-PCR | Cisplatin, carboplatin, vinorelbine, gemcitabine, taxol, docetaxel | I - IV | (184) |
| 65 | China | Asian | *ERCC1* rs3212986 | 300 | RT-PCR | Cisplatin, carboplatin, vinorelbine, paclitaxel, gemcitabine | IIIb or IV | (185) |
| 66 | China | Asian | *MMP2* rs12934241 | 663 | iSelect HD BeadChip | Cisplatin, carboplatin, gemcitabine, paclitaxel, docetaxel | III or IV | (186) |
| 67 | China | Asian | *ABCB1* rs2032582  *ABCB1* rs1045642 | 54 | ABI model 377 | Cisplatin,  docetaxel | IIIb or IV | (187) |
| 68 | China | Asian | *CYP3A5*3* A22893G  *ABCB1* rs2032582  *ABCB1* rs1045642  *COX2* rs689466 | 69 | ABI model 377 | Cisplatin,  Vinorelbine | IIIb or IV | (188) |
| 69 | China | Asian | *ERCC1* rs11615  *ERCC1* rs3212986  *MTHFR* rs1801133 | 135 | ABI 7500 RT-PCR | Cisplatin, gemcitabine, carboplatin | IIIb or IV | (189) |
| 70 | China | Asian | *ABCC2* rs717620  *ABCC2* rs2273697  *ABCC2* rs3740066  *GSTP1* rs1695 | 113 | 3D polyacrylamide gel-based DNA microarray | Cisplatin, carboplatin, paclitaxel, taxol, taxetere, docetaxel, gemcitabine, vinorelbine | III or IV | (190) |
| 71 | China | Asian | *OPN* rs11730582  *OPN* T66G | 376 | Taqman | Cisplatin, paclitaxel | IIIb or IV | (191) |
| 72 | Korea | Asian | *PD-L1* rs2297136  *PD-L1* rs4143815 | 379 | Sequenom Mass ARRAY iPLEX assay | Cisplatin, paclitaxel | III or IV | (192) |
| 73 | China | Asian | *XRCC1* rs1799782  *XRCC1* rs25487  *XPD* rs13181  *XPD* rs1799793  *XPD* rs1052555  *XPD* rs238406 | 375 | Sequenom Mass ARRAY | Carboplatin, cisplatin, navelbine, docetaxel, taxol | III or IV | (193) |
| 74 | Spain | European | *ERCC1* rs11615  *ERCC1* rs3212986  *ERCC1* rs3212948  *XPD* rs1799793  *XPD* rs13181  *ERCC3* rs4150454  *ERCC3* rs4150402  *ERCC3* rs3738948  *ERCC4* rs1799801  *ERCC5* rs1047768  *ERCC5* rs17655  *XPA* rs1800975  *XRCC1* rs25487  *XRCC1* rs25489  *XRCC1* rs1799782  *XRCC1* rs3213239  *XRCC2* rs3218536 | 161 | TaqMan | Platinum, gemcitabine, vinorelbine, taxane, pemetrexed | III or IV | (194) |
| 75 | China | Asian | *REV3* rs240969  *REV3* rs456865  *REV3* rs4945880  *REV3* rs3218573  *REV7* rs2336030 | 663 | Sequenom iPLEX | Cisplatin, carboplatin, taxol, docetaxel, navelbine, gemcitabine | III or IV | (195) |
| 76 | Korea | Asian | *XRCC1* rs25487  *ERCC2* rs1052555  *BRCA1* rs79917  *TNFRSF1B* rs1061624  *BCL2* rs2279115  *BIRC5* rs9904341 | 382 | PCR-RFLP | Cisplatin, paclitaxel | III or IV | (196) |
| 77 | China | Asian | *ERCC1* rs11615  *ERCC1* rs3212986  *ERCC1* rs2298881  *ERCC4* rs2276465  *ERCC4* rs6498486 | 250 | PCR-RFLP | Cisplatin, vinorelbine, gemcitabine, paclitaxel, docetaxel | III or IV | (197) |
| 78 | China | Asian | *ERCC1* rs11615  *ERCC1* rs3212986  *ERCC1* rs2298881 | 192 | Sequenom Mass ARRAY | Platinum-based chemotherapy | III or IV | (198) |
| 79 | Korea | Asian | *PFKL* rs2073436  *GPI* rs7248411 | 377 | Sequenom Mass ARRAY iPLEX | Cisplatin, paclitaxel | III or IV | (199) |
| 80 | China | Asian | *GSTM1* rs36631  *CYP1A1* rs1048943  *CYP2E1 Rsa1*  *CYP2D6* rs1065852 | 217 | PCR-RFLP | Platinum-based chemotherapy | III or IV | (200) |
| 81 | China | Asian | *hMSH2 gIVS12-6*  *hMLH1-1151* | 96 | PCR and 3D polyacrylamide gel-based DNA microarray | Cisplatin, carboplatin, vinorelbine, gemcitabine, taxol, docetaxel | III or IV | (201) |
| 82 | China | Asian | *XPC* rs77907221  *XPD* rs13181 | 151 | PCR-RFLP | Platinum-based chemotherapy | IIIb or IV | (202) |
| 83 | China | Asian | *XRCC1* rs25487  *XRCC1* rs1799782  *PARP1* rs1136410  *APE1* rs1130409 | 147 | TaqMan | Cisplatin, carboplatin, taxol, Taxotere, docaetaxel,  Gemcitabine, vinorelbine | IIIb or IV | (203) |
| 84 | Poland | European | *CASP8* rs3769818 | 99 | RT-PCR | Cisplatin, pemetrexed, gemcitabine, vinorelbine | IIIb or IV | (204) |
| 85 | China | Asian | *TS* rs45445694  *MTHFR* rs18001133  *SCL19A1* rs1051298 | 45 | ABI Prism 3730 DNA analyzer, ABI 7900 HT Sequence Detector | Cisplatin, pemetrexed | IIIb or IV | (205) |
| 86 | Poland | European | *MTHFR* rs1801133  *ERCC1* rs11615 | 115 | PCR RFLP | Cisplatin, carboplatin, pemetrexed | II - IV | (206) |
| 87 | China | Asian | *XRCC1* rs25487  *XRCC1* rs1799782  *ERCC4* rs7655  *ERCC4* rs1047768 | 82 | PCR and 3D DNA microarray | Cisplatin, carboplatin, vinorelbine, taxol, docetaxel | IV | (207) |
| 88 | China | Asian | *BCL2* rs2279115  *BAX* rs4645878 | 180 | PCR-RFLP | Cisplatin, taxol, docetaxel, gemcitabine, vionorelbine, pemetrexed | III or IV | (208) |
| 89 | China | Asian | *TP53* rs1042522  *MDM2* rs2279744  *MDM2* rs937282 | 444 | PCR-RFLP | Platinum, navelbine, gemcitabine, paclitaxel, docetaxel | III or IV | (209) |
| 90 | China | Asian | *RRM1* rs12806698  *RRM1* rs11030813 | 214 | PCR-RFLP | Cisplatin, carboplatin, oxaliplatin navelbine, paclitaxel, gemcitabine, etoposide, ifosfamide | IIb - IV | (210) |
| 91 | China | Asian | *GSTM1* rs36631  *GSTT1* rs17856199  *GSTP1* rs1695 | 262 | PCR-RFLP | Cisplatin-based chemotherapy | III or IV | (211) |
| 92 | Poland | European | *ERCC1* rs11615  *RRM1* rs12806698 | 62 | PCR-RFLP | Cisplatin, carboplatin, gemcitabine | III or IV | (36) |
| 93 | Poland | European | *ERCC1* rs11615 | 43 | PCR-RFLP | Cisplatin-based chemotherapy | IIIb or IV | (212) |
| 94 | Poland | European | *ERCC1* rs11615  *ERCC1* rs3212986  *XPD* rs13181  *XPD* rs1799793  *XPC* C1385T  *XPC* C2704A  XPA A4G  *ERCC5* rs17655 | 91 | SNaPshot PCR | Cisplatin, gemcitabine | IIIb or IV | (73) |
| 95 | Poland | European | *STMN1* T2166C | 110 | SNaPshot PCR | Platinum-based chemotherapy | IIIb or IV | (213) |
| 96 | China | Asian | *COX-2* rs689465  *COX-2* rs689466  *COX-2* rs3218625  *COX-2* rs20417 | 190 | PCR-RFLP | Cisplatin, carboplatin, vinorelbine, gemcitabine, taxol/ 0docetaxel, navelbine | IIIb or IV | (214) |
| 97 | Spain | European | *XPD* rs1799793  *XPD* rs13181  *XRCC3*  *AURORA* rs2273535  *AURORA* rs1047972 | 180 | Taqman assay | Cisplatin, vinorelbine | IIIb or IV | (215) |
| 98 | China | Asian | *PTEN* rs11202607  *PTEN* rs701848  *PTEN* rs11202592 | 111 | Taqman assay | Cisplatin, carboplatin, navelbine, gemcitabine, paclitaxel, docetaxel | III or IV | (216) |
| 99 | China | Asian | *RAGE* T374A  *RAGE* rs2070600  *RAGE* T249C | 432 | PCR and gel electrophoresis | Cisplatin, carboplatin, docetaxel, gemcitabine, navelbine, pemetrexed | I - IV | (217) |
| 100 | China | Asian | *ERCC1* rs11615  *XPD* rs13181  *BRCA1* rs1799966 | 124 | Taqman Assay | Cisplatin, carboplatin, gemcitabine, paclitaxel, docetaxel, vinorelbine, pemetrexed | III or IV | (218) |
| 101 | China | Asian | *GSTM1* rs36631  *CYP1A1* rs1048943  *CYP2E1*  *CYP2D6* rs1065852 | 76 | PCR-RFLP | Platinum-based chemotherapy | All stages | (219) |
| 102 | China | Asian | *IGF-1R* G1013A  *IGF-1R* G1619A | 132 | PCR-RFLP | Cisplatin, carboplatin, gemcitabine, vinorelbine, paclitaxel, docetaxel, pemetrexed | III or IV | (220) |
| 103 | China | Asian | *ERCC1* rs11615  *ERCC1* rs3212986  *ERCC1* rs3212948  *ERCC1* rs229881  *ERCC1* rs226466  *ERCC1* rs6498486 | 187 | Sequenom Mass ARRAY | Platinum-based chemotherapy | III or IV | (221) |
| 104 | China | Asian | *ERCC5* rs2016073  *ERCC5* rs4771436  *ERCC5* rs11069498  *ERCC5* rs4150330  *ERCC5* rs873601  *ERCC* rs20106073  *ERCC5* rs4771436  *ERCC5* rs11069498  *ERCC5* rs4150330  *ERCC5* rs873601 | 189 | Agena Mass ARRAY | Cisplatin-based chemotherapy | III or IV | (222) |
| 105 | China | Asian | *ERCC5* rs2094258  *ERCC5* rs751402  *ERCC5* rs2296147 | 228 | PCR-RFLP | Cisplatin, carboplatin, vinorelibine | III or IV | (223) |
| 106 | China | Asian | *GSTM1* rs36631  *GSTT1* rs17856199  *GSTP* rs1695 | 244 | PCR-RFLP | Cisplatin-based chemotherapy | III or IV | (224) |
| 107 | China | Asian | *GSTP1* rs1695  *XRCC1* rs1799782  *XRCC1* rs25489  *XRCC1* rs25487 | 206 | PCR-RFLP | Cisplatin-based chemotherapy | III or IV | (225) |
| 108 | China | Asian | *XRCC1* rs1799782  *XRCC1* rs25487  *ERCC5* rs1047768  *ERCC5* rs17655 | 378 | PCR-RFLP | Cisplatin, gemcitabine, docetaxel, vinorelbine, paclitaxel | III or IV | (226) |
| 109 | China | Asian | *RRM1* rs12806698  *RRM1* rs9937  *RRM1* rs1042858 | 62 | RT-PCR | Carboplatin, gemcitabine | III or IV | (227) |
| 110 | Spain | European | *ERCC1* rs11615  *XPD* rs13181  *RRM1* rs12806698  *ABCB1* rs1045642 | 60 | Taqman assay | Cisplatin, docetaxel | IIIb or IV | (228) |
| 111 | Spain | European | *XPD* rs13181  *XPD* rs1799793  *ERCC1* rs11615  *RRM1* rs12806698  *ABCB1* rs1045642 | 94 | ABI Prism 7000 | Cisplatin, vinorelbine | IIIb or IV | (229) |
| 112 | China | Asian | *XRCC1* rs1799782  *XRCC1* rs25487 | 105 | PCR--RFLP | Cisplatin, carboplatin, vinorelbine, paclitaxel, docetaxel | IIIb or IV | (230) |
| 113 | China | Asian | *ABCB1* rs1045642  *ABCB1* rs1128503  *ABCG2* rs2231142  *ABCC2* rs3740066  *ABCC2* rs717620 | 240 | Mass ARRAY | Cisplatin, carboplatin- based chemotherapy | III or IV | (100) |
| 114 | China | Asian | *NBS* rs1805794  *LIG4* rs1805388  *RAD51* rs180132 | 146 | PCR-RFLP | Cisplatin, carboplatin, paclitaxel, gemcitabine,  pemetrexed | IIIb or IV | (231) |
| 115 | China | Asian | *BAG1* C324T | 120 | RT-PCR | Cisplatin, vinorelbine | I - IIIA | (232) |
| 116 | China | Asian | *FOKI* rs10735810  *BSMI* rs1544410  *APAI* rs7975232  *TAQI* rs10735810 | 755 | PCR-RFLP | Cisplatin/carboplatin, taxol/paclitaxel/docetaxel, gemcitabine, vinorelbine | III or IV | (233) |
| 117 | China | Asian | *ERCC5* rs1047768  *XPA* rs1800975 | 115 | Gel-based DNA microarray | Cisplatin, carboplatin, gemcitabine, vinorelbine, taxane, paclitaxel, docetaxel | III or IV | (234) |
| 118 | China | Asian | *XPC* rs2228001  *XPC* C11A  *XPC* rs2228000 | 164 | PCR and ABI377 sequencing | Cisplatin, vinorelbine | III or IV | (235) |
| 119 | India | Asian | *XPC* rs2228001  *XPC* rs2228000 | 167 | PCR-RFLP | Platinum-based chemotherapy | I - IV | (236) |
| 120 | China | Asian | *XPC* rs2228001  *XPC* rs2228000 | 96 | 3D polyacrylamide gel-based DNA microarray | Cisplatin, carboplatin, taxol, docetaxel, gemcitabine, vinorelbine | III or IV | (237) |
| 121 | China | Asian | *ERCC5* rs2296147  *ERCC5* rs4150261  *ERCC5* rs17655  *ERCC5* rs1047768  *ERCC5* rs2094258 | 451 | Taqman RT-PCR | Cisplatin, carboplatin, vinorelbine, gemcitabine, docetaxel | III or IV | (238) |
