# Supplementary material for: Genetic Variants Associated With Response to Platinum-Based Chemotherapy in Non-Small Cell Lung Cancer Patients: A Field Synopsis and Meta‐Analysis
Source: Br J Biomed Sci. 2024 Feb 21;81:11835. doi: 10.3389/bjbs.2024.11835 (PMC10914946; doi:10.3389/bjbs.2024.11835)
Supplement: Supplementary file 2 [file Table2.docx]

**Supplementary Table 2** Genetic variants significantly associated with response to PBC in NSCLC patients

| **Genes** | **Variants** | **Quality of the included studies (NOS)** | **Ethnicity** | **Number evaluated** | | **Genetic model** | **Genetic associations with PBC response** | | **Heterogeneity** | |  |  |  |  |
| --- | --- | --- | --- | --- | --- | --- | --- | --- | --- | --- | --- | --- | --- | --- |
|  |  |  |  | **Studies** | **Cases/controls** |  | **OR (95% CI)** | **p-value** | **I2 (%)** | **P (Q test)** | **Begg P** | **Egger P** | **Venice criteria grades** | **Credibility of evidence** |
| *ABCB1* | rs2032582 (G>T/A) | Good/Poor | Asian | 4 | 166/143 | Dominant | 1.822 (1.095-3.030) | 0.0210 | 41.1 | 0.165 | 0.174 | 0.332 | CAA | Weak |
|  |  |  |  | 4 | 166/143 | Heterozygous | 1.959 (1.112-3.322) | 0.0190 | 24.9 | 0.262 | 0.174 | 0.252 | CAA | Weak |
| *ABCC2* | rs717620 (C/T) | Good/Poor | Asian | 3 | 344/110 | Allele | 0.044 (0.008-0.241) | 0.0001 | 69.4 | 0.038 | 0.117 | 0.098 | BBA | Moderate |
| *ABCG2* | rs2231142 (C/A) | Good/Poor | Asian | 3 | 1059/256 | Recessive | 0.480 (0.316-0.727) | 0.0010 | 0.0 | 0.840 | 0.117 | 0.400 | AAB | Moderate |
|  |  |  |  | 3 | 1059/256 | Homozygous | 0.477 (0.306-0.741) | 0.0010 | 0.0 | 0.914 | 0.117 | 0.205 | AAB | Moderate |
|  |  |  |  | 3 | 1059/256 | Allele | 0.754 (0.612-0.929) | 0.0080 | 0.0 | 0.992 | 0.602 | 0.641 | AAB | Moderate |
|  | rs2231164 (G/A) | Good | Asian | 1 | 798/177 | Dominant | 1.584 (1.099-2.283) | 0.0140 | - | - | - | - | BCC | Weak |
|  |  |  |  | 1 | 798/177 | Heterozygous | 1.682 (1.132-2.499) | 0.0100 | - | - | - | - | BCC | Weak |
|  | rs1871744 (A/G) | Good | Asian | 1 | 798/177 | Dominant | 1.519 (1.093-2.110) | 0.0130 | - | - | - | - | BCC | Weak |
|  |  |  |  | 1 | 798/177 | Heterozygous | 1.664 (1.157-2.394) | 0.0060 | - | - | - | - | BCC | Weak |
|  | rs4148157 (G/A) | Good | Asian | 1 | 798/177 | Recessive | 0.503 (0.283-0.895) | 0.0190 | - | - | - | - | CCC | Weak |
|  |  |  |  | 1 | 798/177 | Homozygous | 0.472 (0.260-0.858) | 0.0140 | - | - | - | - | CCC | Weak |
|  |  |  |  | 1 | 798/177 | Allele | 0.767 (0.596-0.987) | 0.0390 | - | - | - | - | BCC | Weak |
| *AKT1* | rs34716810 (C/T) | Good | Asian | 1 | 156/40 | Recessive | 0.359 (0.139-0.930) | 0.0350 | - | - | - | - | CCC | Weak |
| *APA1* | rs7975232 (G/T) | Good | Asian | 1 | 434/321 | Dominant | 1.507 (1.102-2.061) | 0.0100 | - | - | - | - | BCC | Weak |
|  |  |  |  | 1 | 434/321 | Recessive | 1.653 (1.123-2.433) | 0.0110 | - | - | - | - | BCC | Weak |
|  |  |  |  | 1 | 434/321 | Homozygous | 2.008 (1.296-3.111) | 0.0020 | - | - | - | - | BCC | Weak |
|  |  |  |  | 1 | 434/321 | Allele | 1.387 (1.128-1.705) | 0.0200 | - | - | - | - | BCC | Weak |
| *APQ2* | rs7314734 (C/T) | Good | Asian | 1 | 154/184 | Dominant | 6.715 (4.341-10.388) | 0.0001 | - | - | - | - | BCC | Weak |
|  |  |  |  | 1 | 154/184 | Allele | 3.944 (2.674-5.817) | 0.0001 | - | - | - | - | BCC | Weak |
|  |  |  |  | 1 | 154/184 | Heterozygous | 6.881 (4.428-10.693) | 0.0001 | - | - | - | - | BCC | Weak |
| *ATP7B* | rs9526814 (T/G) | Good | Asian | 1 | 86/62 | Recessive | 0.401 (0.161-0.997) | 0.0490 | 0.0 | - | - | - | CCC | Weak |
| *BAG1* | C324T | Good | Asian | 2 | 192/92 | Dominant | 2.786 (1.464-5.300) | 0.0020 | 0.0 | 1.000 | 1.000 | 0.000 | BAC | Weak |
|  |  |  |  | 1 | 96/46 | Heterozygous | 2.786 (1.222-6.918) | 0.0270 | - | - | - | - | CCC | Weak |
|  |  |  |  | 1 | 96/46 | Allele | 2.429 (1.029-5.734) | 0.0430 | - | - | - | - | CCC | Weak |
| *BAX* | rs4645878 (G/A) | Good | Asian | 1 | 151/84 | Recessive | 0.422 (0.201-0.887) | 0.0230 | - | - | - | - | CCC | Weak |
|  |  |  |  | 1 | 151/84 | Heterozygous | 1.951 (1.086-3.504) | 0.0250 | - | - | - | - | CCC | Weak |
| *BMP4* | C6007T | Good | Asian | 1 | 574/364 | Dominant | 2.093 (1.557-2.814) | 0.0001 | - | - | - | - | BCC | Weak |
|  |  |  |  | 1 | 574/364 | Recessive | 1.903 (1.389-2.608) | 0.0001 | - | - | - | - | BCC | Weak |
|  |  |  |  | 1 | 574/364 | Homozygous | 2.776 (1.911-4.032) | 0.0001 | - | - | - | - | BCC | Weak |
|  |  |  |  | 1 | 574/364 | Heterozygous | 1.807 (1.317-2.479) | 0.0001 | - | - | - | - | BCC | Weak |
|  |  |  |  | 1 | 574/364 | Allele | 1.703 (1.412-2.055) | 0.0001 | - | - | - | - | BCC | Weak |
| *BRCA1* | rs179966 (A/G) | Good | Asian | 1 | 41/83 | Dominant | 0.337 (0.155-0.732) | 0.0060 | - | - | - | - | CCC | Weak |
|  |  |  |  | 1 | 41/83 | Heterozygous | 0.298 (0.132-0.677) | 0.0040 | - | - | - | - | CCC | Weak |
|  | rs799917 (C/T) | Good | Asian | 1 | 192/179 | Dominant | 0.620 (0.411-0.936) | 0.0230 | - | - | - | - | BCC | Weak |
| *CASC8* | rs10505477 (A/G) | Good | Asian | 1 | 274/187 | Dominant | 1.559 (1.048-2.318) | 0.0280 | - | - | - | - | BCC | Weak |
|  |  |  |  | 1 | 274/187 | Heterozygous | 1.694 (1.110-2.585) | 0.0140 | - | - | - | - | BCC | Weak |
| *CCAT2* | rs6983267 (G/T) | Good | Asian | 1 | 179/268 | Recessive | 1.614 (1.077-2.420) | 0.0200 | - | - | - | - | BCC | Weak |
| *CCND1* | rs9344 (A/G) | Poor | European | 1 | 110/34 | Recessive | 3.077 (1.002-9.444) | 0.0500 | - | - | - | - | CCC | Weak |
|  |  |  |  | 1 | 110/34 | Homozygous | 4.211 (1.158-15.312) | 0.0290 | - | - | - | - | CCC | Weak |
|  |  |  |  | 1 | 110/34 | Allele | 1.811 (1.044-3.144) | 0.0350 | - | - | - | - | BCC | Weak |
| *CDA* | rs1048977 (C/T) | Good | Asian/European | 2 | 199/69 | Recessive | 0.181 (0.034-0.965) | 0.0450 | 51.0 | 0.153 | 0.317 | - | CBB | Weak |
|  |  |  |  | 2 | 199/69 | Allele | 0.516 (0.342-0.778) | 0.0200 | 32.8 | 0.222 | 0.317 | - | BBB | Moderate |
| *COX-2* | rs20417 (G/C) | Good | Asian | 1 | 126/64 | Dominant | 0.043 (0.022-0.085) | 0.0001 | - | - | - | - | CCC | Weak |
|  |  |  |  | 1 | 126/64 | Heterozygous | 0.417 (0.178-0.964) | 0.0450 | - | - | - | - | BCC | Weak |
| *CTR1* | rs12687377 (G/T) | Good | Asian | 1 | 142/140 | Recessive | 2.272 (1.247-4.139) | 0.0070 | - | - | - | - | CCC | Weak |
| *CYP1A1* | rs1048943 (T/C) | Good | Asian | 2 | 110/162 | Dominant | 2.593 (1.535-4.381) | 0.0001 | 0.0 | 0.905 | 0.317 | - | BAB | Moderate |
|  |  |  |  | 2 | 110/162 | Homozygous | 2.812 (1.377-5.741) | 0.0050 | 0.0 | 0.933 | 0.317 | - | CAB | Weak |
|  |  |  |  | 2 | 110/162 | Heterozygous | 2.512 (1.437-4.392) | 0.0010 | 0.0 | 0.846 | 0.317 | - | BAB | Moderate |
|  |  |  |  | 2 | 110/162 | Allele | 1.851 (1.303-2.631) | 0.0010 | 0.0 | 0.962 | 0.317 | - | BAB | Moderate |
| *EPO* | rs1617640 (G/T) | Good | Asian | 1 | 97/174 | Recessive | 0.507 (0.305-0.842) | 0.0090 | - | - | - | - | BCC | Weak |
|  |  |  |  | 1 | 97/174 | Allele | 0.554 (0.360-0.853) | 0.0070 | - | - | - | - | BCC | Weak |
| *ERCC1* | rs3212986 (C/A) | Good | Asian/European | 10 | 836/605 | Recessive | 1.685 (1.167-2.433) | 0.0050 | 33.2 | 0.152 | 0.677 | 0.257 | BAA | Moderate |
|  |  |  |  | 9 | 795/555 | Homozygous | 1.980 (1.346-2.913) | 0.0010 | 43.7 | 0.077 | 0.677 | 0.180 | BBA | Moderate |
|  |  |  |  | 10 | 836/605 | Allele | 1.417 (1.052-1.910) | 0.0220 | 66.2 | 0.020 | 0.180 | 0.331 | BBA | Moderate |
| *ERCC2 (XPD)* | rs1799793 (G/A) | Good | Asian/European | 7 | 533/466 | Recessive | 1.714 (1.068-2.751) | 0.0260 | 3.8 | 0.397 | 0.293 | 0.227 | CAA | Weak |
|  |  |  |  | 7 | 533/466 | Homozygous | 2.001 (1.216-3.294) | 0.0060 | 24.7 | 0.240 | 0.099 | 0.081 | CAA | Weak |
|  |  |  |  | 10 | 677/531 | Allele | 1.311 (1.082-1.590) | 0.0060 | 6.6 | 0.381 | 0.788 | 0.641 | BAA | Moderate |
|  |  |  |  | 17 | 1743/1039 | Dominant | 1.186 (1.000-1.407) | 0.0490 | 0.4 | 0.448 | 0.934 | 0.442 | AAA | Strong |
|  | rs1052555 (C/T) | Good | Asian | 3 | 811/346 | Dominant | 1.473 (1.063-2.042) | 0.0200 | 46.9 | 0.152 | 0.117 | 0.041 | BBB | Moderate |
| *ERCC3* | rs4150402 (T/C) | Good | European | 1 | 72/89 | Dominant | 0.427 (0.217-0.839) | 0.0140 | - | - | - | - | CCC | Weak |
|  |  |  |  | 1 | 72/89 | Recessive | 0.249 (0.127-0.491) | 0.0001 | - | - | - | - | CCC | Weak |
|  |  |  |  | 1 | 72/89 | Homozygous | 0.226 (0.102-0.502) | 0.0001 | - | - | - | - | CCC | Weak |
|  |  |  |  | 1 | 72/89 | Allele | 0.370 (0.239-0.574) | 0.0001 | - | - | - | - | BCC | Weak |
|  | rs4150454 (T/C) | Good | European | 1 | 72/89 | Recessive | 0.454 (0.227-0.908) | 0.0260 | - | - | - | - | CCC | Weak |
|  |  |  |  | 1 | 72/89 | Homozygous | 0.385 (0.179-0.831) | 0.0150 | - | - | - | - | CCC | Weak |
|  |  |  |  | 1 | 72/89 | Allele | 0.552 (0.362-0.842) | 0.0060 | - | - | - | - | BCC | Weak |
| *ERCC4* | rs1799801 (T/C) | Good | European | 1 | 72/89 | Heterozygous | 0.355 (0.150-0.843) | 0.0190 | - | - | - | - | CCC | Weak |
| *ERCC5 (XPG)* | rs11069498 (A/G) | Good | Asian | 1 | 100/42 | Recessive | 0.455 (0.216-0.957) | 0.0380 | - | - | - | - | CCC | Weak |
|  |  |  |  | 1 | 100/42 | Allele | 0.515 (0.280-0.947) | 0.0330 | - | - | - | - | CCC | Weak |
|  | rs751402 (A/G) | Good | Asian | 1 | 135/92 | Heterozygous | 3.095 (1.119-8.560) | 0.0290 | - | - | - | - | CCC | Weak |
|  |  |  |  | 1 | 135/92 | Dominant | 2.743 (1.037-7.258) | 0.0420 | - | - | - | - | CCC | Weak |
| *FGFR4* | rs351855 (G/A) | Good | Asian | 1 | 338/291 | Recessive | 0.281 (0.177-0.443) | 0.0001 | - | - | - | - | BCC | Weak |
|  |  | Good | Asian | 1 | 338/291 | Homozygous | 0.350 (0.210-0.582) | 0.0001 | - | - | - | - | BCC | Weak |
| *GPI* | rs7248411 (C/G) | Good | Asian | 1 | 193/174 | Allele | 0.673 (0.465-0.976) | 0.0370 | - | - | - | - | BCC | Weak |
| *GSTM1* | rs36631 (deletion) | Good | Asian | 5 | 567/439 | Allele | 0.531 (0.411-0.687) | 0.0001 | 0.0 | 0.412 | 0.050 | 0.011 | BAB | Moderate |
| *HOTAIR* | rs7598904 (C/G) | Good | Asian | 1 | 184/279 | Recessive | 0.613 (0.420-0.893) | 0.0110 | - | - | - | - | BCC | Weak |
| *LGALS3* | rs4652 (A/C) | Good | Asian | 1 | 201/119 | Dominant | 2.524 (1.570-4.057) | 0.0001 | - | - | - | - | BCC | Weak |
|  |  |  |  | 1 | 201/119 | Recessive | 1.893 (1.080-3.318) | 0.0260 | - | - | - | - | CCC | Weak |
|  |  |  |  | 1 | 201/119 | Homozygous | 2.913 (1.566-5.418) | 0.0010 | - | - | - | - | CCC | Weak |
|  |  |  |  | 1 | 201/119 | Heterozygous | 2.320 (1.372-3.922) | 0.0020 | - | - | - | - | BCC | Weak |
|  |  |  |  | 1 | 201/119 | Allele | 1.960 (1.407-2.730) | 0.0001 | - | - | - | - | BCC | Weak |
| *LIG4* | rs1805388 (C/T) | Good | Asian | 1 | 82/63 | Dominant | 2.056 (1.022-4.133) | 0.0430 | - | - | - | - | CCC | Weak |
| *MLH* | T1151A | Good | Asian | 1 | 68/28 | Dominant | 0.183 (0.056-0.591) | 0.0500 | - | - | - | - | CCC | Weak |
|  |  |  |  | 1 | 68/28 | Heterozygous | 0.183 (0.056-0.591) | 0.0500 | - | - | - | - | CCC | Weak |
|  |  |  |  | 1 | 68/28 | Allele | 0.214 (0.070-0.654) | 0.0070 | - | - | - | - | CCC | Weak |
| *MSH2* | gIVS112-6 | Good | Asian | 1 | 68/28 | Recessive | 0.090 (0.010-0.841) | 0.0350 | - | - | - | - | CCC | Weak |
|  |  |  |  | 1 | 68/28 | Homozygous | 0.071 (0.007-0.697) | 0.0230 | - | - | - | - | CCC | Weak |
|  |  |  |  | 1 | 68/28 | Allele | 0.472 (0.240-0.927) | 0.0290 | - | - | - | - | CCC | Weak |
| *MSH3* | rs1105524 (A/G) | Good | Asian | 1 | 103/77 | Recessive | 2.684 (1.180-6.108) | 0.0190 | - | - | - | - | CCC | Weak |
|  |  |  |  | 1 | 103/77 | Homozygous | 2.842 (1.177-6.682) | 0.0200 | - | - | - | - | CCC | Weak |
|  |  |  |  | 1 | 103/77 | Allele | 1.679 (1.087-2.593) | 0.0200 | - | - | - | - | BCC | Weak |
|  | rs1650697 (A/G) | Good | Asian | 1 | 102/77 | Dominant | 0.308 (0.125-0.756) | 0.0100 | - | - | - | - | CCC | Weak |
|  |  |  |  | 1 | 102/77 | Homozygous | 0.318 (0.123-0.820) | 0.0180 | - | - | - | - | CCC | Weak |
|  |  |  |  | 1 | 102/77 | Heterozygous | 0.297 (0.113-0.778) | 0.0140 | - | - | - | - | CCC | Weak |
|  |  |  |  | 1 | 102/77 | Allele | 0.615 (0.395-0.956) | 0.0310 | - | - | - | - | BCC | Weak |
|  | rs26279 (G/A) | Good | Asian | 1 | 103/77 | Heterozygous | 0.085 (0.010-0.708) | 0.0230 | - | - | - | - | CCC | Weak |
| *MTHFR* | rs1537514 (G/C) | Good | Asian | 1 | 757/362 | Dominant | 4.621 (3.517-6.072) | 0.0001 | - | - | - | - | BCC | Weak |
|  |  |  |  | 1 | 757/362 | Heterozygous | 4.772 (3.261-6.289) | 0.0001 | - | - | - | - | BCC | Weak |
|  |  |  |  | 1 | 757/362 | Allele | 3.209 (2.542-4.049) | 0.0001 | - | - | - | - | BCC | Weak |
|  | rs1801131 (C/T) | Poor | Asian | 1 | 191/563 | Heterozygous | 0.651 (0.448-0.946) | 0.0240 | - | - | - | - | BCC | Weak |
| *NBS1* | rs1805794 (G/C) | Good | Asian | 1 | 82/63 | Dominant | 4.056 (1.702-9.663) | 0.0020 | - | - | - | - | CCC | Weak |
| *OCT2* | rs1869641 (G/A) | Good | Asian | 1 | 149/184 | Dominant | 1.614 (0.999-2.608) | 0.0500 | - | - | - | - | BCC | Weak |
|  | rs316003 (C/T) | Good | Asian | 1 | 272/108 | Allele | 0.497 (0.307-0.807) | 0.0050 | - | - | - | - | BCC | Weak |
|  | rs316019 (T/C) | Good | Asian | 1 | 272/108 | Allele | 0.497 (0.307-0.807) | 0.0050 | - | - | - | - | BCC | Weak |
| *OPN* | rs11730582 (C/T) | Good | Asian | 1 | 196/180 | Dominant | 0.373 (0.235-0.590) | 0.0001 | - | - | - | - | BCC | Weak |
|  |  |  |  | 1 | 196/180 | Recessive | 0.344 (0.227-0.519) | 0.0001 | - | - | - | - | BCC | Weak |
|  |  |  |  | 1 | 196/180 | Homozygous | 0.226 (0.133-0.384) | 0.0001 | - | - | - | - | BCC | Weak |
|  |  |  |  | 1 | 196/180 | Heterozygous | 0.515 (0.314-0.846) | 0.0090 | - | - | - | - | BCC | Weak |
|  |  |  |  | 1 | 196/180 | Allele | 0.441 (0.337-0.577) | 0.0001 | - | - | - | - | BCC | Weak |
| *PD-L1* | rs2297136 (T/C) | Good | Asian | 1 | 196/180 | Dominant | 0.558 (0.357-0.973) | 0.0110 | - | - | - | - | BCC | Weak |
|  |  |  |  | 1 | 196/180 | Recessive | 0.361 (0.141-0.926) | 0.0340 | - | - | - | - | CCC | Weak |
|  |  |  |  | 1 | 196/180 | Heterozygous | 0.579 (0.367-0.914) | 0.0190 | - | - | - | - | BCC | Weak |
|  |  |  |  | 1 | 196/180 | Allele | 0.592 (0.398-0.880) | 0.0100 | - | - | - | - | BCC | Weak |
|  | rs4143815 (C/G) | Good | Asian | 1 | 192/174 | Recessive | 0.487 (0.277-0.857) | 0.0130 | - | - | - | - | CCC | Weak |
|  |  |  |  | 1 | 192/174 | Homozygous | 0.468 (0.252-0.871) | 0.0170 | - | - | - | - | CCC | Weak |
|  |  |  |  | 1 | 192/174 | Allele | 0.723 (0.537-0.972) | 0.0320 | - | - | - | - | BCC | Weak |
| *PFKL* | rs2073436 (C/G) | Good | Asian | 1 | 187/172 | Dominant | 1.575 (1.036-2.393) | 0.0330 | - | - | - | - | CCC | Weak |
|  |  |  |  | 1 | 187/172 | Recessive | 2.511 (1.022-6.169) | 0.0450 | - | - | - | - | CCC | Weak |
|  |  |  |  | 1 | 187/172 | Homozygous | 2.911 (1.163-7.285) | 0.0220 | - | - | - | - | CCC | Weak |
|  |  |  |  | 1 | 187/172 | Allele | 1.546 (1.104-2.165) | 0.0110 | - | - | - | - | BCC | Weak |
| *POLK* | rs3213801 (A/G) | Good | Asian | 1 | 538/120 | Dominant | 2.112 (1.150-3.878) | 0.0160 | - | - | - | - | CCC | Weak |
|  |  |  |  | 1 | 538/120 | Homozygous | 2.112 (1.150-3.878) | 0.0160 | - | - | - | - | CCC | Weak |
|  | rs5744533 (A/G) | Good | Asian | 1 | 538/120 | Dominant | 2.112 (1.150-3.878) | 0.0160 | - | - | - | - | CCC | Weak |
| *PTEN* | rs701848 (G/A) | Good | Asian | 1 | 111/100 | Dominant | 1.592 (1.027-2.468) | 0.0380 | - | - | - | - | BCC | Weak |
|  |  |  |  | 1 | 111/100 | Homozygous | 1.951 (1.030-3.696) | 0.0400 | - | - | - | - | CCC | Weak |
|  |  |  |  | 1 | 111/100 | Allele | 1.427 (1.047-1.946) | 0.0240 | - | - | - | - | BCC | Weak |
| *RAGE* | rs1800624 (T/A) | Good | Asian | 1 | 133/274 | Dominant | 1.679 (1.012-2.788) | 0.0450 | - | - | - | - | CCC | Weak |
|  | rs2070600 (G>T/A) | Good | Asian | 1 | 274/133 | Dominant | 2.432 (1.541-3.839) | 0.0001 | - | - | - | - | BCC | Weak |
|  |  |  |  | 1 | 274/133 | Homozygous | 2.213 (1.206-4.059) | 0.0100 | - | - | - | - | CCC | Weak |
|  |  |  |  | 1 | 274/133 | Heterozygous | 2.550 (1.544-4.212) | 0.0001 | - | - | - | - | BCC | Weak |
|  |  |  |  | 1 | 274/133 | Allele | 1.676 (1.218-2.307) | 0.0200 | - | - | - | - | BCC | Weak |
| *REV3* | rs240969 (G/A) | Good | Asian | 1 | 536/119 | Dominant | 1.715 (1.150-2.557) | 0.0080 | - | - | - | - | BCC | Weak |
|  |  |  |  | 1 | 536/119 | Heterozygous | 1.696 (1.108-2.594) | 0.0150 | - | - | - | - | BCC | Weak |
|  |  |  |  | 1 | 536/119 | Allele | 1.458 (1.073-1.980) | 0.0160 | - | - | - | - | BCC | Weak |
|  | rs456865 (G/A) | Good | Asian | 1 | 532/119 | Recessive | 1.573 (1.011-2.447) | 0.0440 | - | - | - | - | BCC | Weak |
|  |  |  |  | 1 | 532/119 | Allele | 1.350 (1.018-1.791) | 0.0370 | - | - | - | - | BCC | Weak |
| *REV7* | rs23336030 (G/A) | Good | Asian | 1 | 537/124 | Allele | 0.622 (0.467-0.828) | 0.0010 | - | - | - | - | BCC | Weak |
| *RRM1* | rs11030813 (C/T) | Good | Asian/European | 2 | 234/148 | Dominant | 0.432 (0.198-0.942) | 0.0350 | 0.0 | 0.494 | 0.317 | - | CAB | Weak |
|  |  |  |  | 2 | 234/148 | Heterozygous | 0.378 (0.168-0.853) | 0.0190 | 0.0 | 0.414 | 0.317 | - | CAB | Weak |
| *TMEM205* | rs896412 (G/C) | Good | Asian | 1 | 153/138 | Recessive | 10.667 (1.371-82.995) | 0.0240 | - | - | - | - | CCC | Weak |
|  |  |  |  | 1 | 153/138 | Homozygous | 10.768 (1.374-84.096) | 0.0240 | - | - | - | - | CCC | Weak |
| *TP53* | rs1042522 (A/C) | Good | Asian | 1 | 409/231 | Dominant | 0.528 (0.374-0.745) | 0.0001 | - | - | - | - | BCC | Weak |
|  |  |  |  | 1 | 409/231 | Recessive | 0.423 (0.264-0.677) | 0.0001 | - | - | - | - | CCC | Weak |
|  |  |  |  | 1 | 409/231 | Homozygous | 0.346 (0.206-0.580) | 0.0001 | - | - | - | - | CCC | Weak |
| *XPC* | rs77907221 (S/L) | Good | Asian | 2 | 226/125 | Recessive | 0.357 (0.195-0.654) | 0.0010 | 0.0 | 0.812 | 0.317 | - | CAB | Weak |
|  |  |  |  | 2 | 226/125 | Homozygous | 0.344 (0.178-0.666) | 0.0020 | 0.0 | 0.860 | 0.317 | - | CAB | Weak |
|  |  |  |  | 2 | 226/125 | Heterozygous | 0.369 (0.193-0.703) | 0.0020 | 0.0 | 0.791 | 0.317 | - | BAB | Moderate |
|  |  |  |  | 2 | 226/125 | Allele | 0.639 (0.465-0.877) | 0.0060 | 0.0 | 0.943 | 0.317 | - | BAB | Moderate |
| *XRCC1* | rs1799782 (C/T) | Good | Asian/European | 13 | 1593/1395 | Dominant | 0.598 (0.444-0.806) | 0.0010 | 63.5 | 0.001 | 0.010 | 0.004 | ABC | Weak |
|  |  | Good | Asian | 12 | 1521/1306 | Recessive | 0.611 (0.453-0.825) | 0.0010 | 33.6 | 0.122 | 0.217 | 0.199 | BAA | Moderate |
|  |  |  |  | 12 | 1521/1306 | Homozygous | 0.494 (0.316-0.773) | 0.0020 | 59.1 | 0.005 | 0.493 | 0.238 | BBA | Moderate |
|  |  |  |  | 12 | 1521/1306 | Heterozygous | 0.600 (0.429-0.838) | 0.0030 | 65.2 | 0.001 | 0.003 | 0.000 | BBC | Weak |
|  |  |  |  | 12 | 1521/1306 | Allele | 0.625 (0.473-0.825) | 0.0010 | 79.5 | 0.000 | 0.131 | 0.651 | ABA | Moderate |
|  | rs25487 (G/A) | Good | Asian | 19 | 2948/2035 | Recessive | 1.526 (1.105-2.107) | 0.0100 | 50.5 | 0.006 | 0.050 | 0.064 | ABB | Moderate |
|  |  |  |  | 19 | 2948/2035 | Homozygous | 0.647 (0.455-0.920) | 0.0150 | 55.0 | 0.020 | 0.142 | 0.078 | BBA | Moderate |
| *XRCC3* | rs861539 (C/T) | Good | Asian/European | 6 | 636/339 | Dominant | 0.686 (0.508-0.925) | 0.0130 | 0.0 | 0.964 | 0.348 | 0.270 | BAA | Moderate |
|  |  |  |  | 5 | 550/295 | Homozygous | 0.510 (0.310-0.838) | 0.0080 | 0.0 | 0.895 | 0.117 | 0.136 | CAA | Weak |
|  |  |  |  | 5 | 550/295 | Allele | 0.734 (0.586-0.918) | 0.0070 | 0.0 | 0.985 | 0.327 | 0.469 | BAA | Moderate |
