# Supplementary material for: Genetic Variants Associated With Response to Platinum-Based Chemotherapy in Non-Small Cell Lung Cancer Patients: A Field Synopsis and Meta‐Analysis
Source: Br J Biomed Sci. 2024 Feb 21;81:11835. doi: 10.3389/bjbs.2024.11835 (PMC10914946; doi:10.3389/bjbs.2024.11835)
Supplement: Supplementary file 3 [file Table3.docx]

**Supplementary Table 3** Genetic variants associated with PBC response in subgroup analysis based on ethnicity

| **Genes** | **Variants** | **Subgroup** | **Number evaluated** | | **Genetic associations with PBC response** | | | **Heterogeneity** | | **Begg P** | **Egger P** | **Venice criteria grades** | **Credibility of evidence** |
| --- | --- | --- | --- | --- | --- | --- | --- | --- | --- | --- | --- | --- | --- |
|  |  |  | **Studies** | **Cases/controls** | **Genetic models** | **OR (95% CI)** | **p-value** | **I2 (%)** | **P (Q test)** |  |  |  |  |
| *ERCC1* | rs11615 (C/T) | Asian | 14 | 1027/767 | Homozygous | 1.287 (0.729-2.272) | 0.385 | 59.5 | 0.0040 | 0.170 | 0.873 | BAA | Moderate |
|  |  |  | 14 | 1027/767 | Heterozygous | 1.165 (0.854-1.589) | 0.321 | 52.6 | 0.0110 | 0.622 | 0.976 | BAA | Moderate |
|  |  |  | 17 | 1175/824 | Dominant | 1.189 (0.843-1.676) | 0.324 | 66.2 | 0.0001 | 0.870 | 0.937 | ABA | Moderate |
|  |  |  | 14 | 1027/767 | Recessive | 1.287 (0.729-2.272) | 0.385 | 59.5 | 0.0040 | 0.170 | 0.873 | BAA | Moderate |
|  |  |  | 14 | 1027/767 | Allele | 1.112 (0.835-1.481) | 0.469 | 71.0 | 0.0001 | 0.956 | 0.815 | ABA | Moderate |
|  |  | European | 7 | 338/320 | Homozygous | 0.851 (0.510-1.418) | 0.535 | 0.0 | 0.7270 | 0.652 | 0.550 | BAA | Moderate |
|  |  |  | 7 | 338/320 | Heterozygous | 0.771 (0.496-1.197) | 0.246 | 0.0 | 0.5880 | 0.652 | 0.760 | BAA | Moderate |
|  |  |  | 8 | 410/409 | Dominant | 0.789 (0.520-1.197) | 0.265 | 0.0 | 0.7330 | 0.453 | 0.725 | BBA | Moderate |
|  |  |  | 9 | 484/394 | Recessive | 0.964 (0.659-1.410) | 0.849 | 0.0 | 0.4540 | 0.293 | 0.122 | BAA | Moderate |
|  |  |  | 7 | 338/320 | Allele | 0.923 (0.729-1.169) | 0.508 | 0.0 | 0.8390 | 0.051 | 0.434 | BCA | Weak |
|  | rs3212986 (C/T) | Asian | 11 | 1063/677 | Dominant | 1.556 (1.078-2.244) | 0.018 | 60.5 | 0.0070 | 0.139 | 0.186 | BAA | Moderate |
|  |  | European | 4 | 260/216 | Dominant | 0.718 (0.510-1.010) | 0.057 | 0.0 | 0.7650 | 0.497 | 0.258 | BAA | Moderate |
| *ERCC2* | rs13181 (A/C) | Asian | 7 | 456/304 | Allele | 1.061 (0.717-1.571) | 0.766 | 0.0 | 0.5970 | 0.851 | 0.877 | BAA | Moderate |
|  |  |  | 11 | 933/490 | Dominant | 1.012 (0.667-1.537) | 0.954 | 0.0 | 0.6330 | 0.851 | 0.765 | BAA | Moderate |
|  |  |  | 7 | 456/304 | Heterozygous | 0.966 (0.634-1.472) | 0.590 | 0.0 | 0.7000 | 0.851 | 0.726 | BAA | Moderate |
|  |  | European | 7 | 461/360 | Allele | 0.839 (0.502-1.404) | 0.505 | 82.0 | 0.0001 | 0.453 | 0.681 | BCA | Weak |
|  |  |  | 10 | 626/479 | Dominant | 0.744 (0.562-1.003) | 0.052 | 47.0 | 0.0790 | 0.099 | 0.301 | BBA | Moderate |
|  |  |  | 7 | 461/360 | Heterozygous | 0.781 (0.556-1.097) | 0.154 | 0.0 | 0.9660 | 0.293 | 0.584 | BAA | Moderate |
