# Supplementary material for: Genetic Variants Associated With Response to Platinum-Based Chemotherapy in Non-Small Cell Lung Cancer Patients: A Field Synopsis and Meta‐Analysis
Source: Br J Biomed Sci. 2024 Feb 21;81:11835. doi: 10.3389/bjbs.2024.11835 (PMC10914946; doi:10.3389/bjbs.2024.11835)
Supplement: Supplementary file 4 [file Table4.docx]

**Supplementary Table 4** Genetic variants not significantly associated with response to PBC

| **Genes** | **Variants** | **Study quality (NOS)** | **Ethnicity** | **Number evaluated** | | **Genetic associations with PBC response** | | | **Heterogeneity** | | **Begg P** | **Egger P** | **Venice criteria grades** | **Credibility of evidence** |
| --- | --- | --- | --- | --- | --- | --- | --- | --- | --- | --- | --- | --- | --- | --- |
|  |  |  |  | **Studies** | **Cases/ controls** | **Genetic model** | **OR (95% CI)** | **p-value** | **I2 (%)** | **P (Q test)** |  |  |  |  |
| *ABCB1* | rs1128503 (C/T) | Good/Poor | Asian | 2 | 261/79 | Recessive | 0.847 (0.476-0.505) | 0.570 | 0.0 | 0.9230 | 0.317 | - | BAB | Moderate |
|  |  |  |  | 2 | 261/79 | Allele | 0.782 (0.523-0.169) | 0.231 | 0.0 | 0.9280 | 0.317 | - | BAB | Moderate |
|  | rs2032582 (G>T/A) | Good/Poor | Asian | 4 | 166/143 | Allele | 1.365 (0.828-2.249) | 0.222 | 55.4 | 0.0810 | 0.497 | 0.060 | BAA | Moderate |
|  | rs1045642 (C/T) | Good/Poor | Asian/ European | 6 | 671/308 | Homozygous | 1.512 (0.957-2.389) | 0.077 | 0.0 | 0.5720 | 0.573 | 0.561 | BAA | Moderate |
|  |  |  |  | 6 | 671/308 | Heterozygous | 1.081 (0.790-1.480) | 0.626 | 0.0 | 0.8260 | 0.348 | 0.432 | BAA | Moderate |
|  |  |  |  | 9 | 824/382 | Dominant | 1.167 (0.772-1.776) | 0.464 | 55.0 | 0.0230 | 0.061 | 0.438 | BAA | Moderate |
|  |  |  |  | 6 | 671/308 | Recessive | 1.339 (0.884-2.027) | 0.168 | 0.0 | 0.6030 | 0.573 | 0.298 | BAA | Moderate |
|  |  |  |  | 6 | 671/308 | Allele | 1.085 (0.880-1.337) | 0.446 | 0.0 | 0.6220 | 0.348 | 0.356 | BAA | Moderate |
|  | rs1128503 (C/T) | Good/Poor | Asian | 2 | 261/79 | Homozygous | 0.413 (0.147-1.164) | 0.094 | 0.0 | 0.9990 | 0.317 | - | CAB | Weak |
|  |  |  |  | 2 | 261/79 | Heterozygous | 0.414 (0.154-1.111) | 0.080 | 0.0 | 0.9270 | 0.317 | - | CAB | Weak |
|  |  |  |  | 2 | 261/79 | Dominant | 0.413 (0.158-1.076) | 0.070 | 0.0 | 0.9550 | 0.317 | - | CAB | Weak |
|  | rs2032582 (G>T/A) | Good/Poor | Asian | 4 | 166/143 | Homozygous | 1.456 (0.687-3.088) | 0.327 | 22.1 | 0.2780 | 0.174 | 0.102 | CAA | Weak |
|  |  |  |  | 4 | 166/143 | Recessive | 0.990 (0.570-1.717) | 0.971 | 0.0 | 0.4980 | 0.174 | 0.283 | CAA | Weak |
|  | E1/-129(T/C) | Good | Asian | 1 | 50/46 | Heterozygous | 0.565 (0.149-2.148) | 0.402 | - | - | - | - | CCC | Weak |
|  |  |  |  | 1 | 50/46 | Dominant | 0.565 (0.149-2.148) | 0.402 | - | - | - | - | CCC | Weak |
|  |  |  |  | 1 | 50/46 | Allele | 0.583 (0.159-2.137) | 0.416 | - | - | - | - | CCC | Weak |
| *ABCC2* | rs2273697 (G/A) | Good/Poor | Asian | 3 | 418/192 | Heterozygous | 1.125 (0.663-1.907) | 0.662 | 18.7 | 0.2920 | 0.602 | 0.691 | BAA | Moderate |
|  |  |  |  | 3 | 418/192 | Allele | 1.000 (0.733-1.365) | 1.000 | 0.0 | 1.0000 | 1.000 | - | BAB | Moderate |
|  | rs717620 (C/T) | Good/Poor | Asian | 3 | 344/110 | Homozygous | 0.311 (0.053-1.526) | 0.150 | 41.9 | 0.1790 | 0.602 | 0.339 | CBA | Weak |
|  |  |  |  | 3 | 344/110 | Heterozygous | 0.644 (0.366-1.069) | 0.089 | 85.8 | 0.0010 | 0.117 | 0.460 | BCA | Weak |
|  |  |  |  | 4 | 680/186 | Dominant | 1.134 (0.276-4.659) | 0.861 | 93.0 | 0.0001 | 0.497 | 0.712 | BCA | Weak |
|  |  |  |  | 3 | 344/110 | Recessive | 0.682 (0.065-7.114) | 0.749 | 73.9 | 0.0220 | 0.602 | 0.252 | CBA | Weak |
|  | rs2273697 (G/A) | Good/Poor | Asian | 3 | 418/192 | Homozygous | 0.627 (0.135-2.920) | 0.552 | 0.0 | 0.9280 | 0.602 | 0.960 | CAA | Weak |
|  |  |  |  | 4 | 751/268 | Dominant | 0.841 (0.341-2.077) | 0.708 | 83.3 | 0.0001 | 0.174 | 0.775 | BCA | Weak |
|  |  |  |  | 3 | 418/192 | Recessive | 0.638 (0.138-2.959) | 0.566 | 0.0 | 0.9240 | 0.602 | 0.834 | CAA | Weak |
|  | rs3740066 (C/T) | Good/Poor | Asian | 4 | 615/217 | Homozygous | 0.834 (0.404-1.720) | 0.623 | 0.0 | 0.4970 | 0.317 | 0.254 | BAA | Moderate |
|  |  |  |  | 4 | 615/217 | Heterozygous | 0.854 (0.463-1.576) | 0.614 | 25.4 | 0.2590 | 0.174 | 0.240 | BAA | Moderate |
|  |  |  |  | 5 | 954/290 | Dominant | 1.051 (0.732-1.507) | 0.788 | 41.4 | 0.1460 | 0.001 | 0.730 | BBB | Moderate |
|  |  |  |  | 4 | 615/217 | Recessive | 0.934 (0.531-1.645) | 0.814 | 33.0 | 0.2140 | 0.497 | 0.135 | BBA | Moderate |
|  |  |  |  | 4 | 615/217 | Allele | 0.902 (0.596-1.364) | 0.624 | 52.0 | 1.0000 | 1.000 | 0.356 | BBA | Moderate |
| *ABCG2* | rs2231142 (C/A) | Good/Poor | Asian | 3 | 1059/256 | Heterozygous | 0.940 (0.695-1.271) | 0.687 | 0.0 | 0.5900 | 0.602 | 0.808 | AAB | Moderate |
|  |  |  |  | 3 | 1059/256 | Dominant | 0.762 (0.577-1.006) | 0.055 | 0.0 | 0.6800 | 0.602 | 0.204 | AAB | Moderate |
|  | rs2231137 (G/A) | Poor | Asian | 1 | 55/47 | Homozygous | 0.538 (0.083-3.471) | 0.514 | - | - | - | - | CCC | Weak |
|  |  |  |  | 1 | 55/47 | Heterozygous | 0.934 (0.416-2.097) | 0.868 | - | - | - | - | CCC | Weak |
|  |  |  |  | 1 | 55/47 | Dominant | 0.880 (0.402-1.924) | 0.748 | - | - | - | - | CCC | Weak |
|  |  |  |  | 1 | 55/47 | Recessive | 0.553 (0.088-3.462) | 0.527 | - | - | - | - | CCC | Weak |
|  |  |  |  | 1 | 55/47 | Allele | 0.865 (0.458-1.632) | 0.653 | - | - | - | - | CCC | Weak |
|  | rs2231164 (G/A) | Good | Asian | 1 | 798/177 | Recessive | 1.019 (0.709-1.467) | 0.917 | - | - | - | - | BCC | Weak |
|  |  |  |  | 1 | 798/177 | Homozygous | 1.432 (0.923-2.221) | 0.109 | - | - | - | - | BCC | Weak |
|  |  |  |  | 1 | 798/177 | Allele | 1.197 (0.950-1.507) | 0.127 | - | - | - | - | BCC | Weak |
|  | rs4148157 (G/A) | Good | Asian | 1 | 798/177 | Heterozygous | 0.867 (0.615-1.222) | 0.414 | - | - | - | - | BCC | Weak |
|  |  |  |  | 1 | 798/177 | Dominant | 0.789 (0.569-1.093) | 0.153 | - | - | - | - | BCC | Weak |
|  | rs1871744 (A/G) | Good | Asian | 1 | 798/177 | Homozygous | 1.168 (0.70-1.951) | 0.552 | - | - | - | - | BCC | Weak |
|  |  |  |  | 1 | 798/177 | Recessive | 0.952 (0.580-1.562) | 0.846 | - | - | - | - | BCC | Weak |
|  |  |  |  | 1 | 798/177 | Allele | 1.266 (0.980-1.635) | 0.071 | - | - | - | - | BCC | Weak |
| *AKT1* | rs2498786 (C/G) | Good | Asian | 1 | 152/40 | Homozygous | 2.557 (0.319-20.481) | 0.376 | - | - | - | - | BCC | Weak |
|  |  |  |  | 1 | 152/40 | Heterozygous | 3.421 (0.414-28.224) | 0.254 | - | - | - | - | BCC | Weak |
|  |  |  |  | 1 | 152/40 | Dominant | 2.832 (0.360-22.295) | 0.323 | - | - | - | - | BCC | Weak |
|  |  |  |  | 1 | 152/40 | Recessive | 0.861 (0.428-1.729) | 0.673 | - | - | - | - | BCC | Weak |
|  |  |  |  | 1 | 152/40 | Allele | 1.022 (0.567-1.840) | 0.942 | - | - | - | - | CCC | Weak |
|  | rs2494752 (A/G) | Good | Asian | 1 | 157/40 | Homozygous | 0.508 (0.105-2.446) | 0.398 | - | - | - | - | CCC | Weak |
|  |  |  |  | 1 | 157/40 | Heterozygous | 2.135 (0.996-4.557) | 0.051 | - | - | - | - | CCC | Weak |
|  |  |  |  | 1 | 157/40 | Dominant | 1.737 (0.823-3.665) | 0.147 | - | - | - | - | CCC | Weak |
|  |  |  |  | 1 | 157/40 | Recessive | 0.323 (0.073-1.435) | 0.138 | - | - | - | - | CCC | Weak |
|  |  |  |  | 1 | 157/40 | Allele | 1.082 (0.651-1.799) | 0.761 | - | - | - | - | BCC | Weak |
|  | rs2494750 (G/C) | Good | Asian | 1 | 155/40 | Homozygous | 0.950 (0.102-8.816) | 0.964 | - | - | - | - | CCC | Weak |
|  |  |  |  | 1 | 155/40 | Heterozygous | 0.924 (0.402-2.124) | 0.853 | - | - | - | - | CCC | Weak |
|  |  |  |  | 1 | 155/40 | Dominant | 0.927 (0.417-2.062) | 0.852 | - | - | - | - | CCC | Weak |
|  |  |  |  | 1 | 155/40 | Recessive | 0.968 (0.105-8.907) | 0.977 | - | - | - | - | CCC | Weak |
|  |  |  |  | 1 | 155/40 | Allele | 0.939 (0.461-1.910) | 0.862 | - | - | - | - | CCC | Weak |
|  | rs74090038 (C/T) | Good | Asian | 1 | 158/39 | Homozygous | 0.593 (0.124-2.824) | 0.511 | - | - | - | - | CCC | Weak |
|  |  |  |  | 1 | 158/39 | Heterozygous | 1.956 (0.936-4.085) | 0.074 | - | - | - | - | CCC | Weak |
|  |  |  |  | 1 | 158/39 | Dominant | 1.641 (0.802-3.360) | 0.175 | - | - | - | - | CCC | Weak |
|  |  |  |  | 1 | 158/39 | Recessive | 0.420 (0.093-1.894) | 0.259 | - | - | - | - | CCC | Weak |
|  |  |  |  | 1 | 158/39 | Allele | 1.146 (0.676-1.943) | 0.614 | - | - | - | - | CCC | Weak |
|  | rs34716810 (C/T) | Good | Asian | 1 | 156/40 | Homozygous | 0.809 (0.081-8.047) | 0.856 | - | - | - | - | CCC | Weak |
|  |  |  |  | 1 | 156/40 | Heterozygous | 0.632 (0.058-6.880) | 0.706 | - | - | - | - | CCC | Weak |
|  |  |  |  | 1 | 156/40 | Dominant | 2.854 (0.285-28.612) | 0.373 | - | - | - | - | CCC | Weak |
|  |  |  |  | 1 | 156/40 | Allele | 1.149 (0.551-2.397) | 0.711 | - | - | - | - | CCC | Weak |
| *AKT2* | rs62107593 (C/G) | Good | Asian | 1 | 153/41 | Homozygous | 0.612 (0.186-2.017) | 0.420 | - | - | - | - | CCC | Weak |
|  |  |  |  | 1 | 153/41 | Heterozygous | 1.421 (0.676-2.988) | 0.354 | - | - | - | - | CCC | Weak |
|  |  |  |  | 1 | 153/41 | Dominant | 1.181 (0.579-2.409) | 0.647 | - | - | - | - | CCC | Weak |
|  |  |  |  | 1 | 153/41 | Recessive | 0.691 (0.223-2.139) | 0.522 | - | - | - | - | CCC | Weak |
|  |  |  |  | 1 | 153/41 | Allele | 0.919 (0.555-1.523) | 0.744 | - | - | - | - | CCC | Weak |
| *APA1* | rs7975232 (G/T) | Good | Asian | 1 | 434/321 | Heterozygous | 1.365 (0.982-1.896) | 0.064 | - | - | - | - | BCC | Weak |
| *APE1* | rs1130409 (T/G) | Good | Asian | 3 | 296/162 | Heterozygous | 0.995 (0.645-1.533) | 0.981 | 0.0 | 0.9270 | 0.117 | 0.132 | BAA | Moderate |
|  |  |  |  | 3 | 296/162 | Dominant | 1.146 (0.762-1.723) | 0.513 | 0.0 | 0.9100 | 0.117 | 0.191 | BAA | Moderate |
|  |  |  |  | 3 | 296/162 | Allele | 1.240 (0.940-1.634) | 0.128 | 0.0 | 0.9000 | 0.117 | 0.188 | BAA | Moderate |
|  |  |  |  | 3 | 296/162 | Homozygous | 1.612 (0.908-2.861) | 0.103 | 0.0 | 0.8650 | 0.117 | 0.126 | CAA | Weak |
|  |  |  |  | 3 | 296/162 | Recessive | 1.626 (0.977-2.705) | 0.061 | 0.0 | 0.9090 | 0.117 | 0.148 | CAA | Weak |
|  | T141G | Good | Asian | 1 | 151/84 | Homozygous | 0.676 (0.323-1.416) | 0.300 | - | - | - | - | BCC | Weak |
|  |  |  |  | 1 | 151/84 | Heterozygous | 0.709 (0.381-1.319) | 0.278 | - | - | - | - | BCC | Weak |
|  |  |  |  | 1 | 151/84 | Dominant | 0.698 (0.392-1.244) | 0.223 | - | - | - | - | BCC | Weak |
|  |  |  |  | 1 | 151/84 | Recessive | 0.827 (0.436-1.566) | 0.559 | - | - | - | - | BCC | Weak |
|  |  |  |  | 1 | 151/84 | Allele | 0.798 (0.546-1.167) | 0.244 | - | - | - | - | BCC | Weak |
| *APQ2* | rs7314734 (C/T) | Good | Asian | 1 | 154/184 | Homozygous | 1.574 (0.141-17.565) | 0.712 | - | - | - | - | CCC | Weak |
|  |  |  |  | 1 | 154/184 | Recessive | 0.718 (0.065-7.978) | 0.788 | - | - | - | - | CCC | Weak |
| *APQ9* | rs1516400 (T/C) | Good | Asian | 1 | 150/182 | Homozygous | 1.423 (0.776-2.610) | 0.254 | - | - | - | - | CCC | Weak |
|  |  |  |  | 1 | 150/182 | Heterozygous | 1.106 (0.665-1.840) | 0.698 | - | - | - | - | CCC | Weak |
|  |  |  |  | 1 | 150/182 | Dominant | 1.210 (0.751-1.949) | 0.434 | - | - | - | - | CCC | Weak |
|  |  |  |  | 1 | 150/182 | Recessive | 1.336 (0.798-2.239) | 0.271 | - | - | - | - | CCC | Weak |
|  |  |  |  | 1 | 150/182 | Allele | 1.196 (0.880-1.625) | 0.253 | - | - | - | - | BCC | Weak |
| *ATM* | rs664143 (A/G) | Good | Asian | 1 | 50/26 | Homozygous | 1.604 (0.339-7.597) | 0.551 | - | - | - | - | CCC | Weak |
|  |  |  |  | 1 | 50/26 | Heterozygous | 0.629 (0.211-1.877) | 0.406 | - | - | - | - | CCC | Weak |
|  |  |  |  | 1 | 50/26 | Dominant | 0.783 (0.274-2.239) | 0.648 | - | - | - | - | CCC | Weak |
|  |  |  |  | 1 | 50/26 | Recessive | 2.162 (0.546-8.567) | 0.272 | - | - | - | - | CCC | Weak |
|  |  |  |  | 1 | 50/26 | Allele | 1.116 (0.567-2.195) | 0.751 | - | - | - | - | CCC | Weak |
| *ATP7A* | rs2227291 (C/G) | Good | Asian | 1 | 69/28 | Recessive | 1.270 (0.502-3.211) | 0.614 | - | - | - | - | CCC | Weak |
| *ATP7B* | rs9526814 (T/G) | Good | Asian | 1 | 86/62 | Homozygous | 0.429 (0.162-1.134) | 0.088 | - | - | - | - | CCC | Weak |
|  |  |  |  | 1 | 86/62 | Heterozygous | 1.152 (0.559-2.372) | 0.702 | - | - | - | - | CCC | Weak |
|  |  |  |  | 1 | 86/62 | Dominant | 0.870 (0.450-1.683) | 0.680 | - | - | - | - | CCC | Weak |
|  |  |  |  | 1 | 86/62 | Allele | 0.714 (0.442-1.155) | 0.170 | - | - | - | - | CCC | Weak |
|  | rs1061472 (A/G) | Good | Asian | 1 | 209/204 | Homozygous | 1.324 (0.718-2.440) | 0.369 | - | - | - | - | CCC | Weak |
|  |  |  |  | 1 | 209/204 | Heterozygous | 1.377 (0.903-2.102) | 0.138 | - | - | - | - | BCC | Weak |
|  |  |  |  | 1 | 209/204 | Dominant | 1.365 (0.913-2.043) | 0.130 | - | - | - | - | BCC | Weak |
|  |  |  |  | 1 | 209/204 | Recessive | 1.099 (0.628-1.923) | 0.742 | - | - | - | - | CCC | Weak |
|  |  |  |  | 1 | 209/204 | Allele | 1.191 (0.900-1.576) | 0.222 | - | - | - | - | BCC | Weak |
|  | rs9535826 (T/G) | Good | Asian | 1 | 209/201 | Homozygous | 1.214 (0.681-2.164) | 0.510 | - | - | - | - | CCC | Weak |
|  |  |  |  | 1 | 209/201 | Heterozygous | 1.201 (0.746-1.935) | 0.451 | - | - | - | - | CCC | Weak |
|  |  |  |  | 1 | 209/201 | Dominant | 1.205 (0.764-1.901) | 0.423 | - | - | - | - | CCC | Weak |
|  |  |  |  | 1 | 209/201 | Recessive | 1.068 (0.666-1.713) | 0.784 | - | - | - | - | CCC | Weak |
|  |  |  |  | 1 | 209/201 | Allele | 1.094 (0.832-1.438) | 0.521 | - | - | - | - | BCC | Weak |
| *AURORA* | rs2273535 (T/A) | Good | European | 1 | 102/50 | Homozygous | 0.525 (0.032-8.667) | 0.652 | - | - | - | - | CCC | Weak |
|  |  |  |  | 1 | 102/50 | Heterozygous | 1.234 (0.606-2.512) | 0.561 | - | - | - | - | CCC | Weak |
|  |  |  |  | 1 | 102/50 | Dominant | 1.195 (0.593-2.406) | 0.618 | - | - | - | - | CCC | Weak |
|  |  |  |  | 1 | 102/50 | Recessive | 0.485 (0.030-7.920) | 0.612 | - | - | - | - | CCC | Weak |
|  |  |  |  | 1 | 102/50 | Allele | 1.105 (0.604-2.022) | 0.745 | - | - | - | - | CCC | Weak |
|  | rs1047972 (G/A) | Good | European | 1 | 120/50 | Homozygous | 1.352 (0.135-13.504) | 0.797 | - | - | - | - | CCC | Weak |
|  |  |  |  | 1 | 120/50 | Heterozygous | 1.091 (0.554-2.150) | 0.801 | - | - | - | - | CCC | Weak |
|  |  |  |  | 1 | 120/50 | Dominant | 1.104 (0.567-2.151) | 0.771 | - | - | - | - | CCC | Weak |
|  |  |  |  | 1 | 120/50 | Recessive | 1.308 (0.133-12.874) | 0.818 | - | - | - | - | CCC | Weak |
|  |  |  |  | 1 | 120/50 | Allele | 1.093 (0.619-1.931) | 0.759 | - | - | - | - | CCC | Weak |
| *BAX* | rs4645878 (G/A) | Good | Asian | 1 | 151/84 | Homozygous | 0.548 (0.253-1.187) | 0.127 | - | - | - | - | CCC | Weak |
|  |  |  |  | 1 | 151/84 | Dominant | 1.331 (0.779-2.219) | 0.273 | - | - | - | - | BCC | Weak |
|  |  |  |  | 1 | 151/84 | Allele | 0.932 (0.632-1.375) | 0.723 | - | - | - | - | BCC | Weak |
| *BCL2* | rs2279115 (C/A) | Good | Asian | 1 | 328/263 | Heterozygous | 1.057 (0.743-1.504) | 0.758 | 0.0 | 0.9530 | 0.317 | - | BAB | Moderate |
|  |  |  |  | 1 | 328/263 | Dominant | 1.103 (0.793-1.535) | 0.561 | 0.0 | 0.9160 | 0.317 | - | BAB | Moderate |
|  |  |  |  | 1 | 328/263 | Allele | 1.111 (0.875-1.412) | 0.388 | 0.0 | 0.7660 | 0.317 | - | BAB | Moderate |
|  |  |  |  | 1 | 328/263 | Homozygous | 1.251 (0.760-2.059) | 0.378 | 0.0 | 0.7410 | 0.317 | - | CAB | Weak |
|  |  |  |  | 1 | 328/263 | Recessive | 1.140 (0.718-1.810) | 0.579 | 0.0 | 0.5350 | 0.317 | - | CAB | Weak |
| *BICR5* | rs9904341 (G/C) | Good | Asian | 1 | 182/173 | Homozygous | 0.716 (0.400-1.282) | 0.261 | - | - | - | - | CCC | Weak |
|  |  |  |  | 1 | 182/173 | Heterozygous | 1.094 (0.669-1.789) | 0.719 | - | - | - | - | CCC | Weak |
|  |  |  |  | 1 | 182/173 | Dominant | 0.959 (0.602-1.527) | 0.860 | - | - | - | - | CCC | Weak |
|  |  |  |  | 1 | 182/173 | Recessive | 0.674 (0.417-1.089) | 0.107 | - | - | - | - | CCC | Weak |
|  |  |  |  | 1 | 182/173 | Allele | 0.855 (0.643-1.138) | 0.283 | - | - | - | - | BCC | Weak |
| *BMP4* | G5826A | Good | Asian | 1 | 574/364 | Homozygous | 0.804 (0.554-1.167) | 0.251 | - | - | - | - | BCC | Weak |
|  |  |  |  | 1 | 574/364 | Heterozygous | 1.007 (0.745-1.363) | 0.962 | - | - | - | - | BCC | Weak |
|  |  |  |  | 1 | 574/364 | Dominant | 0.942 (0.710-1.251) | 0.680 | - | - | - | - | BCC | Weak |
|  |  |  |  | 1 | 574/364 | Recessive | 0.801 (0.579-1.107) | 0.179 | - | - | - | - | BCC | Weak |
|  |  |  |  | 1 | 574/364 | Allele | 0.906 (0.751-1.092) | 0.298 | - | - | - | - | BCC | Weak |
| *BRCA1* | rs799917 (C/T) | Good | Asian | 1 | 192/179 | Homozygous | 0.680 (0.323-1.435) | 0.312 | - | - | - | - | CCC | Weak |
|  |  |  |  | 1 | 192/179 | Recessive | 0.866 (0.424-1.771) | 0.694 | - | - | - | - | CCC | Weak |
|  |  |  |  | 1 | 192/179 | Allele | 0.738 (0.540-1.008) | 0.056 | - | - | - | - | CCC | Weak |
|  | rs179966 (A/G) | Good | Asian | 1 | 41/83 | Homozygous | 0.621 (0.161-2.402) | 0.490 | - | - | - | - | CCC | Weak |
|  |  |  |  | 1 | 41/83 | Recessive | 1.174 (0.323-4.263) | 0.808 | - | - | - | - | CCC | Weak |
|  |  |  |  | 1 | 41/83 | Allele | 0.570 (0.319-1.017) | 0.057 | - | - | - | - | CCC | Weak |
| *BSM1* | rs1544410 (A/G) | Good | Asian | 1 | 434/321 | Homozygous | 1.318 (0.882-1.970) | 0.178 | - | - | - | - | BCC | Weak |
|  |  |  |  | 1 | 434/321 | Heterozygous | 1.120 (0.805-1.558) | 0.503 | - | - | - | - | BCC | Weak |
|  |  |  |  | 1 | 434/321 | Dominant | 1.180 (0.867-1.605) | 0.292 | - | - | - | - | BCC | Weak |
|  |  |  |  | 1 | 434/321 | Recessive | 1.234 (0.867-1.755) | 0.243 | - | - | - | - | BCC | Weak |
|  |  |  |  | 1 | 434/321 | Allele | 1.157 (0.942-1.420) | 0.165 | - | - | - | - | BCC | Weak |
| *CASC8* | rs10505477 (A/G) | Good | Asian | 1 | 274/187 | Homozygous | 1.254 (0.731-2.149) | 0.411 | - | - | - | - | CCC | Weak |
|  |  |  |  | 1 | 274/187 | Recessive | 0.913 (0.567-1.472) | 0.710 | - | - | - | - | CCC | Weak |
|  |  |  |  | 1 | 274/187 | Allele | 1.185 (0.908-1.547) | 0.210 | - | - | - | - | BCC | Weak |
|  | rs3769818 (G/A) | Good | Asian | 1 | 190/168 | Dominant | 0.423 (0.060-2.995) | 0.389 | 71.3 | 0.0620 | 0.317 | - | BBB | Moderate |
|  |  |  | Asian/ | 2 | 199/233 | Allele | 0.666 (0.206-2.158) | 0.498 | 61.7 | 0.1060 | 0.317 | - | BBB | Moderate |
|  |  |  | European | 2 | 199/233 | Homozygous | 1.267 (0.594-2.699) | 0.540 | 0.0 | 0.5340 | 0.317 | - | CAB | Weak |
|  |  |  |  | 2 | 199/233 | Heterozygous | 0.831 (0.537-1.287) | 0.408 | - | - | - | - | BCC | Weak |
|  |  |  |  | 2 | 199/233 | Recessive | 1.463 (0.700-3.057) | 0.312 | 0.0 | 0.8520 | 0.317 | - | CAB | Weak |
| *CCAT2* | rs6983267 (G/T) | Good | Asian | 1 | 179/268 | Homozygous | 1.446 (0.836-2.502) | 0.187 | - | - | - | - | CCC | Weak |
|  |  |  |  | 1 | 179/268 | Heterozygous | 0.859 (0.513-1.437) | 0.562 | - | - | - | - | CCC | Weak |
|  |  |  |  | 1 | 179/268 | Dominant | 1.055 (0.650-1.712) | 0.827 | - | - | - | - | CCC | Weak |
|  |  |  |  | 1 | 179/268 | Allele | 1.257 (0.959-1.647) | 0.097 | - | - | - | - | BCC | Weak |
| *CCND1* | rs9344 (A/G) | Poor | European | 1 | 110/34 | Heterozygous | 1.553 (0.620-3.890) | 0.348 | - | - | - | - | CCC | Weak |
|  |  |  |  | 1 | 110/34 | Dominant | 1.996 (0.821-4.850) | 0.127 | - | - | - | - | CCC | Weak |
| *CDA* | rs1048977 (C/T) | Good | Asian/ | 2 | 199/69 | Heterozygous | 0.754 (0.405-1.401) | 0.371 | 24.2 | 0.2510 | 0.317 | - | BAB | Moderate |
|  |  |  | European | 2 | 199/69 | Dominant | 0.570 (0.322-1.007) | 0.053 | 31.6 | 0.2270 | 0.317 | - | BBB | Moderate |
|  | rs2072671 (A/C) | Good | Asian/ | 4 | 276/167 | Homozygous | 2.997 (0.469-19.138) | 0.246 | 79.8 | 0.0700 | 0.602 | 0.426 | CCA | Weak |
|  |  |  | European | 4 | 276/167 | Heterozygous | 1.587 (0.746-3.375) | 0.230 | 62.3 | 0.0470 | 1.000 | 0.955 | BCA | Weak |
|  |  |  |  | 4 | 276/167 | Dominant | 1.709 (0.699-4.179) | 0.240 | 75.9 | 0.0060 | 1.000 | 0.940 | BCA | Weak |
|  |  |  |  | 4 | 276/167 | Recessive | 2.203 (0.616-7.882) | 0.225 | 69.9 | 0.0190 | 0.497 | 0.159 | CCA | Weak |
|  |  |  |  | 4 | 276/167 | Allele | 1.560 (0.661-3.682) | 0.310 | 83.9 | 0.0001 | 1.000 | 0.811 | BCA | Weak |
|  | rs60369023 (G/A) | Good | Asian | 1 | 68/52 | Heterozygous | 1.241 (0.445-3.458) | 0.680 | - | - | - | - | CCC | Weak |
|  |  |  |  | 1 | 68/52 | Dominant | 1.241 (0.445-3.458) | 0.680 | - | - | - | - | CCC | Weak |
|  |  |  |  | 1 | 68/52 | Allele | 1.219 (0.456-3.262) | 0.693 | - | - | - | - | CCC | Weak |
|  | rs1048977 (C/T) | Good | Asian/European | 2 | 199/69 | Homozygous | 0.137 (0.016-1.155) | 0.068 | 64.4 | 0.0940 | 0.317 | - | CBB | Weak |
| *CDC25* | rs1380053 (A/C) | Good | Asian | 1 | 437/79 | Homozygous | 1.455 (0.179-11.842) | 0.726 | - | - | - | - | CCC | Weak |
|  |  |  |  | 1 | 437/79 | Heterozygous | 1 (0.564-1.773) | 1.000 | - | - | - | - | BCC | Weak |
|  |  |  |  | 1 | 437/79 | Dominant | 1.024 (0.585-1.793) | 0.934 | - | - | - | - | BCC | Weak |
|  |  |  |  | 1 | 437/79 | Recessive | 1.455 (0.179-11.793) | 0.726 | - | - | - | - | CCC | Weak |
|  |  |  |  | 1 | 437/79 | Allele | 1.045 (0.629-1.738) | 0.864 | - | - | - | - | BCC | Weak |
|  | rs3731513 (A/C) | Good | Asian | 1 | 437/79 | Heterozygous | 0.801 (0.459-1.397) | 0.434 | - | - | - | - | BCC | Weak |
|  |  |  |  | 1 | 437/79 | Dominant | 0.853 (0.490-1.485) | 0.574 | - | - | - | - | BCC | Weak |
|  |  |  |  | 1 | 437/79 | Allele | 0.932 (0.559-1.555) | 0.787 | - | - | - | - | BCC | Weak |
| *COX-2* | rs689465 (A/G) | Good | Asian | 1 | 126/64 | Dominant | 1.820 (0.722-4.289) | 0.171 | - | - | - | - | CCC | Weak |
| *COX-2* | rs689466 (A/G) | Good | Asian | 1 | 42/19 | Homozygous | 2.357 (0.640-8.677) | 0.197 | - | - | - | - | CCC | Weak |
|  |  |  |  | 1 | 42/19 | Heterozygous | 2.727 (0.856-8.685) | 0.090 | - | - | - | - | CCC | Weak |
|  |  |  |  | 2 | 168/83 | Dominant | 1.422 (0.538-3.756) | 0.478 | 60.2 | 0.1130 | 0.317 | - | CCB | Weak |
|  |  |  |  | 1 | 42/19 | Recessive | 1.291 (0.431-3.646) | 0.648 | - | - | - | - | CCC | Weak |
|  |  |  |  | 1 | 42/19 | Allele | 1.633 (0.828-3.222) | 0.157 | - | - | - | - | CCC | Weak |
|  | rs20417 (G/C) | Good | Asian | 1 | 126/64 | Allele | 0.443 (0.196-1.004) | 0.051 | - | - | - | - | CCC | Weak |
|  | rs3218625 (G/A) | Good | Asian | 1 | 126/64 | Heterozygous | 1.016 (1.090-11.422) | 0.990 | - | - | - | - | CCC | Weak |
|  |  |  |  | 1 | 126/64 | Dominant | 1.016 (1.090-11.422) | 0.990 | - | - | - | - | CCC | Weak |
|  |  |  |  | 1 | 126/64 | Allele | 1.016 (0.091-11.311) | 0.990 | - | - | - | - | CCC | Weak |
| *CTR1* | rs7851395 (A/G) | Good | Asian | 1 | 142/140 | Homozygous | 1.870 (0.921-3.797) | 0.083 | - | - | - | - | CCC | Weak |
|  |  |  |  | 1 | 142/140 | Heterozygous | 1.084 (0.637-1.845) | 0.765 | - | - | - | - | CCC | Weak |
|  |  |  |  | 1 | 142/140 | Dominant | 1.245 (0.752-2.062) | 0.395 | - | - | - | - | CCC | Weak |
|  |  |  |  | 1 | 142/140 | Recessive | 1.779 (0.951-3.328) | 0.072 | - | - | - | - | CCC | Weak |
|  |  |  |  | 1 | 142/140 | Allele | 1.303 (0.933-1.819) | 0.121 | - | - | - | - | BCC | Weak |
|  | rs12687377 (G/T) | Good | Asian | 1 | 142/140 | Homozygous | 1.853 (0.921-3.724) | 0.084 | - | - | - | - | CCC | Weak |
|  |  |  |  | 1 | 142/140 | Heterozygous | 0.730 (0.420-1.268) | 0.264 | - | - | - | - | CCC | Weak |
|  |  |  |  | 1 | 142/140 | Dominant | 0.950 (0.564-1.601) | 0.847 | - | - | - | - | CCC | Weak |
|  |  |  |  | 1 | 142/140 | Allele | 1.277 (0.917-1.779) | 0.148 | - | - | - | - | BCC | Weak |
| *CYP1A1* | rs1048943 (T/C) | Good | Asian | 2 | 110/162 | Recessive | 1.686 (0.896-3.172) | 0.105 | 0.0 | 0.8360 | 0.317 | - | CAB | Weak |
| *CYP2D6* | rs1065852 (T/C) | Good | Asian | 2 | 110/162 | Dominant | 1.069 (0.641-1.783) | 0.799 | 0.0 | 0.3470 | 0.317 | - | BAB | Moderate |
|  |  |  |  | 2 | 110/162 | Allele | 1.123 (0.784-1.608) | 0.526 | 26.8 | 0.2420 | 0.317 | - | BAB | Moderate |
|  |  |  |  | 2 | 110/162 | Homozygous | 1.071 (0.597-1.923) | 0.818 | 39.6 | 0.1980 | 0.317 | - | CAB | Weak |
|  |  |  |  | 2 | 110/162 | Heterozygous | 1.066 (0.583-1.948) | 0.836 | 0.0 | 0.7220 | 0.317 | - | CAB | Weak |
|  |  |  |  | 2 | 110/162 | Recessive | 1.043 (0.626-1.738) | 0.872 | 42.0 | 0.1890 | 0.317 | - | CAB | Weak |
| *CYP2E1* | rs3813867 (G/A) rs2031920 (C/T) | Good | Asian | 2 | 110/162 | Heterozygous | 1.052 (0.615-1.798) | 0.853 | 0.0 | 0.8810 | 0.317 | - | BAB | Moderate |
|  |  |  |  | 2 | 110/162 | Dominant | 0.967 (0.594-1.571) | 0.891 | 0.0 | 0.9870 | 0.317 | - | BAB | Moderate |
|  |  |  |  | 2 | 110/162 | Allele | 0.912 (0.626-1.328) | 0.630 | 0.0 | 0.8310 | 0.317 | - | BAB | Moderate |
|  |  |  |  | 2 | 110/162 | Homozygous | 0.785 (0.370-1.667) | 0.528 | 0.0 | 0.7150 | 0.317 | - | CAB | Weak |
|  |  |  |  | 2 | 110/162 | Recessive | 0.772 (0.374-1.595) | 0.484 | 0.0 | 0.6740 | 0.317 | - | CAB | Weak |
| *CYP3A55*3* | rs776746 (A/G) | Good | Asian | 1 | 39/30 | Recessive | 0.585 (0.211-1.624) | 0.303 | - | - | - | - | CCC | Weak |
| *EPO* | rs1617640 (G/T) | Good | Asian | 1 | 97/174 | Homozygous | 0.334 (0.093-1.198) | 0.092 | - | - | - | - | CCC | Weak |
|  |  |  |  | 1 | 97/174 | Heterozygous | 0.622 (0.167-2.314) | 0.479 | - | - | - | - | CCC | Weak |
|  |  |  |  | 1 | 97/174 | Dominant | 0.417 (0.118-1.476) | 0.175 | - | - | - | - | CCC | Weak |
| *ERCC1* | rs11615 (C/T) | Good | Asian/ | 21 | 1376/1087 | Homozygous | 1.135 (0.857-1.502) | 0.378 | 44.6 | 0.0190 | 0.807 | 0.836 | BBA | Moderate |
|  |  |  | European | 21 | 1376/1087 | Heterozygous | 1.079 (0.894-1.302) | 0.428 | 42.6 | 0.0210 | 0.305 | 0.441 | ABA | Moderate |
|  |  |  |  | 25 | 1585/1233 | Dominant | 1.106 (0.813-1.417) | 0.618 | 55.6 | 0.0010 | 0.398 | 0.429 | ABA | Moderate |
|  |  |  |  | 23 | 1511/1161 | Recessive | 1.088 (0.852-1.389) | 0.500 | 37.5 | 0.0510 | 0.861 | 0.769 | BAA | Moderate |
|  |  |  |  | 21 | 1376/1087 | Allele | 1.053 (0.858-1.291) | 0.623 | 59.3 | 0.0001 | 0.717 | 0.801 | ABA | Moderate |
| *ERCC1* | rs3212986 (C/A) | Good | Asian/European | 10 | 836/605 | Heterozygous | 1.329 (0.918-1.924) | 0.132 | 55.5 | 0.0160 | 0.089 | 0.058 | BBA | Moderate |
|  |  |  |  | 14 | 1251/1181 | Dominant | 1.227 (0.895-1.482) | 0.204 | 62.8 | 0.0010 | 0.171 | 0.250 | ABA | Moderate |
|  |  |  |  | 2 | 162/184 | Heterozygous | 0.980 (0.592-1.622) | 0.937 | 0.0 | 0.8540 | 0.317 | - | BAB | Moderate |
|  |  |  |  | 2 | 162/184 | Dominant | 0.924 (0.578-1.477) | 0.742 | 0.0 | 0.5910 | 0.317 | - | BAB | Moderate |
|  |  |  |  | 2 | 162/184 | Allele | 0.888 (0.649-1.216) | 0.459 | 0.0 | 0.3990 | 0.317 | - | BAB | Moderate |
|  |  |  |  | 2 | 162/184 | Homozygous | 0.809 (0.428-1.530) | 0.515 | 0.0 | 0.5070 | 0.317 | - | CAB | Weak |
|  |  |  |  | 2 | 162/184 | Recessive | 0.802 (0.481-1.339) | 0.399 | 0.0 | 0.5190 | 0.317 | - | CAB | Weak |
|  | rs2298881 (C/A) | Good | Asian | 4 | 404/378 | Homozygous | 1.236 (0.500-3.055) | 0.646 | 70.2 | 0.0180 | 0.497 | 0.933 | CBA | Weak |
|  |  |  |  | 4 | 404/378 | Heterozygous | 0.805 (0.488-1.327) | 0.395 | 0.0 | 0.6550 | 1.000 | 0.166 | CAA | Weak |
|  |  |  |  | 4 | 404/378 | Dominant | 0.995 (0.632-0.566) | 0.983 | 1.6 | 0.3840 | 0.174 | 0.226 | CAA | Weak |
|  |  |  |  | 4 | 404/378 | Recessive | 1.497 (0.636-3.400) | 0.367 | 85.7 | 0.0001 | 0.497 | 0.205 | BCA | Weak |
|  |  |  |  | 4 | 404/378 | Allele | 1.182 (0.683-2.045) | 0.551 | 81.9 | 0.0010 | 1.000 | 0.991 | BCA | Weak |
|  | rs226466 (T/A) | Good | Asian | 1 | 90/97 | Homozygous | 0.542 (0.250-1.172) | 0.120 | - | - | - | - | CCC | Weak |
|  |  |  |  | 1 | 90/97 | Heterozygous | 0.901 (0.429-1.894) | 0.783 | - | - | - | - | CCC | Weak |
|  |  |  |  | 1 | 90/97 | Dominant | 0.714 (0.362-1.408) | 0.331 | - | - | - | - | CCC | Weak |
|  |  |  |  | 1 | 90/97 | Recessive | 0.579 (0.315-1.065) | 0.079 | - | - | - | - | CCC | Weak |
|  |  |  |  | 1 | 90/97 | Allele | 0.688 (0.475-1.037) | 0.074 | - | - | - | - | CCC | Weak |
|  | rs6498486 (C/A) | Good | Asian | 1 | 90/97 | Homozygous | 0.748 (0.312-1.172) | 0.515 | - | - | - | - | CCC | Weak |
|  | rs6498486 (C/A) | Good | Asian | 1 | 90/97 | Heterozygous | 1.026 (0.400-2.629) | 0.958 | - | - | - | - | CCC | Weak |
|  |  |  |  | 1 | 90/97 | Dominant | 0.836 (0.360-1.943) | 0.677 | - | - | - | - | CCC | Weak |
|  |  |  |  | 1 | 90/97 | Recessive | 0.735 (0.412-1.312) | 0.298 | - | - | - | - | CCC | Weak |
|  |  |  |  | 1 | 90/97 | Allele | 0.790 (0.504-1.237) | 0.303 | - | - | - | - | CCC | Weak |
| *ERCC2 (XPD)* | rs13181 (A/C) | Good | European | 7 | 461/360 | Homozygous | 0.783 (0.326-1.882) | 0.585 | 71.3 | 0.0020 | 0.652 | 0.576 | BBA | Moderate |
|  |  |  | Asian/ | 13 | 874/645 | Heterozygous | 0.849 (0.652-1.106) | 0.226 | 0.0 | 0.9580 | 0.113 | 0.318 | BAA | Moderate |
|  |  |  | European | 20 | 1516/950 | Dominant | 0.777 (0.543-0.112) | 0.168 | 69.3 | 0.0001 | 0.243 | 0.114 | ABA | Moderate |
|  |  |  |  | 7 | 461/360 | Recessive | 0.658 (0.310-1.397) | 0.276 | 65.6 | 0.0001 | 0.881 | 0.463 | BBA | Moderate |
|  |  |  |  | 13 | 874/645 | Allele | 0.917 (0.643-1.308) | 0.631 | 68.8 | 0.0001 | 0.542 | 0.421 | BBA | Moderate |
|  |  |  | Asian | 2 | 587/284 | Dominant | 1.460 (0.997-2.137) | 0.052 | 0.0 | 0.9250 | 0.317 | - | BAB | Moderate |
|  | rs1799793 (G/A) | Good | Asian/European | 10 | 677/531 | Heterozygous | 1.156 (0.893-1.497) | 0.272 | 0.0 | 0.7400 | 0.655 | 0.997 | BAA | Moderate |
|  | rs238406 (C/A) | Good | Asian | 3 | 809/346 | Dominant | 1.399 (0.813-2.406) | 0.225 | 71.8 | 0.0290 | 0.602 | 0.668 | BBA | Moderate |
|  |  |  |  | 1 | 222/62 | Homozygous | 1.108 (0.490-2.505) | 0.806 | - | - | - | - | BCC | Weak |
|  |  |  |  | 1 | 222/62 | Heterozygous | 1.299 (0.670-2.518) | 0.439 | - | - | - | - | BCC | Weak |
|  |  |  |  | 1 | 222/62 | Recessive | 0.932 (0.465-1.867) | 0.842 | - | - | - | - | BCC | Weak |
|  |  |  |  | 1 | 222/62 | Allele | 1.064 (0.714-1.587) | 0.759 | - | - | - | - | BCC | Weak |
|  | rs1305686667 (G/A) | Good | Asian | 1 | 56/52 | Heterozygous | 0.923 (0.219-3.897) | 0.913 | - | - | - | - | CCC | Weak |
|  |  |  |  | 1 | 56/52 | Dominant | 0.923 (0.219-3.897) | 0.913 | - | - | - | - | BCC | Weak |
|  |  |  |  | 1 | 56/52 | Allele | 0.923 (0.219-3.897) | 0.913 | - | - | - | - | BCC | Weak |
| *ERCC3* | rs4150454 (T/C) | Good | European | 1 | 72/89 | Heterozygous | 0.716 (0.362-1.414) | 0.335 | - | - | - | - | CCC | Weak |
|  |  |  |  | 1 | 72/89 | Dominant | 0.551 (0.301-1.008) | 0.053 | - | - | - | - | CCC | Weak |
|  |  |  |  | 1 | 72/89 | Heterozygous | 0.835 (0.385-1.812) | 0.649 | - | - | - | - | CCC | Weak |
|  | rs3738948 (A/G) | Good | European | 1 | 72/89 | Homozygous | 1.763 (0.625-4.973) | 0.284 | - | - | - | - | CCC | Weak |
|  |  |  |  | 1 | 72/89 | Heterozygous | 0.748 (0.384-1.456) | 0.393 | - | - | - | - | CCC | Weak |
|  |  |  |  | 1 | 72/89 | Dominant | 0.913 (0.494-1.686) | 0.722 | - | - | - | - | CCC | Weak |
|  |  |  |  | 1 | 72/89 | Recessive | 1.982 (0.728-5.399) | 0.181 | - | - | - | - | CCC | Weak |
|  |  |  |  | 1 | 72/89 | Allele | 1.111 (0.690-1.788) | 0.665 | - | - | - | - | CCC | Weak |
| *ERCC4 (XPF)* | rs2276465 (C/G) | Good | Asian | 1 | 148/92 | Homozygous | 0.678 (0.333-1.379) | 0.283 | - | - | - | - | BCC | Weak |
|  |  |  |  | 1 | 148/92 | Heterozygous | 0.844 (0.450-1.580) | 0.595 | - | - | - | - | CCC | Weak |
|  |  |  |  | 1 | 148/92 | Dominant | 0.771 (0.445-1.306) | 0.333 | - | - | - | - | CCC | Weak |
|  |  |  |  | 1 | 148/92 | Recessive | 0.712 (0.359-1.414) | 0.332 | - | - | - | - | CCC | Weak |
|  |  |  |  | 1 | 148/92 | Allele | 0.764 (0.511-1.143) | 0.190 | - | - | - | - | BCC | Weak |
|  | rs1799801 (T/C) | Good | European | 1 | 72/89 | Homozygous | 1.276 (0.604-2.696) | 0.524 | - | - | - | - | CCC | Weak |
|  |  |  |  | 1 | 72/89 | Dominant | 0.742 (0.390-1.410) | 0.362 | - | - | - | - | CCC | Weak |
|  |  |  |  | 1 | 72/89 | Recessive | 1.814 (0.907-3.629) | 0.092 | - | - | - | - | CCC | Weak |
|  |  |  |  | 1 | 72/89 | Allele | 1.115 (0.708-1.755) | 0.640 | - | - | - | - | BCC | Weak |
|  | rs6498486 (A/C) | Good | Asian | 1 | 148/92 | Homozygous | 0.684 (0.320-1.460) | 0.326 | - | - | - | - | BCC | Weak |
|  |  |  |  | 1 | 148/92 | Heterozygous | 0.855 (0.445-1.642) | 0.638 | - | - | - | - | CCC | Weak |
|  |  |  |  | 1 | 148/92 | Dominant | 0.782 (0.454-1.346) | 0.375 | - | - | - | - | CCC | Weak |
|  |  |  |  | 1 | 148/92 | Recessive | 0.711 (0.339-1.491) | 0.366 | - | - | - | - | CCC | Weak |
|  |  |  |  | 1 | 148/92 | Allele | 0.769 (0.503-1.176) | 0.226 | - | - | - | - | BCC | Weak |
| *ERCC5 (XPG)* | rs1047768 (C/T) | Good | Asian/ | 3 | 391/262 | Homozygous | 1.028 (0.415-2.547) | 0.953 | 72.3 | 0.0270 | 0.117 | 0.093 | BBA | Moderate |
|  |  |  | European | 3 | 391/262 | Heterozygous | 0.869 (0.511-1.478) | 0.603 | 36.7 | 0.2060 | 0.602 | 0.408 | BBA | Moderate |
|  |  |  |  | 3 | 391/262 | Dominant | 0.916 (0.484-1.732) | 0.788 | 62.0 | 0.0720 | 0.117 | 0.198 | BBA | Moderate |
|  |  |  |  | 3 | 391/262 | Recessive | 1.000 (0.517-1.934) | 1.000 | 64.5 | 0.0600 | 0.117 | 0.017 | BBA | Moderate |
|  |  |  |  | 3 | 391/262 | Allele | 0.971 (0.584-1.617) | 0.911 | 75.4 | 0.0170 | 0.117 | 0.063 | BBA | Moderate |
|  | rs17655 (C/G) | Good | Asian/ | 5 | 886/498 | Homozygous | 0.823 (0.590-1.148) | 0.251 | 0.0 | 0.4270 | 1.000 | 0.679 | BAA | Moderate |
|  |  |  | European | 5 | 886/498 | Heterozygous | 0.955 (0.744-1.225) | 0.716 | 0.0 | 0.9550 | 0.624 | 0.952 | BAA | Moderate |
|  |  |  |  | 5 | 886/498 | Dominant | 0.920 (0.730-1.160) | 0.481 | 0.0 | 0.8060 | 0.624 | 0.612 | BAA | Moderate |
|  |  |  |  | 5 | 886/498 | Recessive | 0.826 (0.612-1.116) | 0.213 | 0.0 | 0.3960 | 1.000 | 0.578 | BAA | Moderate |
|  |  |  |  | 5 | 886/498 | Allele | 1.052 (0.758-1.461) | 0.761 | 67.5 | 0.0150 | 0.142 | 0.350 | BBA | Moderate |
|  | rs2094258 (A/G) | Good | Asian | 2 | 450/229 | Homozygous | 0.886 (0.536-1.464) | 0.637 | 0.0 | 0.4940 | 0.317 | - | BAB | Moderate |
|  |  |  |  | 2 | 450/229 | Heterozygous | 1.020 (0.653-1.594) | 0.930 | 14.2 | 0.2800 | 0.317 | - | BAB | Moderate |
|  |  |  |  | 2 | 450/229 | Dominant | 0.939 (0.651-1.355) | 0.738 | 2.1 | 0.3120 | 0.317 | - | BAB | Moderate |
|  |  |  |  | 2 | 450/229 | Recessive | 0.821 (0.540-1.248) | 0.356 | 0.0 | 0.9170 | 0.317 | - | BAB | Moderate |
|  |  |  |  | 2 | 450/229 | Allele | 0.904 (0.705-1.160) | 0.426 | 0.0 | 0.5330 | 0.317 | - | BAB | Moderate |
|  | rs2296147 (C/T) | Good | Asian | 2 | 450/228 | Homozygous | 0.655 (0.373-1.151) | 0.142 | 0.0 | 0.5160 | 0.317 | - | BAB | Moderate |
|  |  |  |  | 2 | 450/228 | Heterozygous | 0.838 (0.544-1.290) | 0.421 | 0.0 | 0.4400 | 0.317 | - | BAB | Moderate |
|  |  |  |  | 2 | 450/228 | Dominant | 0.789 (0.534-1.166) | 0.234 | 0.0 | 0.4140 | 0.317 | - | BAB | Moderate |
|  |  |  |  | 2 | 450/228 | Recessive | 0.779 (0.522-1.164) | 0.223 | 0.0 | 0.7970 | 0.317 | - | BAB | Moderate |
|  |  |  |  | 2 | 450/228 | Allele | 0.808 (0.623-1.048) | 0.108 | 0.0 | 0.8970 | 0.317 | - | BAB | Moderate |
|  | rs2016073 (G/A) | Good | Asian | 1 | 100/42 | Homozygous | 2.118 (0.534-8.397) | 0.286 | - | - | - | - | CCC | Weak |
|  |  |  |  | 1 | 100/42 | Heterozygous | 1.270 (0.322-5.002) | 0.733 | - | - | - | - | CCC | Weak |
|  |  |  |  | 1 | 100/42 | Dominant | 1.649 (0.441-6.174) | 0.458 | - | - | - | - | CCC | Weak |
|  |  |  |  | 1 | 100/42 | Recessive | 1.726 (0.831-3.586) | 0.143 | - | - | - | - | CCC | Weak |
|  |  |  |  | 1 | 100/42 | Allele | 1.501 (0.866-2.600) | 0.148 | - | - | - | - | CCC | Weak |
|  | rs11069498 (A/G) | Good | Asian | 1 | 100/42 | Homozygous | 0.303 (0.062-1.472) | 0.139 | - | - | - | - | CCC | Weak |
|  |  |  |  | 1 | 100/42 | Heterozygous | 0.615 (0.121-3.137) | 0.559 | - | - | - | - | CCC | Weak |
|  |  |  |  | 1 | 100/42 | Dominant | 0.405 (0.086-1.910) | 0.253 | - | - | - | - | CCC | Weak |
|  | rs4150330 (G/A) | Good | Asian | 1 | 100/42 | Homozygous | 0.562 (0.110-2.877) | 0.489 | - | - | - | - | CCC | Weak |
|  |  |  |  | 1 | 100/42 | Heterozygous | 0.935 (0.169-5.172) | 0.939 | - | - | - | - | CCC | Weak |
|  |  |  |  | 1 | 100/42 | Dominant | 0.664 (0.132-3.339) | 0.620 | - | - | - | - | CCC | Weak |
|  |  |  |  | 1 | 100/42 | Recessive | 0.594 (0.277-1.277) | 0.182 | - | - | - | - | CCC | Weak |
|  |  |  |  | 1 | 100/42 | Allele | 0.652 (0.343-1.241) | 0.193 | - | - | - | - | CCC | Weak |
|  | rs873601 (A/G) | Good | Asian | 2 | 414/179 | Homozygous | 1.108 (0.669-1.833) | 0.691 | 0.0 | 0.7320 | 0.317 |  | BAB | Weak |
|  |  |  |  | 2 | 414/179 | Heterozygous | 1.047 (0.702-1.561) | 0.821 | 0.0 | 0.9100 | 0.317 |  | BAB | Weak |
|  |  |  |  | 2 | 414/179 | Dominant | 1.064 (0.737-1.535) | 0.742 | 0.0 | 0.9510 | 0.317 |  | BAB | Weak |
|  |  |  |  | 2 | 414/179 | Recessive | 1.104 (0.704-1.732) | 0.666 | 0.0 | 0.6360 | 0.317 |  | BAB | Weak |
|  |  |  |  | 2 | 414/179 | Allele | 1.065 (0.824-1.376) | 0.630 | 0.0 | 0.7560 | 0.317 |  | BAB | Weak |
|  | rs4771436 (T/G) | Good | Asian | 1 | 100/42 | Homozygous | 0.470 (0.120-1.867) | 0.286 | - | - | - | - | CCC | Weak |
|  |  |  |  | 1 | 100/42 | Heterozygous | 0.584 (0.273-1.248) | 0.165 | - | - | - | - | CCC | Weak |
|  |  |  |  | 1 | 100/42 | Dominant | 0.566 (0.273-1.172) | 0.125 | - | - | - | - | CCC | Weak |
|  |  |  |  | 1 | 100/42 | Recessive | 0.606 (0.162-2.270) | 0.458 | - | - | - | - | CCC | Weak |
|  |  |  |  | 1 | 100/42 | Allele | 0.649 (0.372-1.132) | 0.128 | - | - | - | - | CCC | Weak |
|  | rs1047768 (T/C) | Good | Asian | 3 | 619/307 | Homozygous | 0.629 (0.170-2.329) | 0.488 | 88.5 | 0.0001 | 0.602 | 0.470 | BCA | Weak |
|  |  |  |  | 3 | 619/307 | Heterozygous | 0.642 (0.238-1.732) | 0.382 | 80.8 | 0.0050 | 0.117 | 0.267 | BCA | Weak |
|  |  |  |  | 3 | 619/307 | Dominant | 0.622 (0.194-1.988) | 0.423 | 88.3 | 0.0001 | 0.602 | 0.299 | BCA | Weak |
|  |  |  |  | 3 | 619/307 | Recessive | 0.893 (0.431-1.852) | 0.762 | 80.5 | 0.0060 | 0.602 | 0.872 | BCA | Weak |
|  |  |  |  | 3 | 619/307 | Allele | 0.987 (0.499-1.950) | 0.970 | 88.2 | 0.0001 | 0.602 | 0.962 | BCA | Weak |
|  | rs751402 (A/G) | Good | Asian | 1 | 135/92 | Homozygous | 2.455 (0.895-6.729) | 0.081 | - | - | - | - | CCC | Weak |
|  |  |  |  | 1 | 135/92 | Recessive | 0.955 (0.561-1.623) | 0.864 | - | - | - | - | BCC | Weak |
|  |  |  |  | 1 | 135/92 | Allele | 1.170 (0.781-1.753) | 0.447 | - | - | - | - | BCC | Weak |
| *FGFR4* | rs351855 (G/A) | Good | Asian | 1 | 338/291 | Dominant | 1.022 (0.727-1.435) | 0.902 | - | - | - | - | BCC | Weak |
|  |  |  |  | 1 | 338/291 | Heterozygous | 1.422 (0.992-2.037) | 0.055 | - | - | - | - | BCC | Weak |
| *FOK1* | rs10735810 (C/T) | Good | Asian | 1 | 434/321 | Homozygous | 0.852 (0.542-1.340) | 0.448 | - | - | - | - | BCC | Weak |
|  |  |  |  | 1 | 434/321 | Heterozygous | 0.745 (0.533-1.043) | 0.086 | - | - | - | - | BCC | Weak |
|  |  |  |  | 1 | 434/321 | Dominant | 0.768 (0.556-1.061) | 0.110 | - | - | - | - | BCC | Weak |
|  |  |  |  | 1 | 434/321 | Recessive | 1.035 (0.699-1.534) | 0.862 | - | - | - | - | BCC | Weak |
|  |  |  |  | 1 | 434/321 | Allele | 0.906 (0.738-1.113) | 0.347 | - | - | - | - | BCC | Weak |
| *FRAP1* | rs12139042 (G/A) | Good | Asian | 1 | 156/41 | Homozygous | 1.943 (0.171-22.047) | 0.592 | - | - | - | - | CCC | Weak |
|  |  |  |  | 1 | 156/41 | Heterozygous | 1.079 (0.375-3.110) | 0.888 | - | - | - | - | CCC | Weak |
|  |  |  |  | 1 | 156/41 | Dominant | 1.166 (0.435-3.122) | 0.760 | - | - | - | - | CCC | Weak |
|  |  |  |  | 1 | 156/41 | Recessive | 1.925 (0.170-21.768) | 0.597 | - | - | - | - | CCC | Weak |
|  |  |  |  | 1 | 156/41 | Allele | 1.230 (0.506-2.989) | 0.647 | - | - | - | - | CCC | Weak |
| *GPI* | rs7248411 (C/G) | Good | Asian | 1 | 193/174 | Homozygous | 0.389 (0.114-1.327) | 0.131 | - | - | - | - | CCC | Weak |
|  |  |  |  | 1 | 193/174 | Heterozygous | 0.689 (0.441-1.076) | 0.101 | - | - | - | - | BCC | Weak |
|  |  |  |  | 1 | 193/174 | Dominant | 0.665 (0.432-1.023) | 0.064 | - | - | - | - | BCC | Weak |
|  |  |  |  | 1 | 193/174 | Recessive | 0.439 (0.130-1.485) | 0.186 | - | - | - | - | CCC | Weak |
| *GSTP1* | rs1695 (A/G) | Good | Asian/ | 13 | 1237/1389 | Heterozygous | 0.819 (0.590-1.137) | 0.233 | 68.3 | 0.0001 | 0.542 | 0.781 | ABA | Moderate |
|  |  |  | European | 12 | 1159/1352 | Homozygous | 0.717 (0.347-1.485) | 0.371 | 84.3 | 0.0001 | 0.891 | 0.696 | BCA | Weak |
|  |  |  |  | 15 | 1382/1452 | Dominant | 0.821 (0.550-1.225) | 0.334 | 82.8 | 0.0001 | 0.656 | 0.767 | ACA | Weak |
|  |  |  |  | 12 | 1159/1352 | Recessive | 0.794 (0.443-1.425) | 0.440 | 78.5 | 0.0001 | 0.891 | 0.695 | BCA | Weak |
|  |  |  |  | 13 | 1237/1389 | Allele | 0.834 (0.588-1.183) | 0.308 | 86.8 | 0.0001 | 0.464 | 0.714 | ACA | Weak |
|  | rs1138272 (C/T) | Good | Asian/ | 5 | 429/513 | Homozygous | 0.649 (0.140-3.004) | 0.500 | 88.4 | 0.0001 | 0.327 | 0.155 | BCA | Weak |
|  |  |  | European | 6 | 457/571 | Heterozygous | 0.718 (0.379-1.360) | 0.390 | 78.7 | 0.0001 | 0.851 | 0.648 | BCA | Weak |
|  |  |  |  | 7 | 533/606 | Dominant | 0.647 (0.311-1.349) | 0.246 | 86.8 | 0.0001 | 0.881 | 0.488 | BCA | Weak |
|  |  |  |  | 6 | 457/571 | Recessive | 0.756 (0.243-2.353) | 0.629 | 81.0 | 0.0001 | 0.624 | 0.111 | BCA | Weak |
|  |  |  |  | 6 | 457/571 | Allele | 0.769 (0.339-1.482) | 0.433 | 89.4 | 0.0001 | 0.851 | 0.406 | BCA | Weak |
| *GSTT1* | Deletion mutation | Good | Asian | 3 | 457/331 | Present/Null | 0.896 (0.668-1.201) | 0.462 | 0.0 | 0.9800 | 0.602 | 0.594 | BAA | Moderate |
| *H19* | rs2839698 (G/A) | Good | Asian | 1 | 183/282 | Homozygous | 0.836 (0.396-1.763) | 0.637 | - | - | - | - | CCC | Weak |
|  |  |  |  | 1 | 183/282 | Heterozygous | 0.681 (0.461-1.004) | 0.053 | - | - | - | - | BCC | Weak |
|  |  |  |  | 1 | 183/282 | Dominant | 0.701 (0.482-1.018) | 0.062 | - | - | - | - | BCC | Weak |
|  |  |  |  | 1 | 183/282 | Recessive | 1.002 (0.552-1.817) | 0.995 | - | - | - | - | CCC | Weak |
|  |  |  |  | 1 | 183/282 | Allele | 0.806 (0.602-1.079) | 0.148 | - | - | - | - | BCC | Weak |
| *HOGG1* | rs1052133 (C/G) | Good | Asian | 1 | 95/53 | Homozygous | 0.467 (0.154-1.413) | 0.177 | - | - | - | - | CCC | Weak |
|  |  |  |  | 1 | 95/53 | Heterozygous | 0.423 (0.138-1.298) | 0.132 | - | - | - | - | CCC | Weak |
|  |  |  |  | 1 | 95/53 | Dominant | 0.446 (0.155-1.279) | 0.133 | - | - | - | - | CCC | Weak |
|  |  |  |  | 1 | 95/53 | Recessive | 0.888 (0.452-1.742) | 0.729 | - | - | - | - | CCC | Weak |
|  |  |  |  | 1 | 95/53 | Allele | 0.758 (0.457-1.256) | 0.282 | - | - | - | - | CCC | Weak |
| *HOTAIR* | rs7598904 (C/G) | Good | Asian | 1 | 184/279 | Homozygous | 0.615 (0.288-1.317) | 0.211 | - | - | - | - | CCC | Weak |
|  |  |  |  | 1 | 184/279 | Heterozygous | 1.005 (0.471-2.143) | 0.990 | - | - | - | - | CCC | Weak |
|  |  |  |  | 1 | 184/279 | Dominant | 0.788 (0.378-1.640) | 0.524 | - | - | - | - | CCC | Weak |
| *iASP* | A67T | Good | Asian | 1 | 50/26 | Homozygous | 0.513 (0.101-2.614) | 0.423 | - | - | - | - | CCC | Weak |
|  |  |  |  | 1 | 50/26 | Heterozygous | 0.286 (0.071-1.146) | 0.077 | - | - | - | - | CCC | Weak |
|  |  |  |  | 1 | 50/26 | Dominant | 0.335 (0.087-1.297) | 0.113 | - | - | - | - | CCC | Weak |
|  |  |  |  | 1 | 50/26 | Recessive | 1.326 (0.411-4.280) | 0.637 | - | - | - | - | CCC | Weak |
|  |  |  |  | 1 | 50/26 | Allele | 0.791 (0.404-1.549) | 0.494 | - | - | - | - | CCC | Weak |
| *IGF-1R* | G1013A | Good | Asian | 1 | 32/100 | Homozygous | 0.841 (0.201-3.525) | 0.813 | - | - | - | - | CCC | Weak |
|  |  |  |  | 1 | 32/100 | Heterozygous | 1.008 (0.431-2.360) | 0.985 | - | - | - | - | CCC | Weak |
|  |  |  |  | 1 | 32/100 | Dominant | 0.979 (0.430-2.229) | 0.959 | - | - | - | - | CCC | Weak |
|  |  |  |  | 1 | 32/100 | Recessive | 0.837 (0.218-3.208) | 0.795 | - | - | - | - | CCC | Weak |
|  |  |  |  | 1 | 32/100 | Allele | 0.955 (0.532-1.716) | 0.878 | - | - | - | - | CCC | Weak |
| *IGF-2R* | G1619A | Good | Asian | 1 | 32/100 | Homozygous | 1.017 (0.100-10.330) | 0.989 | - | - | - | - | CCC | Weak |
|  |  |  |  | 1 | 32/100 | Heterozygous | 0.932 (0.401-2.166) | 0.870 | - | - | - | - | CCC | Weak |
|  |  |  |  | 1 | 32/100 | Dominant | 0.938 (0.413-2.132) | 0.879 | - | - | - | - | CCC | Weak |
|  |  |  |  | 1 | 32/100 | Recessive | 1.043 (0.105-10.393) | 0.971 | - | - | - | - | CCC | Weak |
|  |  |  |  | 1 | 32/100 | Allele | 0.959 (0.477-1.926) | 0.906 | - | - | - | - | CCC | Weak |
| *LGALS3* | rs4644 (A/C) | Good | Asian | 1 | 201/119 | Homozygous | 1.531 (0.809-2.897) | 0.191 | - | - | - | - | CCC | Weak |
|  |  |  |  | 1 | 201/119 | Heterozygous | 1.018 (0.615-1.683) | 0.946 | - | - | - | - | BCC | Weak |
|  |  |  |  | 1 | 201/119 | Dominant | 1.165 (0.737-1.842) | 0.513 | - | - | - | - | BCC | Weak |
|  |  |  |  | 1 | 201/119 | Recessive | 1.518 (0.840-2.745) | 0.167 | - | - | - | - | CCC | Weak |
|  |  |  |  | 1 | 201/119 | Allele | 1.240 (0.890-1.728) | 0.203 | - | - | - | - | BCC | Weak |
| *MALAT* | rs619586 (A/G) | Good | Asian | 1 | 178/279 | Homozygous | 1.703 (0.237-12.226) | 0.597 | - | - | - | - | CCC | Weak |
|  |  |  |  | 1 | 178/279 | Heterozygous | 1.541 (0.947-2.506) | 0.082 | - | - | - | - | CCC | Weak |
|  |  |  |  | 1 | 178/279 | Dominant | 1.548 (0.961-2.494) | 0.073 | - | - | - | - | BCC | Weak |
|  |  |  |  | 1 | 178/279 | Recessive | 1.574 (0.220-11.275) | 0.652 | - | - | - | - | CCC | Weak |
|  |  |  |  | 1 | 178/279 | Allele | 1.489 (0.958-2.315) | 0.077 | - | - | - | - | BCC | Weak |
| *MATE1* | rs2289669 (G/A) | Good | Asian | 1 | 267/108 | Homozygous | 1.573 (0.809-3.055) | 0.182 | - | - | - | - | CCC | Weak |
|  |  |  |  | 1 | 267/108 | Heterozygous | 1.168 (0.686-1.989) | 0.568 | - | - | - | - | CCC | Weak |
|  |  |  |  | 1 | 267/108 | Dominant | 1.272 (0.765-2.113) | 0.354 | - | - | - | - | CCC | Weak |
|  |  |  |  | 1 | 267/108 | Recessive | 1.416 (0.809-2.479) | 0.224 | - | - | - | - | CCC | Weak |
|  |  |  |  | 1 | 267/108 | Allele | 1.231 (0.897-1.691) | 0.198 | - | - | - | - | BCC | Weak |
| *MDM2* | rs2279744 (T/G) | Good | Asian | 1 | 332/72 | Homozygous | 1.798 (0.569-5.690) | 0.318 | - | - | - | - | CCC | Weak |
|  |  |  |  | 1 | 332/72 | Heterozygous | 2.376 (0.787-7.151) | 0.125 | - | - | - | - | CCC | Weak |
|  |  |  |  | 1 | 332/72 | Dominant | 2.178 (0.733-6.473) | 0.171 | - | - | - | - | CCC | Weak |
|  |  |  |  | 1 | 332/72 | Recessive | 0.804 (0.464-1.396) | 0.439 | - | - | - | - | BCC | Weak |
|  |  |  |  | 1 | 332/72 | Allele | 0.981 (0.676-1.423) | 0.919 | - | - | - | - | CCC | Weak |
|  | rs937282 (C/G) | Good | Asian | 1 | 331/70 | Homozygous | 1.556 (0.572-4.232) | 0.387 | - | - | - | - | BCC | Weak |
|  |  |  |  | 1 | 331/70 | Heterozygous | 1.029 (0.597-1.774) | 0.919 | - | - | - | - | BCC | Weak |
|  |  |  |  | 1 | 331/70 | Dominant | 1.111 (0.662-1.864) | 0.690 | - | - | - | - | BCC | Weak |
|  |  |  |  | 1 | 331/70 | Recessive | 1.537 (0.580-4.074) | 0.387 | - | - | - | - | CCC | Weak |
|  |  |  |  | 1 | 331/70 | Allele | 1.162 (0.771-1.754) | 0.473 | - | - | - | - | BCC | Weak |
| *MMP2* | rs12934241 (C/T) | Good | Asian | 1 | 537/129 | Homozygous | 1.554 (0.450-5.363) | 0.485 | - | - | - | - | CCC | Weak |
|  |  |  |  | 1 | 537/129 | Heterozygous | 1.421 (0.872-2.318) | 0.159 | - | - | - | - | BCC | Weak |
|  |  |  |  | 1 | 537/129 | Dominant | 1.436 (0.900-2.290) | 0.129 | - | - | - | - | BCC | Weak |
|  |  |  |  | 1 | 537/129 | Recessive | 1.431 (0.416-4.914) | 0.570 | - | - | - | - | CCC | Weak |
|  |  |  |  | 1 | 537/129 | Allele | 1.381 (0.912-2.092) | 0.127 | - | - | - | - | BCC | Weak |
| *MSH2* | gIVS112-6 | Good | Asian | 1 | 68/28 | Heterozygous | 0.607 (0.238-1.553) | 0.298 | - | - | - | - | CCC | Weak |
|  |  |  |  | 1 | 68/28 | Dominant | 0.481 (0.196-1.181) | 0.110 | - | - | - | - | CCC | Weak |
| *MSH3* | rs26279 (G/A) | Good | Asian | 1 | 103/77 | Homozygous | 0.190 (0.023-1.559) | 0.122 | - | - | - | - | CCC | Weak |
|  |  |  |  | 1 | 103/77 | Dominant | 0.137 (0.017-0.109) | 0.062 | - | - | - | - | CCC | Weak |
|  |  |  |  | 1 | 103/77 | Recessive | 1.756 (0.964-3.198) | 0.066 | - | - | - | - | CCC | Weak |
|  |  |  |  | 1 | 103/77 | Allele | 1.187 (0.731-1.929) | 0.489 | - | - | - | - | CCC | Weak |
|  | rs1650697 (A/G) | Good | Asian | 1 | 102/77 | Recessive | 0.757 (0.417-1.374) | 0.359 | - | - | - | - | CCC | Weak |
|  | rs1105524 (A/G) | Good | Asian | 1 | 103/77 | Heterozygous | 1.125 (0.584-2.166) | 0.724 | - | - | - | - | CCC | Weak |
|  |  |  |  | 1 | 103/77 | Dominant | 1.502 (0.824-2.738) | 0.184 | - | - | - | - | CCC | Weak |
| *MTHFR* | rs1801133 (C/T) | Good | Asian/ | 6 | 466/1003 | Homozygous | 1.635 (0.590-4.532) | 0.345 | 78.4 | 0.0001 | 0.573 | 0.513 | BBA | Moderate |
|  |  |  | European | 6 | 466/1003 | Heterozygous | 1.067 (0.798-1.427) | 0.661 | 46.3 | 0.0980 | 0.348 | 0.894 | BBA | Moderate |
|  |  |  |  | 7 | 474/1006 | Dominant | 1.126 (0.864-1.468) | 0.381 | 32.6 | 0.1790 | 0.652 | 0.416 | BBA | Moderate |
|  |  |  |  | 6 | 466/1003 | Allele | 1.253 (0.788-1.993) | 0.341 | 77.0 | 0.0100 | 0.851 | 0.479 | ABA | Moderate |
|  |  |  |  | 6 | 466/1003 | Recessive | 1.551 (0.561-4.287) | 0.398 | 84.3 | 0.0001 | 0.188 | 0.474 | BCA | Weak |
|  | rs1801131 (C/T) | Poor | Asian | 1 | 191/563 | Homozygous | 1.623 (0.655-4.021) | 0.296 | - | - | - | - | CCC | Weak |
|  |  |  |  | 1 | 191/563 | Dominant | 0.711 (0.498-1.014) | 0.059 | - | - | - | - | BCC | Weak |
|  |  |  |  | 1 | 191/563 | Recessive | 1.826 (0.740-4.503) | 0.191 | - | - | - | - | CCC | Weak |
|  |  |  |  | 1 | 191/563 | Allele | 0.817 (0.600-1.114) | 0.202 | - | - | - | - | BCC | Weak |
|  | rs1801131 (A/C)) | Good | Asian | 1 | 58/38 | Homozygous | 1.647 (0.281-9.665) | 0.580 | - | - | - | - | CCC | Weak |
|  |  |  |  | 1 | 58/38 | Heterozygous | 2.059 (0.788-5.379) | 0.141 | - | - | - | - | CCC | Weak |
|  |  |  |  | 1 | 58/38 | Dominant | 1.976 (0.811-4.819) | 0.134 | - | - | - | - | CCC | Weak |
|  |  |  |  | 1 | 58/38 | Recessive | 1.111 (0.192-6.427) | 0.906 | - | - | - | - | CCC | Weak |
|  |  |  |  | 1 | 58/38 | Allele | 1.697 (0.802-3.589) | 0.166 | - | - | - | - | CCC | Weak |
|  | rs1537514 (G/C) | Good | Asian | 1 | 757/362 | Homozygous | 1.539 (0.394-6.017) | 0.535 | - | - | - | - | CCC | Weak |
|  |  |  |  | 1 | 757/362 | Recessive | 0.895 (0.230-3.483) | 0.873 | - | - | - | - | CCC | Weak |
| *MTR* | A2756G | Good | Asian | 1 | 70/31 | Heterozygous | 0.719 (0.266-1.944) | 0.515 | - | - | - | - | CCC | Weak |
|  |  |  |  | 1 | 70/31 | Dominant | 0.719 (0.266-1.944) | 0.515 | - | - | - | - | CCC | Weak |
|  |  |  |  | 1 | 70/31 | Allele | 0.750 (0.297-1.892) | 0.542 | - | - | - | - | CCC | Weak |
| *MUTYH* | rs3219489 (G/C) | Good | Asian | 1 | 97/55 | Homozygous | 2.049 (0.605-6.940) | 0.249 | - | - | - | - | CCC | Weak |
|  |  |  |  | 1 | 97/55 | Heterozygous | 0.911 (0.453-1.830) | 0.793 | - | - | - | - | CCC | Weak |
|  |  |  |  | 1 | 97/55 | Dominant | 1.057 (0.542-2.062) | 0.870 | - | - | - | - | CCC | Weak |
|  |  |  |  | 1 | 97/55 | Recessive | 2.151 (0.671-6.892) | 0.198 | - | - | - | - | CCC | Weak |
|  |  |  |  | 1 | 97/55 | Allele | 1.210 (0.736-1.988) | 0.453 | - | - | - | - | CCC | Weak |
| *NBS* | rs1805794 (A/G) | Good | Asian | 1 | 89/52 | Homozygous | 1.422 (0.571-3.544) | 0.450 | - | - | - | - | CCC | Weak |
|  |  |  |  | 1 | 89/52 | Heterozygous | 1.043 (0.444-2.454) | 0.922 | - | - | - | - | CCC | Weak |
|  |  |  |  | 1 | 89/52 | Dominant | 1.193 (0.545-2.613) | 0.659 | - | - | - | - | CCC | Weak |
|  |  |  |  | 1 | 89/52 | Recessive | 1.385 (0.661-2.902) | 0.388 | - | - | - | - | CCC | Weak |
|  |  |  |  | 1 | 89/52 | Allele | 1.234 (0.759-2.004) | 0.396 | - | - | - | - | CCC | Weak |
|  | rs13312840 (T/C) | Good | Asian | 1 | 93/52 | Heterozygous | 1.000 (0.456-2.193) | 1.000 | - | - | - | - | CCC | Weak |
|  |  |  |  | 1 | 93/52 | Dominant | 1.043 (0.478-2.278) | 0.915 | - | - | - | - | CCC | Weak |
|  |  |  |  | 1 | 93/52 | Allele | 1.087 (0.530-0.228) | 0.820 | - | - | - | - | CCC | Weak |
| *NF-kB* | rs230521 (C/G) | Good | Asian | 1 | 149/113 | Homozygous | 1.154 (0.570-2.335) | 0.691 | - | - | - | - | CCC | Weak |
|  |  |  |  | 1 | 149/113 | Heterozygous | 0.782 (0.430-1.421) | 0.419 | - | - | - | - | CCC | Weak |
|  |  |  |  | 1 | 149/113 | Dominant | 0.886 (0.503-1.560) | 0.674 | - | - | - | - | CCC | Weak |
|  |  |  |  | 1 | 149/113 | Recessive | 1.361 (0.763-2.427) | 0.296 | - | - | - | - | CCC | Weak |
|  |  |  |  | 1 | 149/113 | Allele | 1.069 (0.756-1.510) | 0.706 | - | - | - | - | BCC | Weak |
|  | rs4648068 (A/G) | Good | Asian | 1 | 149/113 | Homozygous | 0.904 (0.441-1.850) | 0.782 | - | - | - | - | CCC | Weak |
|  |  |  |  | 1 | 149/113 | Heterozygous | 0.738 (0.419-1.301) | 0.294 | - | - | - | - | CCC | Weak |
|  |  |  |  | 1 | 149/113 | Dominant | 0.781 (0.457-1.336) | 0.367 | - | - | - | - | CCC | Weak |
|  |  |  |  | 1 | 149/113 | Recessive | 1.092 (0.587-2.033) | 0.780 | - | - | - | - | CCC | Weak |
|  |  |  |  | 1 | 149/113 | Allele | 0.926 (0.653-1.312) | 0.664 | - | - | - | - | BCC | Weak |
| *OCT2.* | rs316003 (C/T) | Good | Asian | 1 | 272/108 | Homozygous | 0.982 (0.337-2.865) | 0.974 | - | - | - | - | CCC | Weak |
|  |  |  |  | 1 | 272/108 | Heterozygous | 0.941 (0.314-2.823) | 0.914 | - | - | - | - | CCC | Weak |
|  |  |  |  | 1 | 272/108 | Dominant | 0.967 (0.336-2.781) | 0.951 | - | - | - | - | CCC | Weak |
|  |  |  |  | 1 | 272/108 | Recessive | 1.036 (0.657-1.634) | 0.879 | - | - | - | - | BCC | Weak |
|  | rs316019 (T/C) | Good | Asian | 1 | 272/108 | Homozygous | 0.712 (0.145-3.504) | 0.677 | - | - | - | - | CCC | Weak |
|  |  |  |  | 1 | 272/108 | Heterozygous | 0.720 (0.140-3.685) | 0.693 | - | - | - | - | CCC | Weak |
|  |  |  |  | 1 | 272/108 | Dominant | 0.714 (0.146-3.494) | 0.678 | - | - | - | - | CCC | Weak |
|  |  |  |  | 1 | 272/108 | Recessive | 0.964 (0.584-1.593) | 0.887 | - | - | - | - | CCC | Weak |
|  | rs1869641 (G/A) | Good | Asian | 1 | 149/184 | Homozygous | 1.680 (0.546-5.165) | 0.365 | - | - | - | - | CCC | Weak |
|  |  |  |  | 1 | 149/184 | Heterozygous | 1.604 (0.967-2.661) | 0.067 | - | - | - | - | BCC | Weak |
|  |  |  |  | 1 | 149/184 | Recessive | 1.481 (0.486-4.518) | 0.490 | - | - | - | - | CCC | Weak |
|  |  |  |  | 1 | 149/184 | Allele | 1.508 (0.996-2.284) | 0.052 | - | - | - | - | BCC | Weak |
| *OGG1* | rs1052133 (C/G) | Good | Asian | 1 | 151/84 | Homozygous | 0.980 (0.477-2.016) | 0.957 | - | - | - | - | CCC | Weak |
|  |  |  |  | 1 | 151/84 | Heterozygous | 1.661 (0.804-3.431) | 0.170 | - | - | - | - | CCC | Weak |
|  |  |  |  | 1 | 151/84 | Dominant | 1.284 (0.667-2.473) | 0.455 | - | - | - | - | CCC | Weak |
|  |  |  |  | 1 | 151/84 | Recessive | 0.700 (0.405-1.210) | 0.202 | - | - | - | - | CCC | Weak |
|  |  |  |  | 1 | 151/84 | Allele | 0.896 (0.611-1.315) | 0.576 | - | - | - | - | BCC | Weak |
| *OPN* | T66G | Good | Asian | 1 | 167/180 | Homozygous | 0.918 (0.562-1.502) | 0.734 | - | - | - | - | BCC | Weak |
|  |  |  |  | 1 | 167/180 | Heterozygous | 0.980 (0.626-1.533) | 0.928 | - | - | - | - | BCC | Weak |
|  |  |  |  | 1 | 167/180 | Dominant | 0.955 (0.634-1.439) | 0.825 | - | - | - | - | BCC | Weak |
|  |  |  |  | 1 | 167/180 | Recessive | 0.930 (0.615-1.406) | 0.730 | - | - | - | - | BCC | Weak |
|  |  |  |  | 1 | 167/180 | Allele | 0.953 (0.732-1.240) | 0.718 | - | - | - | - | BCC | Weak |
| *P53* | rs1042522 (C/G) | Good | Asian/ | 2 | 465/138 | Recessive | 0.926 (0.619-1.383) | 0.706 | 0.0 | 0.3280 | 0.317 | - | BAB | Moderate |
|  |  |  | European | 2 | 465/138 | Allele | 0.977 (0.733-1.302) | 0.873 | 0.0 | 0.4650 | 0.317 | - | BAB | Moderate |
|  |  |  |  | 2 | 465/138 | Homozygous | 1.076 (0.576-2.010) | 0.818 | 0.0 | 0.9160 | 0.317 | - | CAB | Weak |
|  |  |  |  | 2 | 465/138 | Heterozygous | 1.070 (0.590-1.938) | 0.824 | 0.0 | 0.6200 | 0.317 | - | CAB | Weak |
|  |  |  |  | 2 | 465/138 | Dominant | 1.224 (0.696-2.152) | 0.482 | 33.3 | 0.2210 | 0.317 | - | CBB | Weak |
| *PARP1* | rs1136410 (T/C) | Good | Asian | 2 | 191/104 | Homozygous | 0.717 (0.071-7.269) | 0.778 | 88.7 | 0.0300 | 0.317 | - | CCB | Weak |
|  |  |  |  | 2 | 191/104 | Heterozygous | 0.790 (0.264-2.362)72.9 | 0.673 | 72.9 | 0.0550 | 0.317 | - | CCB | Weak |
|  |  |  |  | 2 | 191/104 | Dominant | 0.753 (0.188-3.017) | 0.689 | 84.5 | 0.0110 | 0.317 | - | CCB | Weak |
|  |  |  |  | 2 | 191/104 | Recessive | 0.842 (0.171-4.150) | 0.833 | 81.9 | 0.0190 | 0.317 | - | CCB | Weak |
|  |  |  |  | 2 | 191/104 | Allele | 0.838 (0.301-2.334) | 0.735 | 88.7 | 0.0030 | 0.317 | - | BCB | Weak |
| *PD-L1* | rs2297136 (T/C) | Good | Asian | 1 | 196/180 | Homozygous | 0.309 (0.059-1.620) | 0.165 | - | - | - | - | CCC | Weak |
|  | rs4143815 (C/G) | Good | Asian | 1 | 192/174 | Dominant | 0.780 (0.508-1.197) | 0.255 | - | - | - | - | BCC | Weak |
|  |  |  |  | 1 | 192/174 | Heterozygous | 0.932 (0.571-1.470) | 0.762 | - | - | - | - | BCC | Weak |
| *PFKL* | rs2073436 (C/G) | Good | Asian | 1 | 187/172 | Heterozygous | 1.424 (0.920-2.204) | 0.113 | - | - | - | - | CCC | Weak |
| *POLK* | rs1018119 (G/A) | Good | Asian | 1 | 537/120 | Dominant | 0.735 (0.447-1.207) | 0.224 | - | - | - | - | BCC | Weak |
|  | rs4604177 (G/A) | Good | Asian | 1 | 538/120 | Recessive | 0.774 (0.498-1.202) | 0.254 | - | - | - | - | BCC | Weak |
|  | rs10077427 (A/G) | Good | Asian | 1 | 533/117 | Dominant | 1.030 (0.679-1.563) | 0.889 | - | - | - | - | BCC | Weak |
|  | rs5744545 (G/A) | Good | Asian | 1 | 538/117 | Recessive | 1.052 (0.679-1.630) | 0.822 | - | - | - | - | BCC | Weak |
|  | rs3756558 (A/G) | Good | Asian | 1 | 538/120 | Dominant | 1.082 (0.715-1.636) | 0.711 | - | - | - | - | BCC | Weak |
|  | rs449106 (A/G) | Good | Asian | 1 | 538/120 | Dominant | 1.064 (0.483-2.343) | 0.878 | - | - | - | - | CCC | Weak |
|  | rs5744653 (G/A) | Good | Asian | 1 | 535/119 | Dominant | 0.958 (0.592-1.551) | 0.861 | - | - | - | - | BCC | Weak |
|  | rs5744651 (G/A) | Good | Asian | 1 | 538/120 | Dominant | 0.996 (0.547-1.812) | 0.989 | - | - | - | - | CCC | Weak |
|  | rs5744655 (A/G) | Good | Asian | 1 | 538/120 | Dominant | 0.513 (0.254-1.037) | 0.063 | - | - | - | - | CCC | Weak |
| *PTEN* | rs11202607 (G/A) | Good | Asian | 1 | 376/100 | Homozygous | 1.304 (0.150-1.327) | 0.810 | - | - | - | - | CCC | Weak |
|  |  |  |  | 1 | 376/100 | Heterozygous | 0.939 (0.539-1.635) | 0.824 | - | - | - | - | BCC | Weak |
|  |  |  |  | 1 | 376/100 | Dominant | 0.957 (0.556-1.646) | 0.873 | - | - | - | - | CCC | Weak |
|  |  |  |  | 1 | 376/100 | Recessive | 1.321 (0.153-11.436) | 0.801 | - | - | - | - | CCC | Weak |
|  |  |  |  | 1 | 376/100 | Allele | 0.979 (0.594-1.613) | 0.934 | - | - | - | - | BCC | Weak |
|  | rs701848 (G/A) | Good | Asian | 1 | 111/100 | Heterozygous | 1.475 (0.925-2.353) | 0.103 | - | - | - | - | BCC | Weak |
|  |  |  |  | 1 | 111/100 | Recessive | 1.567 (0.871-2.818) | 0.134 | - | - | - | - | CCC | Weak |
|  | rs11202592 (G/C) | Good | Asian | 1 | 364/111 | Homozygous | 0.859 (0.302-0.445) | 0.776 | - | - | - | - | CCC | Weak |
|  |  |  |  | 1 | 364/111 | Heterozygous | 1.135 (0.544-2.367) | 0.736 | - | - | - | - | CCC | Weak |
|  |  |  |  | 1 | 364/111 | Dominant | 1.043 (0.561-1.937) | 0.894 | - | - | - | - | BCC | Weak |
|  |  |  |  | 1 | 364/111 | Recessive | 0.848 (0.299-2.409) | 0.757 | - | - | - | - | CCC | Weak |
|  |  |  |  | 1 | 364/111 | Allele | 0.990 (0.586-1.674) | 0.971 | - | - | - | - | BCC | Weak |
| *PXR* | rs3814058 (T/C) | Good | Asian | 1 | 149/113 | Homozygous | 1.090 (0.537-2.213) | 0.811 | - | - | - | - | CCC | Weak |
|  |  |  |  | 1 | 149/113 | Heterozygous | 1.297 (0.718-2.342) | 0.388 | - | - | - | - | CCC | Weak |
|  |  |  |  | 1 | 149/113 | Dominant | 1.231 (0.703-2.157) | 0.467 | - | - | - | - | CCC | Weak |
|  |  |  |  | 1 | 149/113 | Recessive | 0.915 (0.509-1.646) | 0.767 | - | - | - | - | CCC | Weak |
|  |  |  |  | 1 | 149/113 | Allele | 1.049 (0.743-1.483) | 0.784 | - | - | - | - | BCC | Weak |
| *RAD51* | rs180132 (G/C) | Good | Asian | 1 | 77/62 | Dominant | 0.758 (0.337-1.704) | 0.503 | - | - | - | - | CCC | Weak |
| *RAGE* | rs1800624 (T/A) | Good | Asian | 1 | 133/274 | Homozygous | 1.915 (0.946-3.875) | 0.071 | - | - | - | - | CCC | Weak |
|  |  |  |  | 1 | 133/274 | Heterozygous | 1.628 (0.968-2.736) | 0.066 | - | - | - | - | CCC | Weak |
|  |  |  |  | 1 | 133/274 | Recessive | 1.323 (0.735-2.381) | 0.351 | - | - | - | - | CCC | Weak |
|  |  |  |  | 1 | 133/274 | Allele | 1.273 (0.948-1.708) | 0.108 | - | - | - | - | BCC | Weak |
|  | rs2070600 (G>T/A) | Good | Asian | 1 | 274/133 | Recessive | 1.355 (0.770-2.315) | 0.303 | - | - | - | - | CCC | Weak |
|  | rs1800625 (T/C) | Good | Asian | 1 | 133/274 | Homozygous | 1.179 (0.589-2.358) | 0.642 | - | - | - | - | CCC | Weak |
|  |  |  |  | 1 | 133/274 | Heterozygous | 0.869 (0.503-1.502) | 0.614 | - | - | - | - | CCC | Weak |
|  |  |  |  | 1 | 133/274 | Dominant | 1.407 (0.824-2.403) | 0.211 | - | - | - | - | CCC | Weak |
|  |  |  |  | 1 | 133/274 | Recessive | 0.869 (0.503-1.502) | 0.614 | - | - | - | - | CCC | Weak |
|  |  |  |  | 1 | 133/274 | Allele | 1.062 (0.792-1.423) | 0.690 | - | - | - | - | BCC | Weak |
| *REV3* | rs240969 (G/A) | Good | Asian | 1 | 536/119 | Homozygous | 1.788 (0.912-3.508) | 0.091 | - | - | - | - | CCC | Weak |
|  |  |  |  | 1 | 536/119 | Recessive | 1.384 (0.725-2.640) | 0.325 | - | - | - | - | CCC | Weak |
|  | rs456865 (G/A) | Good | Asian | 1 | 532/119 | Homozygous | 1.750 (0.992-3.090) | 0.053 | - | - | - | - | BCC | Weak |
|  |  |  |  | 1 | 532/119 | Heterozygous | 1.163 (0.697-1.943) | 0.563 | - | - | - | - | BCC | Weak |
|  |  |  |  | 1 | 532/119 | Dominant | 1.367 (0.843-2.217) | 0.204 | - | - | - | - | BCC | Weak |
|  | rs45945880 (G/A) | Good | Asian | 1 | 538/120 | Homozygous | 1.731 (0.816-3.671) | 0.153 | - | - | - | - | CCC | Weak |
|  |  |  |  | 1 | 538/120 | Heterozygous | 1.343 (0.883-2.043) | 0.167 | - | - | - | - | BCC | Weak |
|  |  |  |  | 1 | 538/120 | Dominant | 1.408 (0.946-2.095) | 0.092 | - | - | - | - | BCC | Weak |
|  |  |  |  | 1 | 538/120 | Recessive | 1.519 (0.731-3.156) | 0.262 | - | - | - | - | CCC | Weak |
|  |  |  |  | 1 | 538/120 | Allele | 1.337 (0.976-1.832) | 0.070 | - | - | - | - | BCC | Weak |
|  | rs3218573 (G/A) | Good | Asian | 1 | 105/31 | Homozygous | 1.619 (0.322-8.150) | 0.559 | - | - | - | - | CCC | Weak |
|  |  |  |  | 1 | 105/31 | Heterozygous | 1.111 (0.212-5.827) | 0.901 | - | - | - | - | CCC | Weak |
|  |  |  |  | 1 | 105/31 | Dominant | 1.503 (0.300-7.539) | 0.621 | - | - | - | - | CCC | Weak |
|  |  |  |  | 1 | 105/31 | Recessive | 1.467 (0.915-2.354) | 0.112 | - | - | - | - | BCC | Weak |
|  |  |  |  | 1 | 105/31 | Allele | 1.416 (0.922-2.175) | 0.112 | - | - | - | - | BCC | Weak |
| *REV7* | rs23336030 (G/A) | Good | Asian | 1 | 537/120 | Homozygous | 0.536 (0.292-0.983) | 0.044 | - | - | - | - | BCC | Weak |
|  |  |  |  | 1 | 537/121 | Heterozygous | 0.636 (0.355-1.140) | 0.129 | - | - | - | - | BCC | Weak |
|  |  |  |  | 1 | 537/122 | Dominant | 0.592 (0.341-1.029) | 0.063 | - | - | - | - | BCC | Weak |
|  |  |  |  | 1 | 537/123 | Recessive | 0.746 (0.494-1.126) | 0.163 | - | - | - | - | BCC | Weak |
| *RRM1* | rs12806698 (C/A) | Good | Asian/ | 5 | 277/208 | Heterozygous | 1.103 (0.569-2.135) | 0.772 | 56.2 | 0.0580 | 1.000 | 0.945 | BBA | Moderate |
|  |  |  | European | 5 | 277/208 | Dominant | 1.171 (0.816-1.680) | 0.392 | 30.9 | 0.2040 | 0.851 | 0.938 | BBA | Moderate |
|  |  |  |  | 5 | 277/208 | Allele | 1.118 (0.824-1.516) | 0.474 | 0.0 | 0.6660 | 0.624 | 0.924 | BAA | Moderate |
|  | rs11030813 (C/T) | Good | Asian/European | 2 | 234/148 | Recessive | 1.079 (0.711-1.637) | 0.721 | 0.0 | 0.8650 | 0.317 | - | BAB | Moderate |
|  | rs12806698 (C/A) | Good | Asian/ | 5 | 277/208 | Homozygous | 1.543 (0.522-4.554) | 0.433 | 0.0 | 0.8490 | 0.117 | 0.334 | CAA | Weak |
|  |  |  | European | 5 | 277/208 | Recessive | 1.865 (0.657-5.291) | 0.242 | 0.0 | 0.9930 | 0.117 | 0.467 | CAA | Weak |
|  | rs9937 (A/G) | Good | Asian | 2 | 90/28 | Homozygous | 1.007 (0.223-4.353) | 0.992 | 0.0 | 0.8950 | 0.317 | - | CAB | Weak |
|  |  |  |  | 2 | 90/28 | Heterozygous | 0.646 (0.215-1.943) | 0.436 | 25.1 | 0.2480 | 0.317 | - | CAB | Weak |
|  |  |  |  | 2 | 90/28 | Dominant | 0.710 (0.261-1.929) | 0.502 | 0.0 | 0.3180 | 0.317 | - | CAB | Weak |
|  |  |  |  | 2 | 90/28 | Recessive | 1.233 (0.315-4.829) | 0.763 | 0.0 | 0.4650 | 0.317 | - | CAB | Weak |
|  |  |  |  | 2 | 90/28 | Allele | 0.907 (0.485-1.694) | 0.759 | 0.0 | 0.7700 | 0.317 | - | CAB | Weak |
|  | rs1042858 (G/A) | Good | Asian | 2 | 90/28 | Recessive | 0.869 (0.360-2.101) | 0.755 | 0.0 | 0.4920 | 0.317 | - | CAB | Weak |
|  |  |  |  | 2 | 90/28 | Allele | 0.694 (0.343-1.402) | 0.309 | 0.0 | 3.9000 | 0.317 | - | CAB | Weak |
|  | rs11030813 (C/T) | Good | Asian/ | 2 | 234/148 | Homozygous | 0.490 (0.218-1.101) | 0.084 | 0.0 | 0.5970 | 0.317 | - | CAB | Weak |
|  |  |  | European | 2 | 234/148 | Allele | 0.889 (0.648-1.221) | 0.468 | 0.0 | 0.7430 | 0.317 | - | CAB | Weak |
| *SLC19A1* | C2522T | Good | Asian | 1 | 30/15 | Homozygous | 1.000 (0.153-6.531) | 1.000 | - | - | - | - | CCC | Weak |
|  |  |  |  | 1 | 30/15 | Heterozygous | 0.583 (0.121-2.801) | 0.501 | - | - | - | - | CCC | Weak |
|  |  |  |  | 1 | 30/15 | Dominant | 0.687 (0.153-3.087) | 0.625 | - | - | - | - | CCC | Weak |
|  |  |  |  | 1 | 30/15 | Recessive | 1.455 (0.324-6.531) | 0.625 | - | - | - | - | CCC | Weak |
|  |  |  |  | 1 | 30/15 | Allele | 1.000 (0.416-2.403) | 1.000 | - | - | - | - | CCC | Weak |
| *STMN1* | T2166C | Good | European | 1 | 126/64 | Homozygous | 0.211 (0.039-1.156) | 0.073 | - | - | - | - | CCC | Weak |
|  |  |  |  | 1 | 126/64 | Heterozygous | 0.215 (0.039-1.173) | 0.076 | - | - | - | - | CCC | Weak |
|  |  |  |  | 1 | 126/64 | Dominant | 0.213 (0.041-1.110) | 0.066 | - | - | - | - | CCC | Weak |
|  |  |  |  | 1 | 126/64 | Recessive | 0.804 (0.372-1.738) | 0.579 | - | - | - | - | CCC | Weak |
|  |  |  |  | 1 | 126/64 | Allele | 0.697 (0.390-1.247) | 0.224 | - | - | - | - | CCC | Weak |
| *TAQ1* | rs10735810 (T/C) | Good | Asian | 1 | 434/321 | Homozygous | 0.725 (0.488-1.079) | 0.113 | - | - | - | - | BCC | Weak |
|  |  |  |  | 1 | 434/321 | Heterozygous | 0.792 (0.563-1.115) | 0.182 | - | - | - | - | BCC | Weak |
|  |  |  |  | 1 | 434/321 | Dominant | 0.769 (0.559-1.057) | 0.105 | - | - | - | - | BCC | Weak |
|  |  |  |  | 1 | 434/321 | Recessive | 0.836 (0.598-1.171) | 0.298 | - | - | - | - | BCC | Weak |
|  |  |  |  | 1 | 434/321 | Allele | 0.839 (0.684-1.099) | 0.093 | - | - | - | - | BCC | Weak |
| *TMEM205* | rs896412 (G/C) | Good | Asian | 1 | 153/138 | Heterozygous | 1.042 (0.642-1.632) | 0.922 | - | - | - | - | BCC | Weak |
|  |  |  |  | 1 | 153/138 | Dominant | 1.218 (0.775-1.914) | 0.392 | - | - | - | - | BCC | Weak |
|  |  |  |  | 1 | 153/138 | Allele | 1.399 (0.947-2.065) | 0.092 | - | - | - | - | BCC | Weak |
| *TNFRSF1B* | rs1061624 (A/G) | Good | Asian | 1 | 187/173 | Homozygous | 1.671 (0.914-3.055) | 0.095 | - | - | - | - | CCC | Weak |
|  |  |  |  | 1 | 187/173 | Heterozygous | 1.257 (0.752-2.101) | 0.382 | - | - | - | - | CCC | Weak |
|  |  |  |  | 1 | 187/173 | Dominant | 1.319 (0.809-2.151) | 0.268 | - | - | - | - | CCC | Weak |
|  |  |  |  | 1 | 187/173 | Recessive | 1.427 (0.876-2.325) | 0.153 | - | - | - | - | CCC | Weak |
|  |  |  |  | 1 | 187/173 | Allele | 1.277 (0.952-1.711) | 0.102 | - | - | - | - | BCC | Weak |
| *TP53* | rs1042522 (A/C) | Good | Asian | 1 | 409/231 | Heterozygous | 0.705 (0.493-1.008) | 0.055 | - | - | - | - | BCC | Weak |
| *TS* | 2R/3R | Good | European | 1 | 20/95 | Homozygous | 2.667 (0.439-16.198) | 0.287 | - | - | - | - | CCC | Weak |
|  |  |  |  | 1 | 20/95 | Heterozygous | 3.170 (0.667-15.056) | 0.147 | - | - | - | - | CCC | Weak |
|  |  |  |  | 1 | 20/95 | Dominant | 3.429 (0.751-15.650) | 0.112 | - | - | - | - | CCC | Weak |
|  |  |  |  | 1 | 20/95 | Recessive | 0.556 (0.163-1.898) | 0.348 | - | - | - | - | CCC | Weak |
|  |  |  |  | 1 | 20/95 | Allele | 1.739 (0.870-3.477) | 0.118 | - | - | - | - | CCC | Weak |
| *XPA* | rs1800975 (A/G) | Good | Asian/ | 2 | 157/119 | Recessive | 0.737 (0.445-1.221) | 0.236 | 0.0 | 0.7510 | 0.317 | - | BAB | Moderate |
|  |  |  | European | 2 | 157/119 | Allele | 0.704 (0.385-1.388) | 0.255 | 59.9 | 0.1140 | 0.317 | - | BBB | Moderate |
|  |  |  |  | 2 | 157/119 | Homozygous | 0.603 (0.135-2.798) | 0.508 | 67.2 | 0.8100 | 0.317 | - | CBB | Weak |
|  |  |  |  | 2 | 157/119 | Heterozygous | 0.480 (0.035-6.480) | 0.580 | 89.1 | 0.0020 | 0.317 | - | CCB | Weak |
|  |  |  |  | 2 | 157/119 | Dominant | 0.535 (0.070-4.068) | 0.546 | 84.3 | 0.0120 | 0.317 | - | CCB | Weak |
| *XPA* | A4G | Good | European | 1 | 40/50 | Homozygous | 0.855 (0.192-3.803) | 0.837 | - | - | - | - | CCC | Weak |
|  |  |  |  | 1 | 40/50 | Heterozygous | 1.198 (0.286-5.025) | 0.805 | - | - | - | - | CCC | Weak |
|  |  |  |  | 1 | 40/50 | Dominant | 1.047 (0.261-4.190) | 0.949 | - | - | - | - | CCC | Weak |
|  |  |  |  | 1 | 40/50 | Recessive | 0.735 (0.305-1.770) | 0.492 | - | - | - | - | CCC | Weak |
|  |  |  |  | 1 | 40/50 | Allele | 0.867 (0.469-1.602) | 0.648 | - | - | - | - | CCC | Weak |
| *XPC* | rs2228001 (A/C) | Good | Asian/ | 4 | 266/227 | Homozygous | 0.918 (0.415-2.031) | 0.833 | 42.8 | 0.1550 | 1.000 | 0.902 | BBA | Moderate |
|  |  |  | European | 4 | 266/227 | Heterozygous | 1.516 (0.700-3.283) | 0.291 | 63.9 | 0.0400 | 0.497 | 0.988 | BBA | Moderate |
|  |  |  |  | 4 | 266/227 | Dominant | 1.376 (0.664-2.849) | 0.390 | 65.1 | 0.0350 | 1.000 | 0.942 | BBA | Moderate |
|  |  |  |  | 4 | 266/227 | Recessive | 0.813 (0.464-1.423) | 0.468 | 23.6 | 0.2690 | 0.497 | 0.912 | BBA | Moderate |
|  |  |  |  | 4 | 266/227 | Allele | 1.107 (0.709-1.729) | 0.655 | 60.1 | 0.0570 | 1.000 | 0.673 | BBA | Moderate |
|  | rs228000 (C/T) | Good | Asian/ | 1 | 190/163 | Heterozygous | 0.722 (0.437-1.193) | 0.203 | 20.4 | 0.2850 | 0.602 | 0.149 | BAA | Moderate |
|  |  |  | European | 1 | 190/163 | Dominant | 0.719 (0.440-1.172) | 0.186 | 21.1 | 0.2810 | 0.117 | 0.159 | BAA | Moderate |
|  |  |  |  | 1 | 190/163 | Allele | 0.808 (0.590-1.107) | 0.185 | 0.0 | 0.4420 | 0.602 | 0.552 | BAA | Moderate |
|  | C11A | Good | Asian | 1 | 107/57 | Homozygous | 0.765 (0.322-1.815) | 0.543 | - | - | - | - | CCC | Weak |
|  |  |  |  | 1 | 107/57 | Heterozygous | 0.552 (0.247-1.236) | 0.149 | - | - | - | - | CCC | Weak |
|  |  |  |  | 1 | 107/57 | Dominant | 0.634 (0.302-1.330) | 0.228 | - | - | - | - | CCC | Weak |
|  |  |  |  | 1 | 107/57 | Recessive | 1.096 (0.545-2.203) | 0.797 | - | - | - | - | CCC | Weak |
|  |  |  |  | 1 | 107/57 | Allele | 0.869 (0.551-1.370) | 0.545 | - | - | - | - | BCC | Weak |
|  | rs228000 (C/T) | Good | Asian/European | 1 | 190/163 | Homozygous | 0.543 (0.205-1.439) | 0.219 | 0.0 | 0.6670 | 0.602 | 0.833 | CAA | Weak |
|  |  |  |  | 1 | 190/163 | Recessive | 0.713 (0.283-1.795) | 0.479 | 0.0 | 0.7900 | 0.602 | 0.996 | CAA | Weak |
| *XPC PAT* | rs77907221 (S/L) | Good | Asian | 2 | 226/125 | Dominant | 0.723 (0.460-1.136) | 0.159 | 0.0 | 0.9660 | 0.317 | - | BAB | Moderate |
| *XRCC1* | rs25487 (G/A) | Good | Asian | 19 | 2948/2035 | Heterozygous | 0.989 (0.785-1.246) | 0.925 | 65.5 | 0.0001 | 0.030 | 0.030 | ABB | Moderate |
|  |  |  |  | 24 | 3304/2199 | Dominant | 0.991 (0.788-1.245) | 0.935 | 71.3 | 0.0001 | 0.083 | 0.024 | ABB | Moderate |
|  |  |  |  | 19 | 2948/2035 | Allele | 0.822 (0.732-1.062) | 0.185 | 72.8 | 0.0001 | 0.086 | 0.109 | ABA | Moderate |
|  | rs25489 (G/A) | Good | Asian | 3 | 434/478 | Homozygous model | 1.801 (0.666-4.870) | 0.247 | 67.0 | 0.0480 | 0.602 | 0.997 | BBA | Moderate |
|  |  |  |  | 3 | 434/478 | Heterozygous | 1.108 (0.764-1.606) | 0.588 | 0.0 | 0.5240 | 0.602 | 0.403 | BAA | Moderate |
|  |  |  |  | 3 | 434/478 | Recessive | 1.545 (0.733-3.256) | 0.253 | 70.6 | 0.0330 | 0.602 | 0.981 | BBA | Moderate |
|  |  |  |  | 3 | 434/478 | Allele | 1.355 (0.822-2.233) | 0.234 | 78.1 | 0.0100 | 0.117 | 0.324 | BBA | Moderate |
|  |  |  | Asian/European | 4 | 506/567 | Dominant | 0.492 (0.076-3.178) | 0.456 | 95.8 | 0.0001 | 0.497 | 0.658 | BCA | Weak |
| *XRCC2* | rs3218536 (C/T) | Good | European | 1 | 72/89 | Dominant | 0.933 (0.419-2.077) | 0.865 | - | - | - | - | CCC | Weak |
| *XRCC3* | rs861539 (C/T) | Good | Asian/European | 5 | 550/295 | Heterozygous | 0.722 (0.556-1.072) | 0.123 | 0.0 | 0.9900 | 0.142 | 0.278 | BAA | Moderate |
|  |  |  |  | 5 | 550/295 | Recessive | 0.898 (0.693-1.165) | 0.418 | 0.0 | 0.9950 | 0.624 | 0.837 | CAA | Weak |
