# Supplementary material for: Genetic Variants Associated With Response to Platinum-Based Chemotherapy in Non-Small Cell Lung Cancer Patients: A Field Synopsis and Meta‐Analysis
Source: Br J Biomed Sci. 2024 Feb 21;81:11835. doi: 10.3389/bjbs.2024.11835 (PMC10914946; doi:10.3389/bjbs.2024.11835)
Supplement: Supplementary file 5 [file DataSheet1.DOCX]

**Supplementary information**

ABCC2 rs717620 allele model


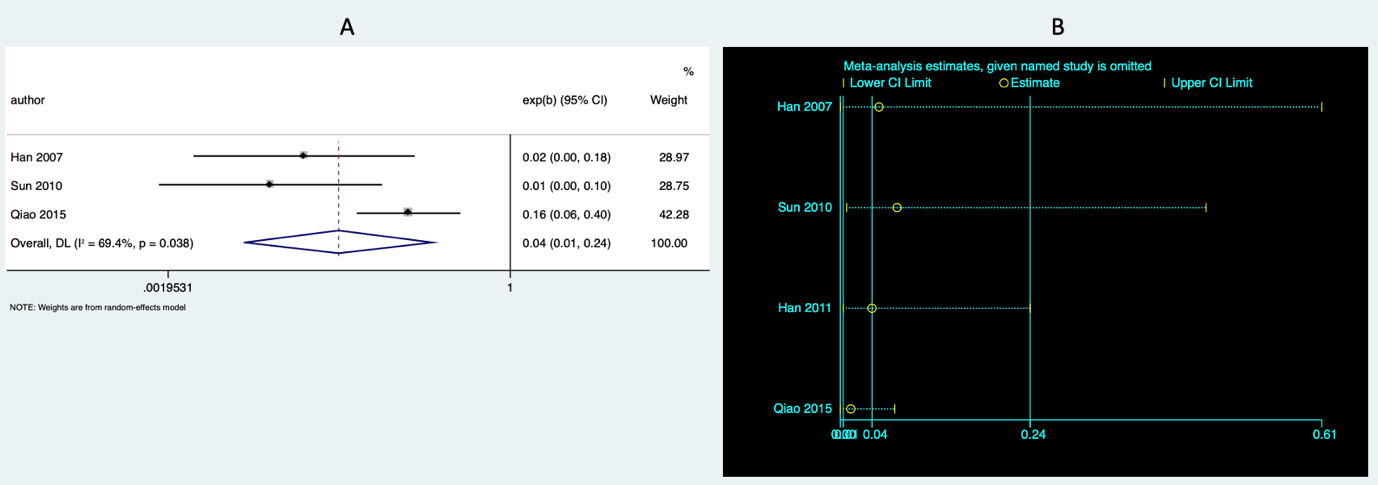


**Figure_SuppInfo_1** Forest plot (A) and the sensitivity analysis (B) of the association between *ABCC2* rs717620 and response to PBC

ABCG2 rs2231142 homozygous model


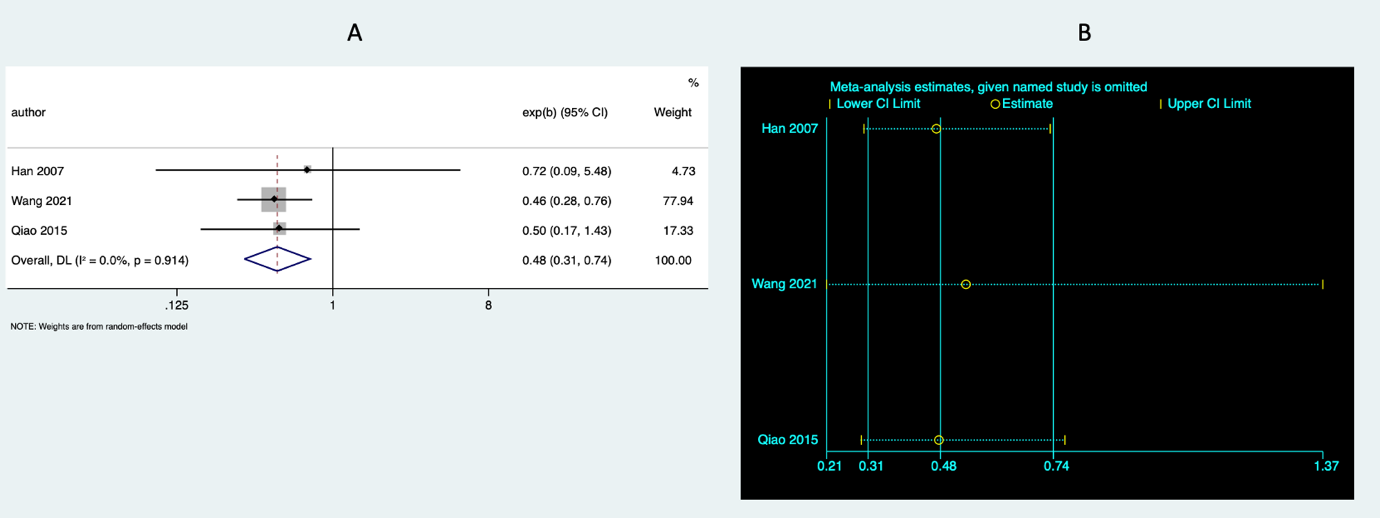


ABCG2 rs2231142 recessive model


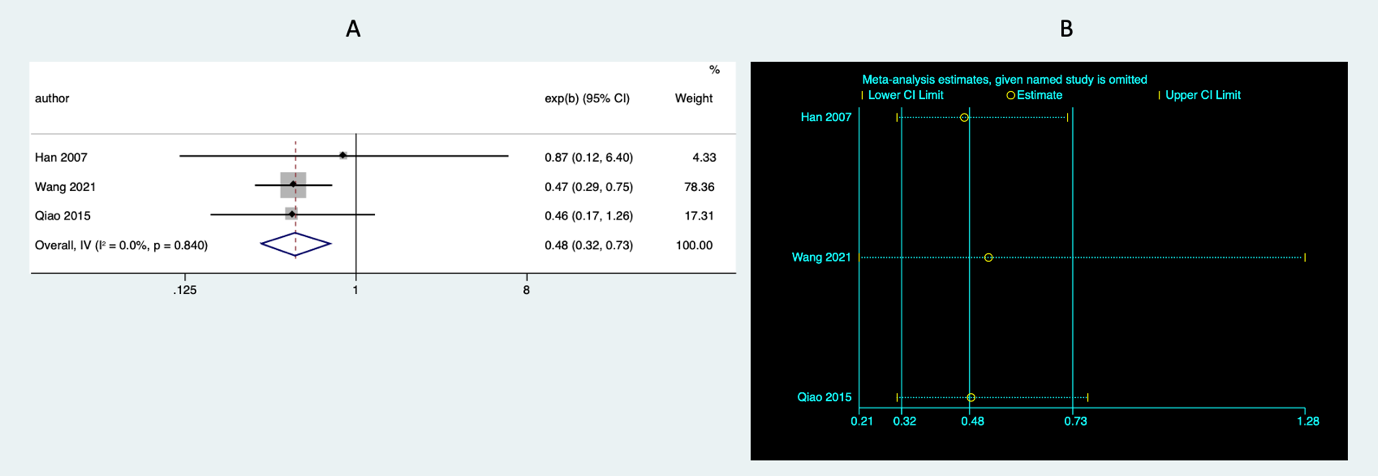


ABCG2 rs2231142 allele model


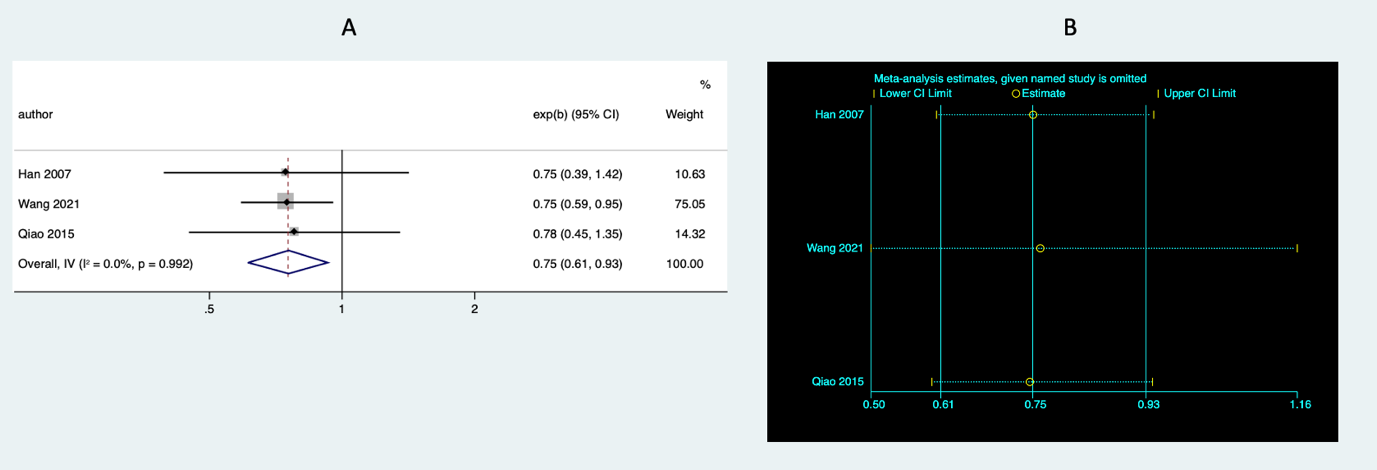


**Figure_SuppInfo_2** Forest plots (A) and the sensitivity analysis (B) of the association between *ABCG2* rs2231142 and response to PBC

*CDA* rs1048977 allele model


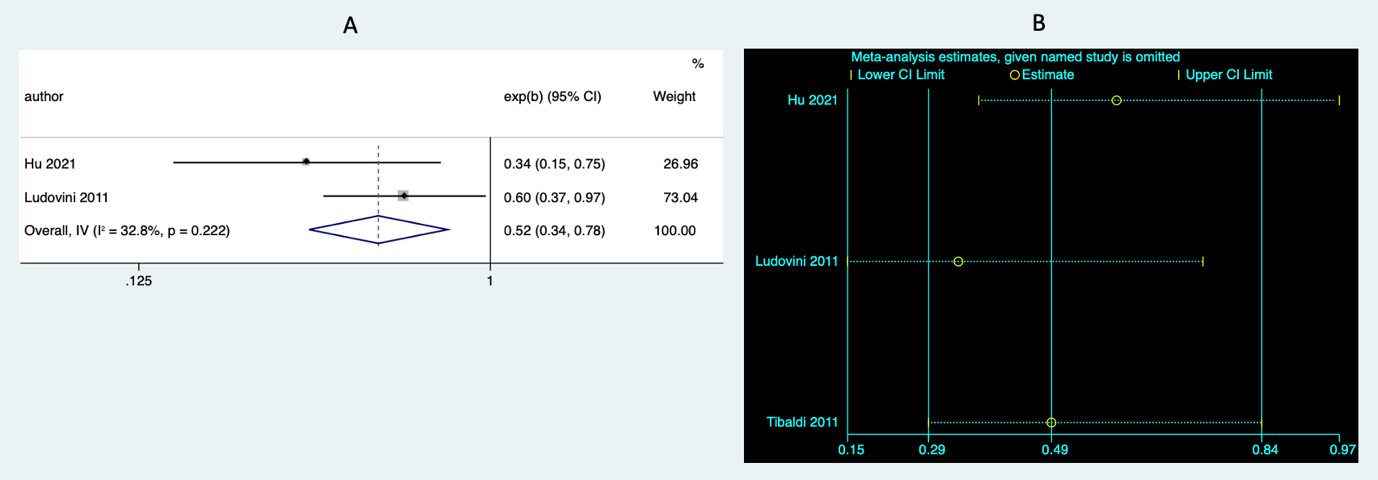


**Figure_SuppInfo_3** Forest plot (A) and the sensitivity analysis (B) of the association between *CDA* rs1048977 and response to PBC

*CYP1A1* rs1048943 heterozygous model


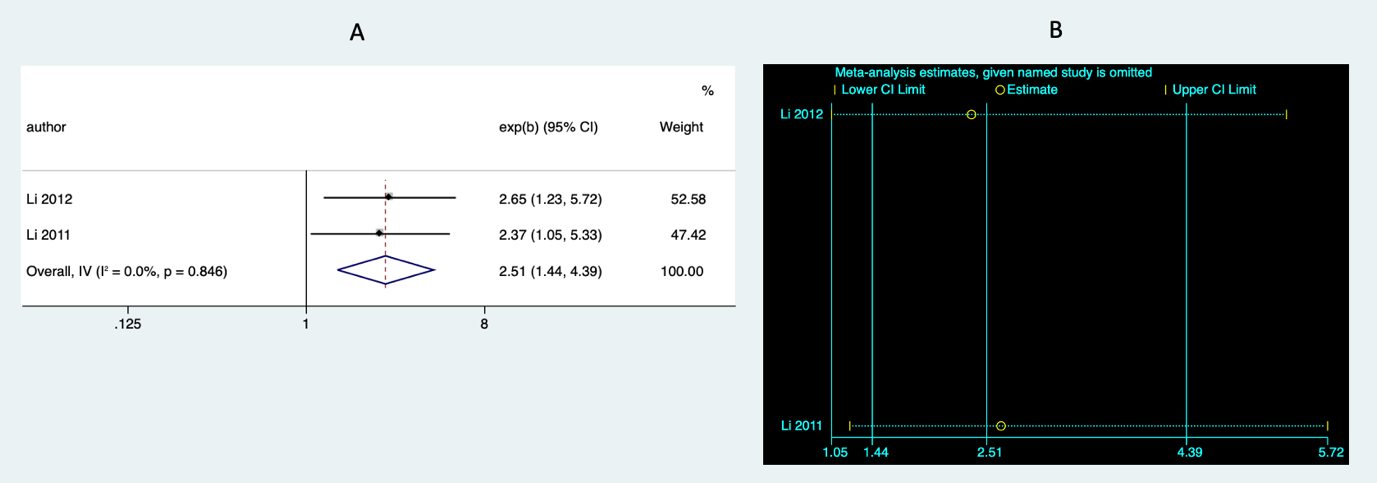


CYP1A1 rs1048943 dominant model


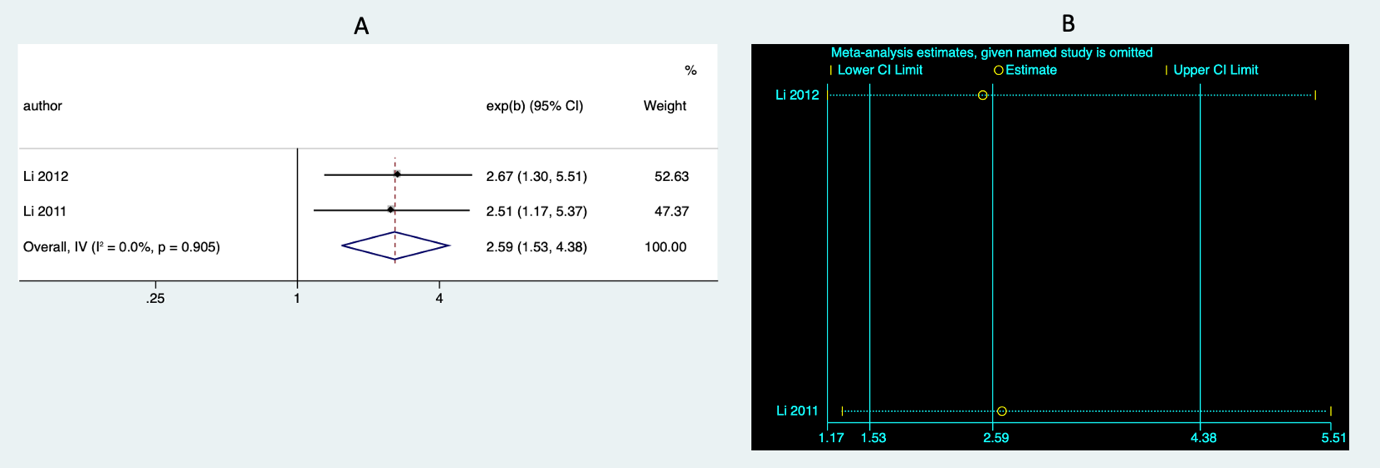


CYP1A1 rs1048943 allele model


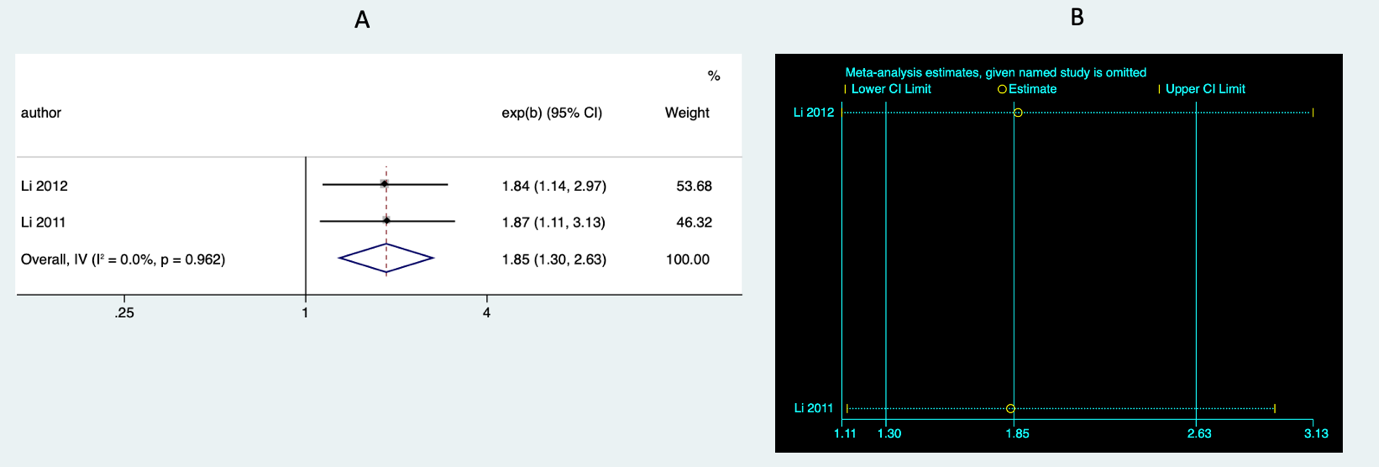


**Figure_SuppInfo_4** Forest plots (A) and the sensitivity analysis (B) of the association between *CYP1A1* rs1048943 and response to PBC

*ERCC1* rs3212986 homozygous model


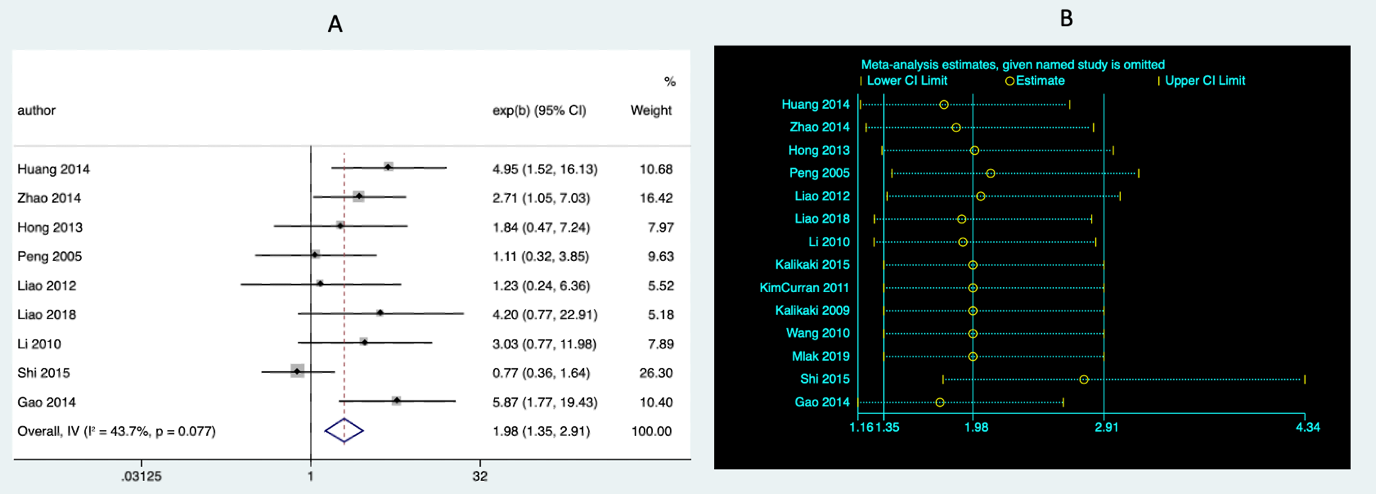


*ERCC1* rs3212986 recessive model


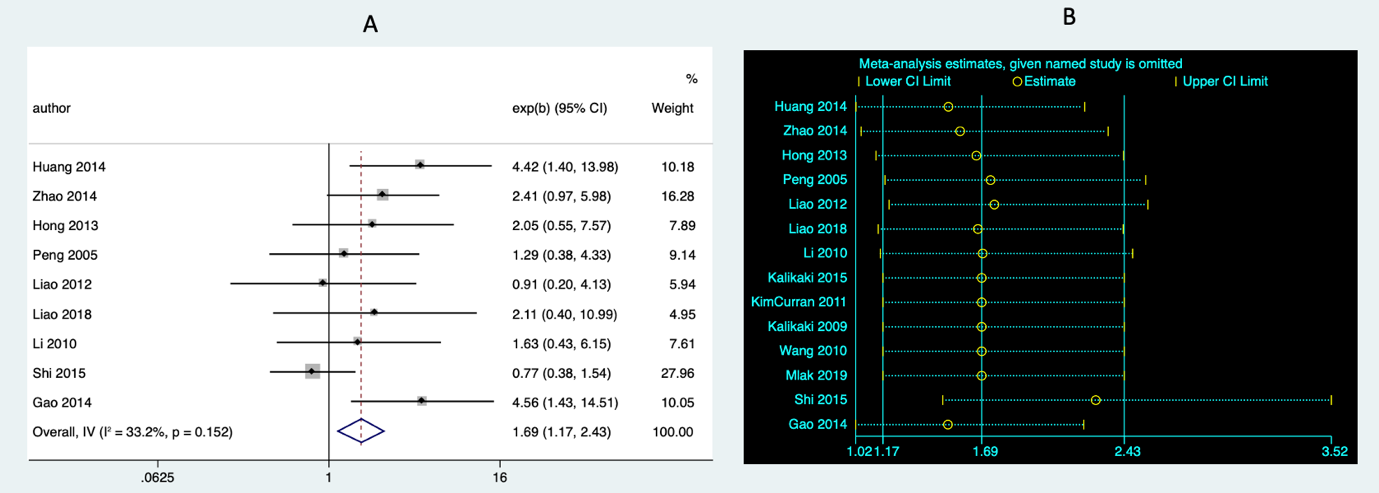


ERCC1 rs3212986 allele model


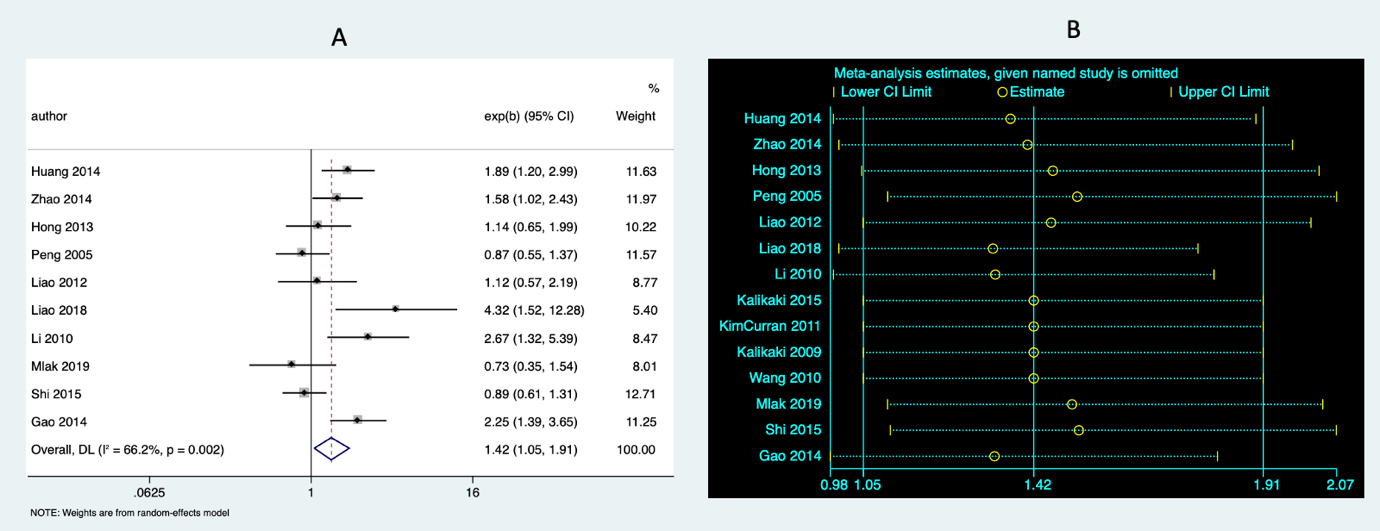


**Figure_SuppInfo_5** Forest plots (A) and the sensitivity analysis (B) of the association between *ERCC1* rs3212986 and response to PBC

ERCC2 rs1799793 dominant model


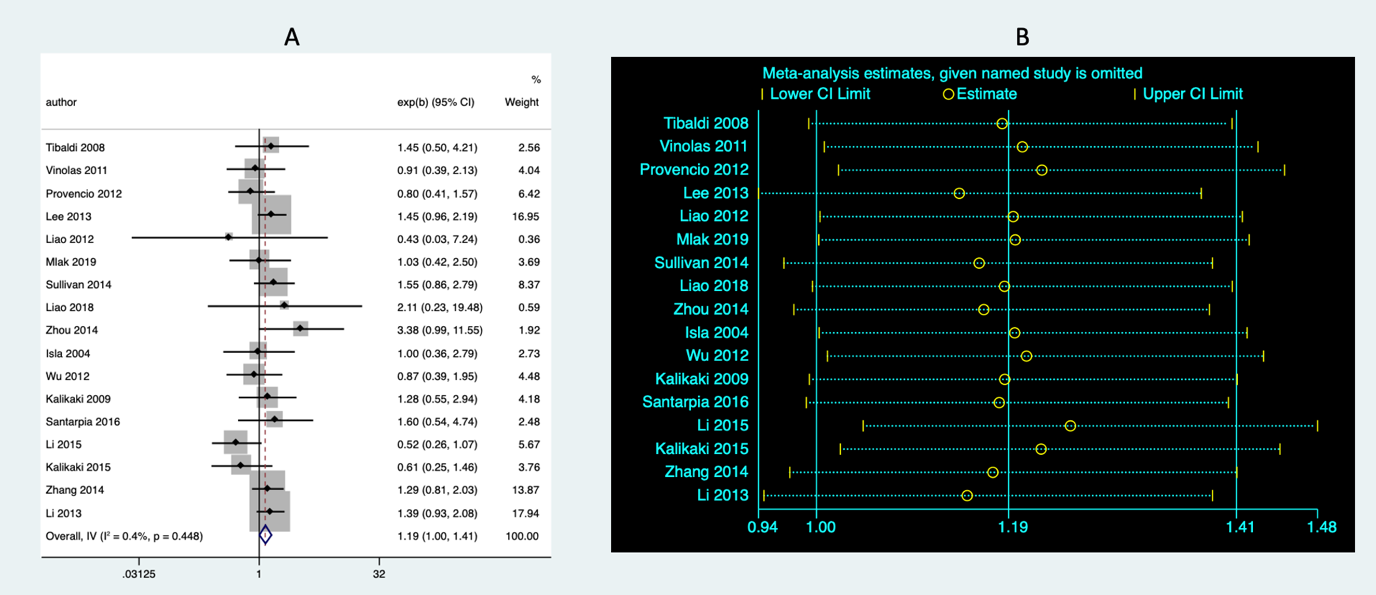


ERCC2 rs1799793 allele model

**
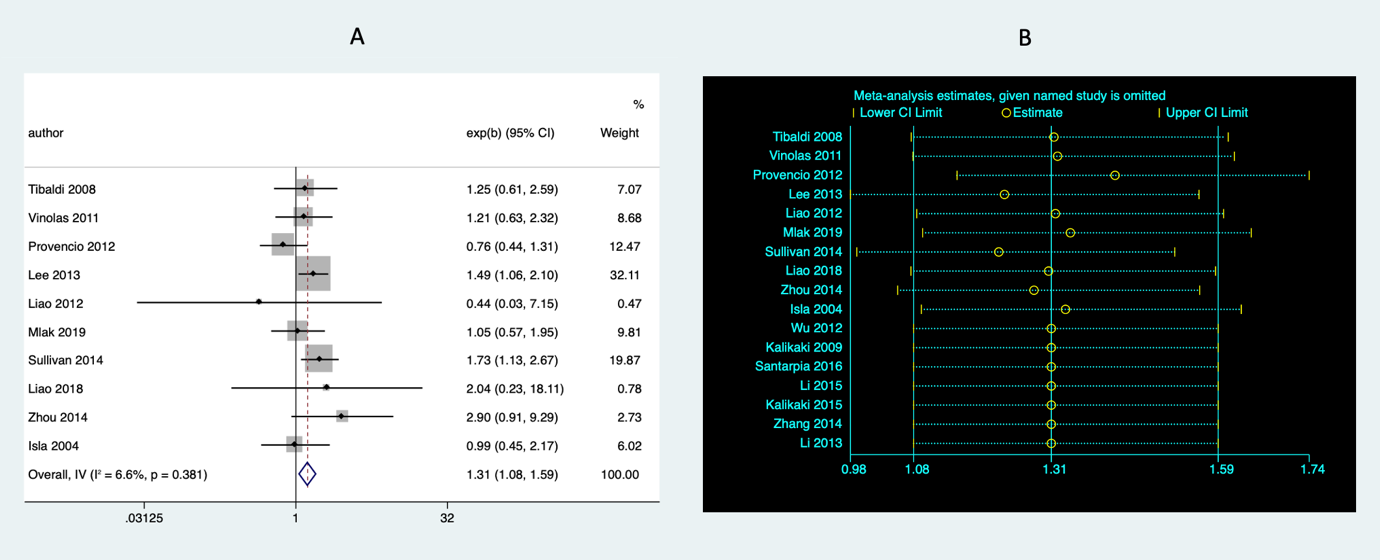
**

**Figure_SuppInfo_6** Forest plots (A) and the sensitivity analysis (B) of the association between *ERCC2* rs1799793 and response to PBC

*ERCC2* rs1052555 dominant model

**
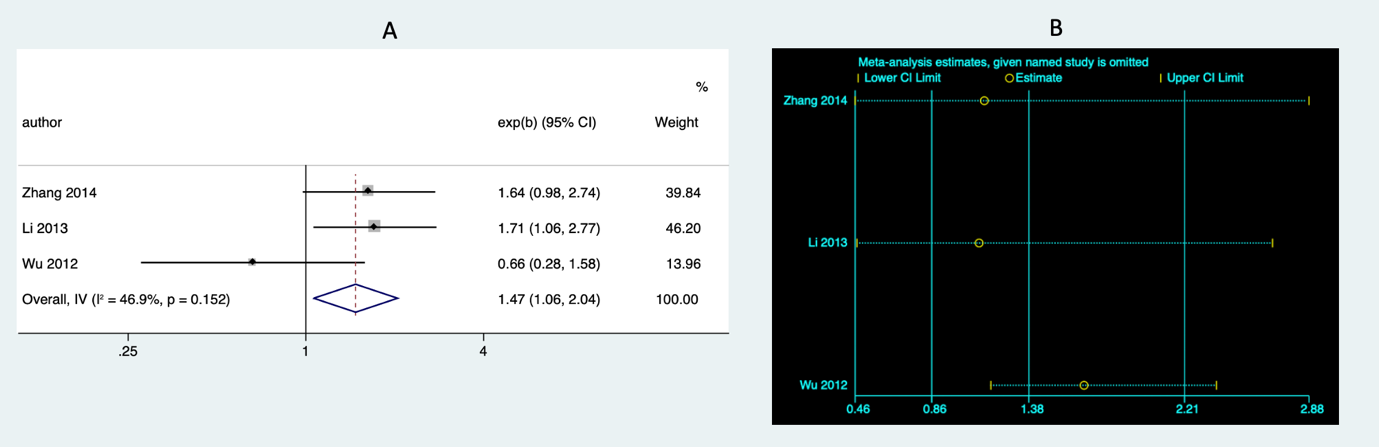
**

**Figure_SuppInfo_7** Forest plots (A) and the sensitivity analysis (B) of the association between *ERCC2* rs1052555 and response to PBC

*GSTM1 rs*36631 allele model

**
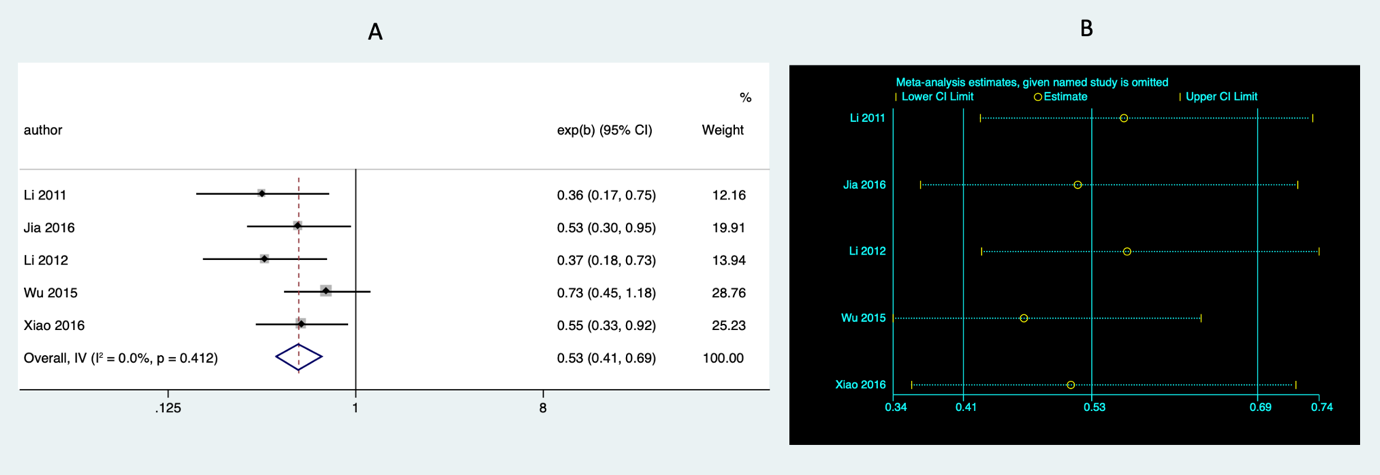
**

**Figure_SuppInfo_8** Forest plots (A) and the sensitivity analysis (B) of the association between *GSTM1 rs*36631 and response to PBC

XPC rs77907221 heterozygous model


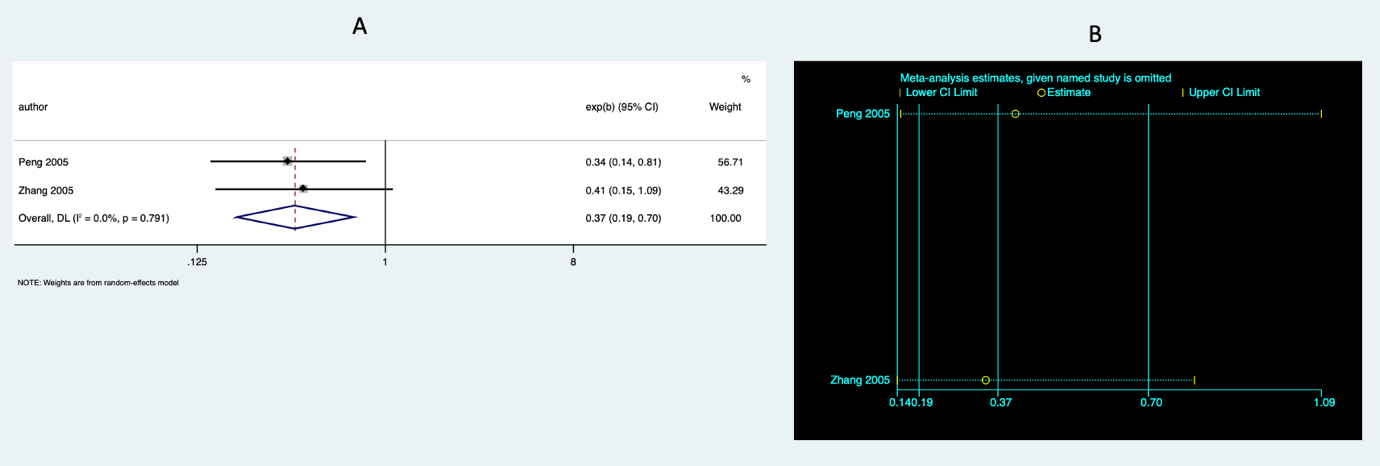


XPC rs77907221 allele model


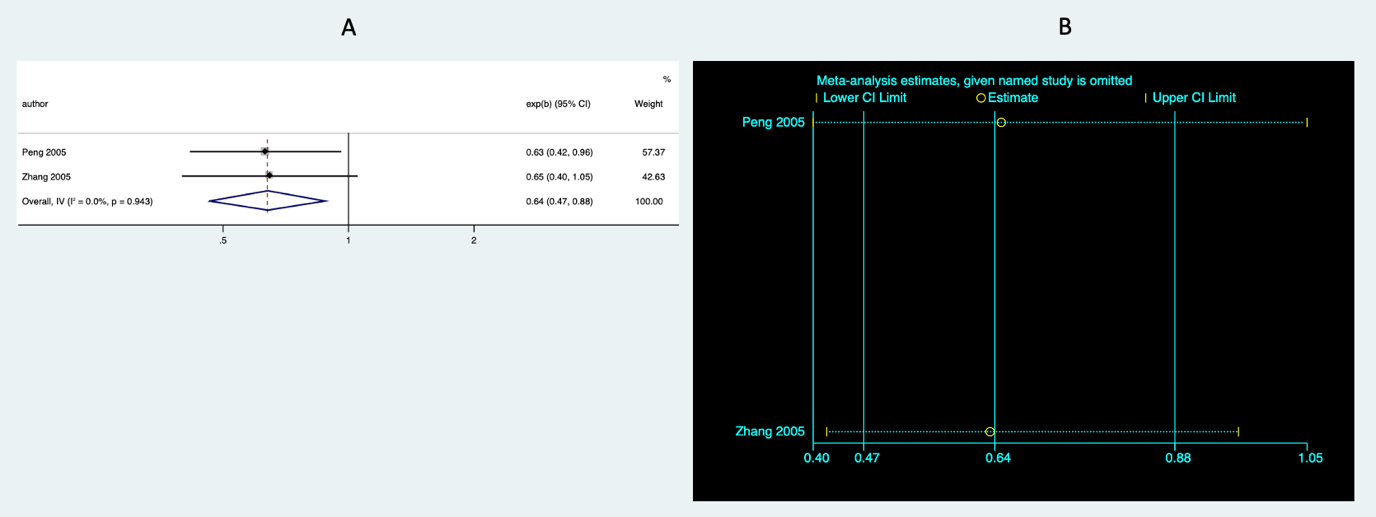


**Figure_SuppInfo_9** Forest plots (A) and the sensitivity analysis (B) of the association between *XPC* rs77907221 and response to PBC

XRCC1 rs1799782 homozygous model


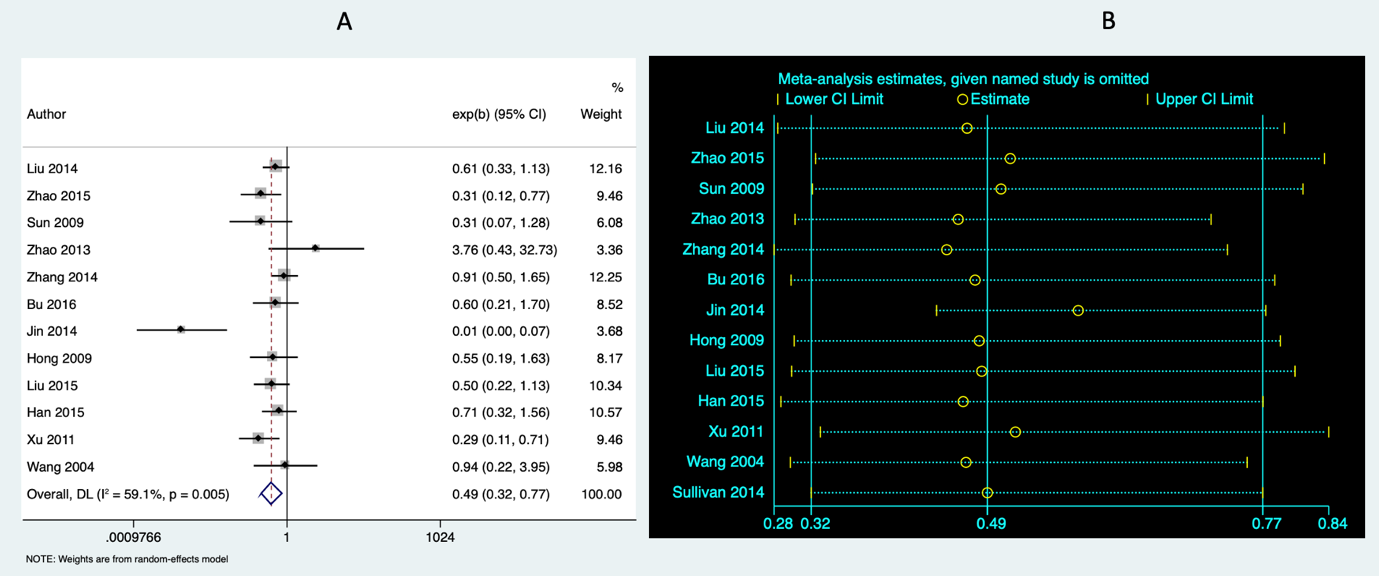


XRCC1 rs1799782 heterozygous model


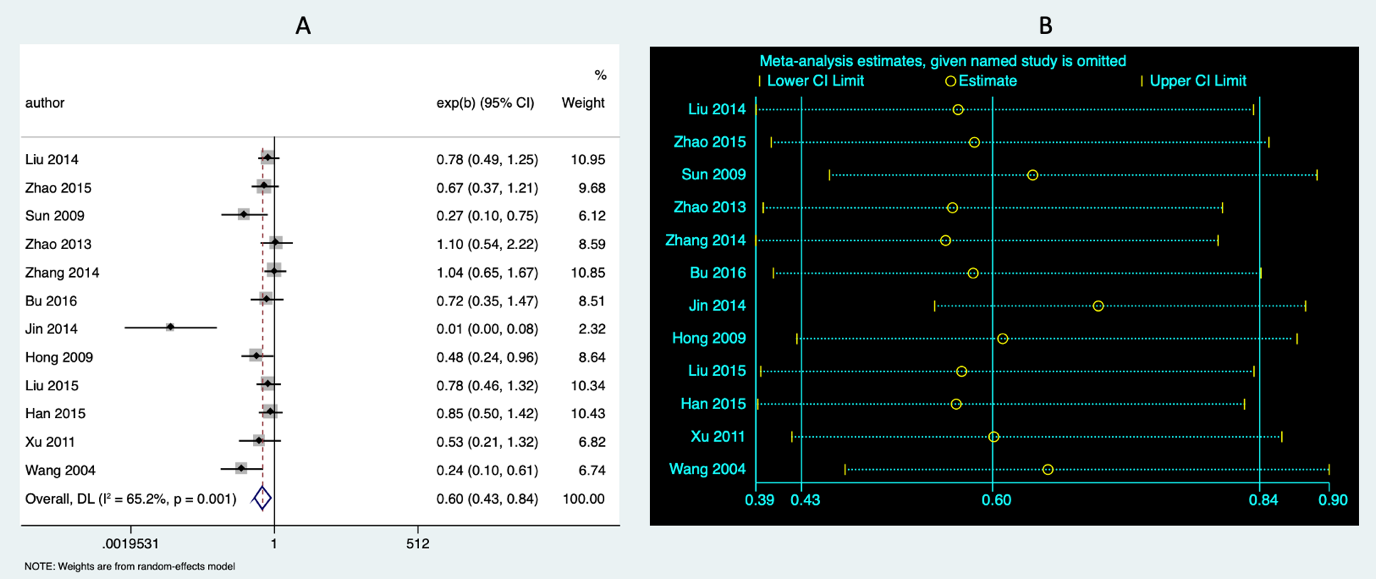


XRCC1 rs1799782 dominant model


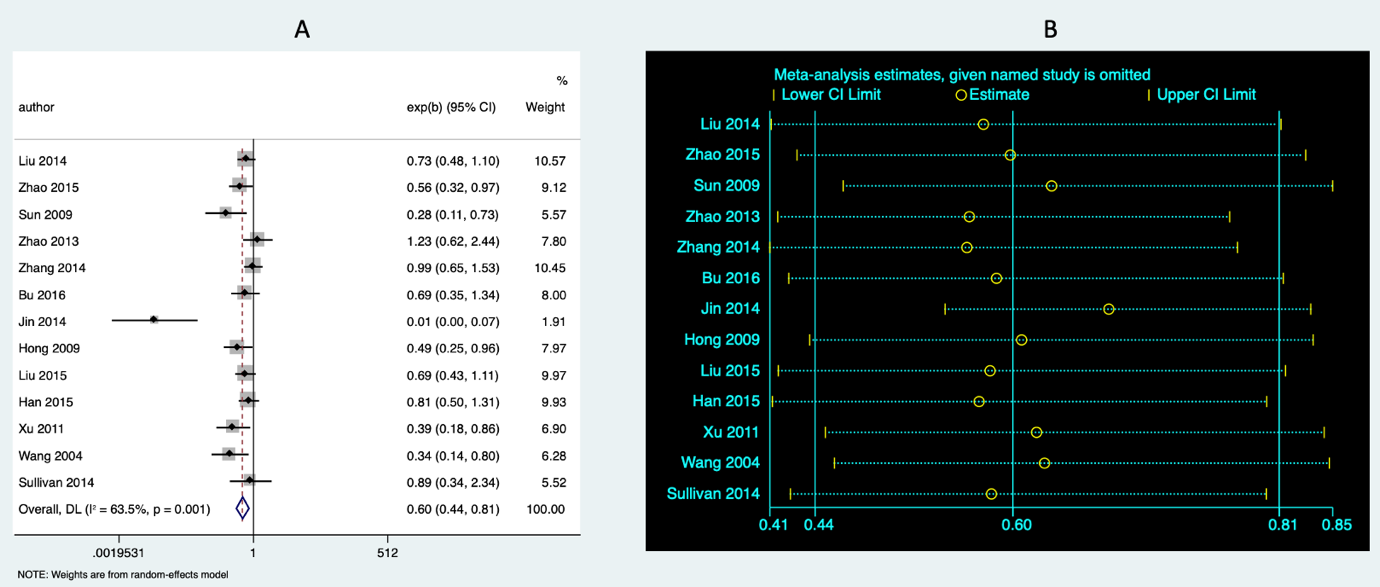


*XRCC1* rs1799782 recessive model


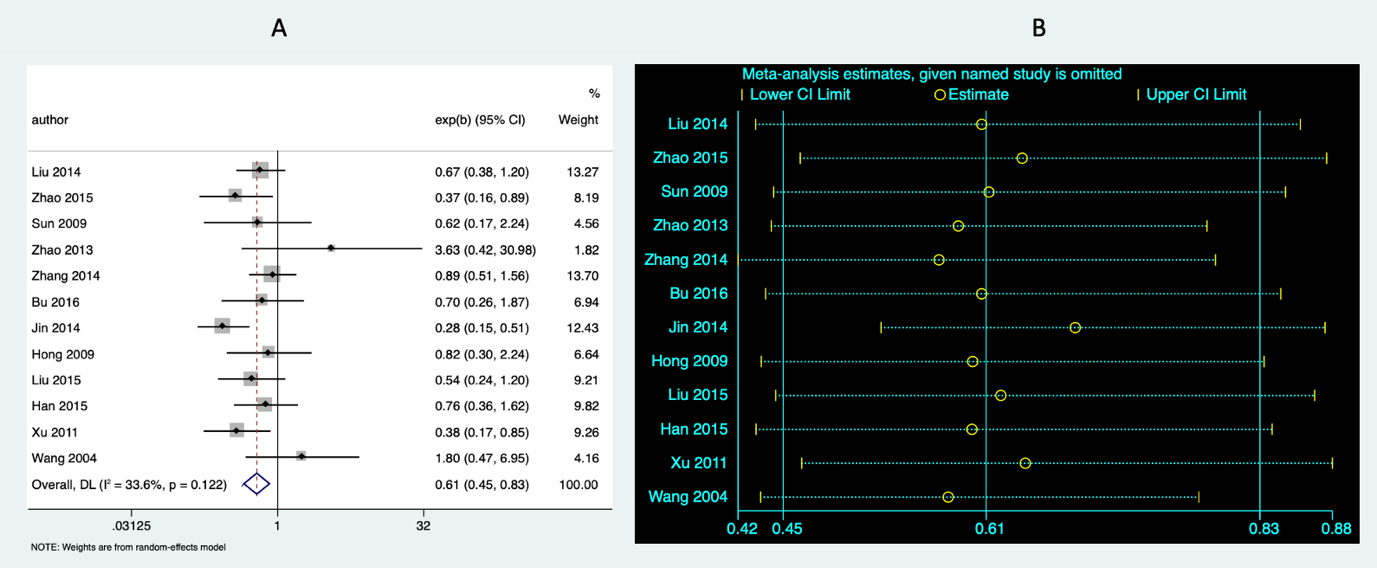


XRCC1 rs1799782 allele model


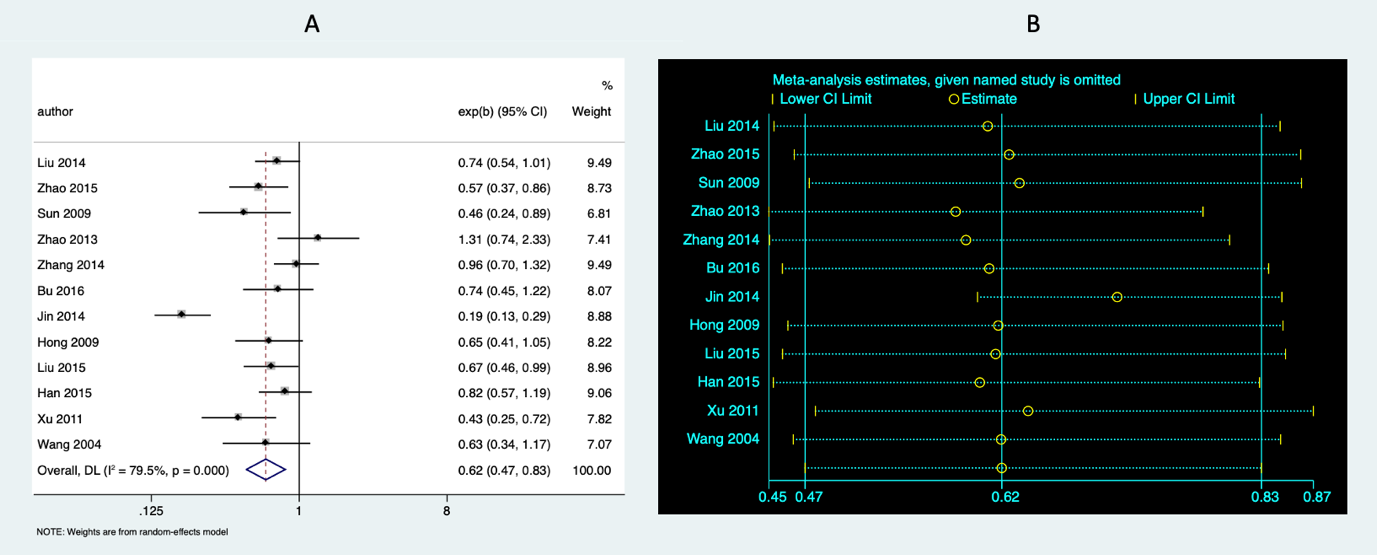


**Figure_SuppInfo_10** Forest plots (A) and the sensitivity analysis (B) of the association between *XRCC1* rs1799782 and response to PBC

XRCC1 rs25487 homozygous model


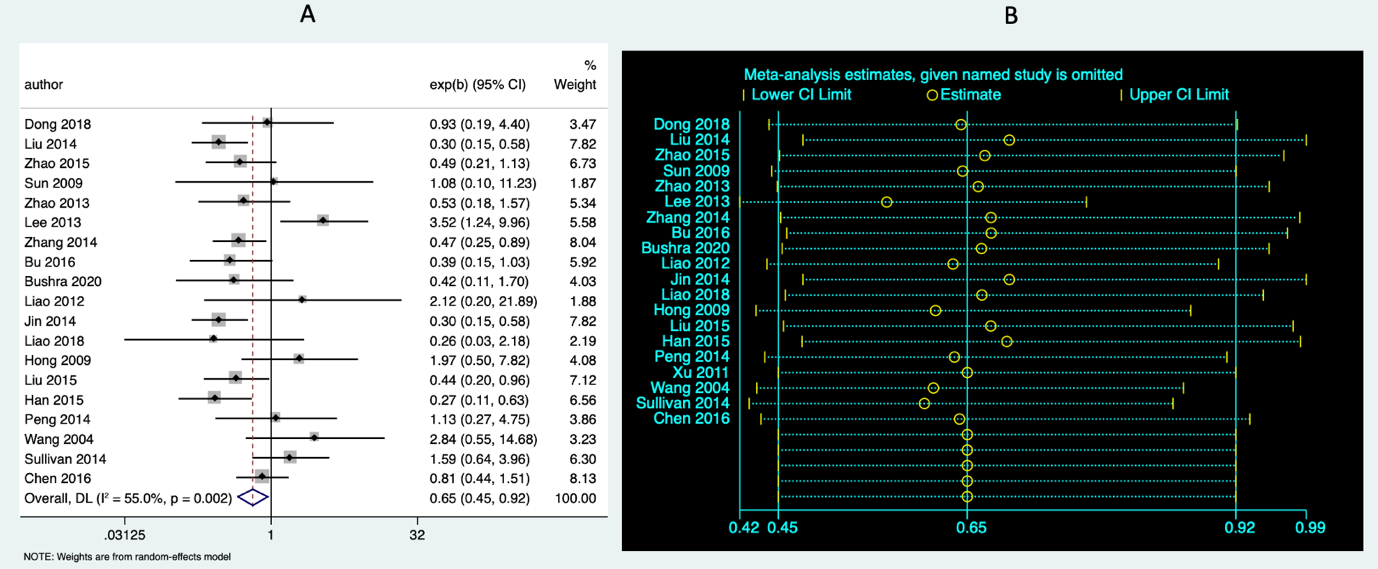


XRCC1 rs25487 recessive model


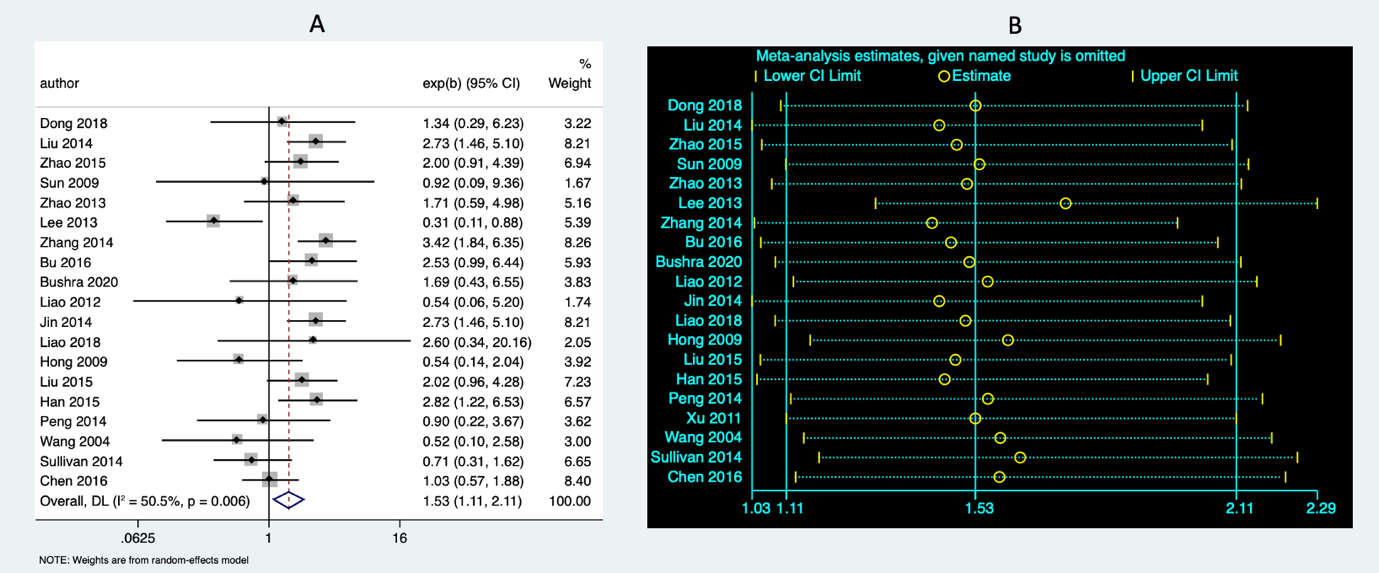


**Figure_SuppInfo_11** Forest plots (A) and the sensitivity analysis (B) of the association between *XRCC1* rs25487 and response to PBC

*XRCC3* rs861539 dominant model


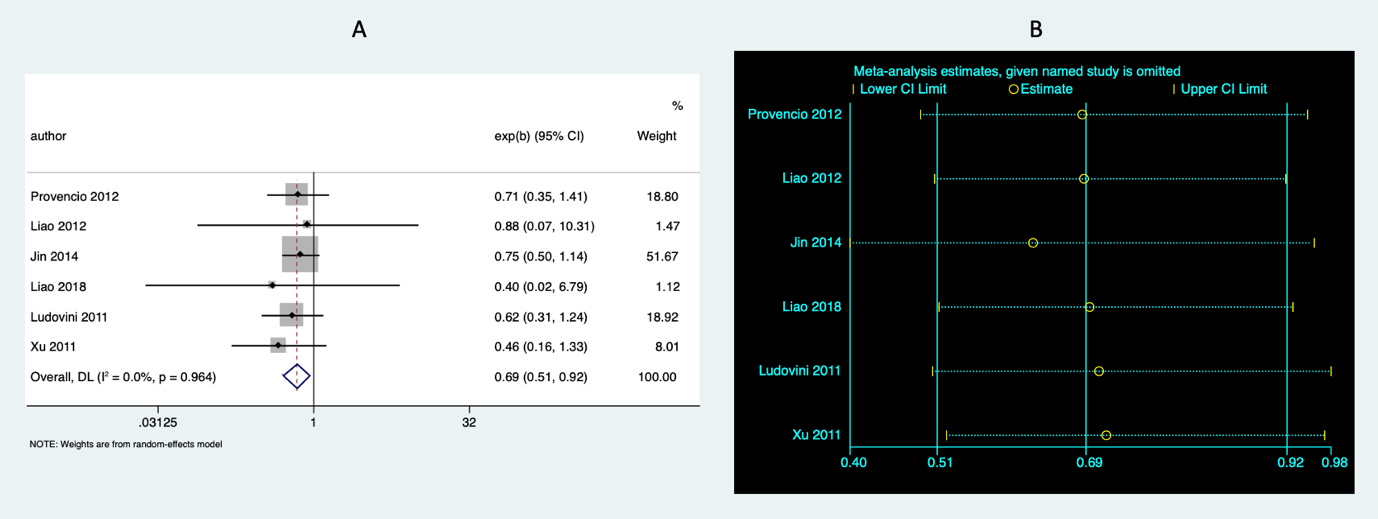


*XRCC3* rs861539 allele model


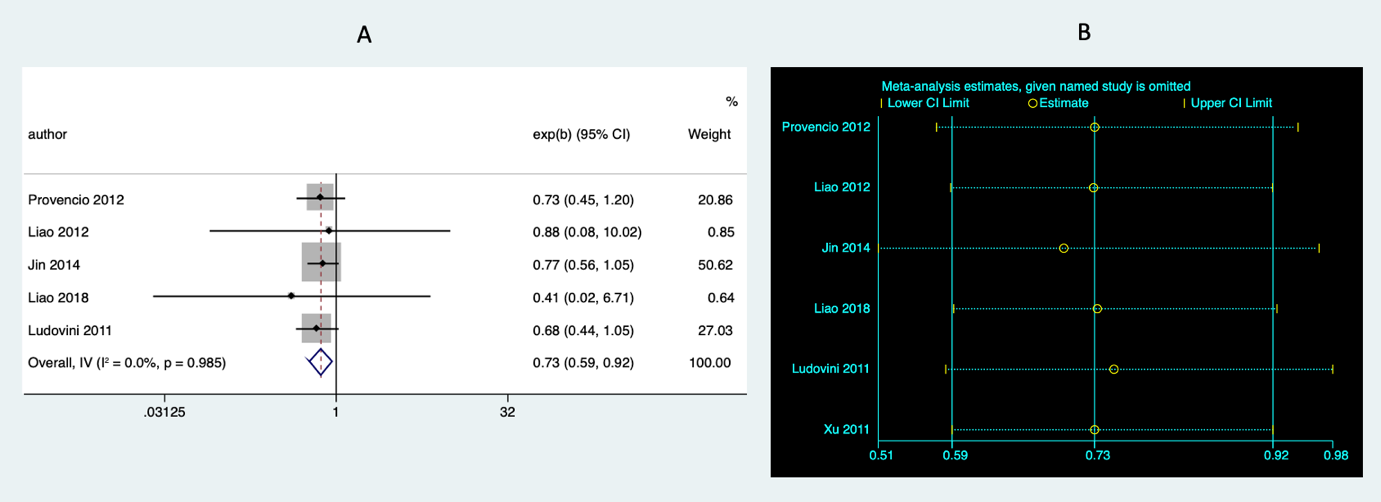


**Figure_SuppInfo_12** Forest plots (A) and the sensitivity analysis (B) of the association between *XRCC3* rs861539 and response to PBC

*ERCC1* rs11615 homozygous model


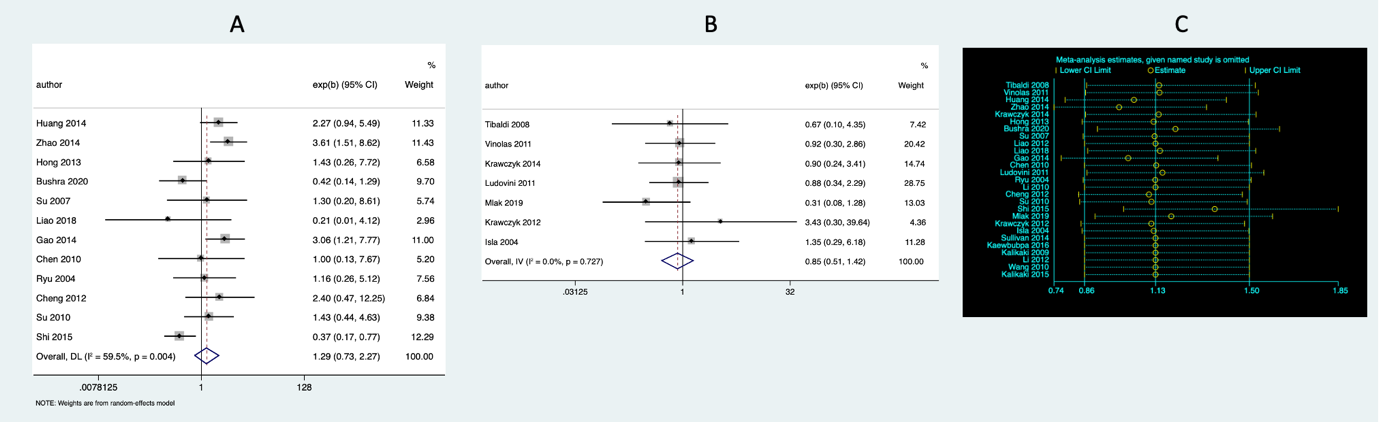


*ERCC1* rs11615 heterozygous model


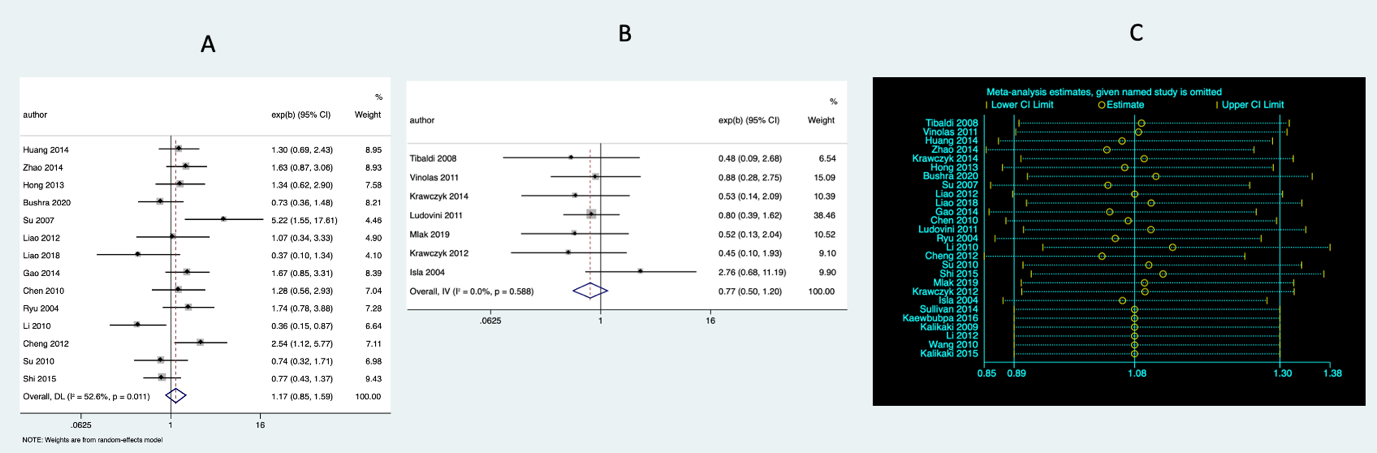


ERCC1 rs11615 dominant model
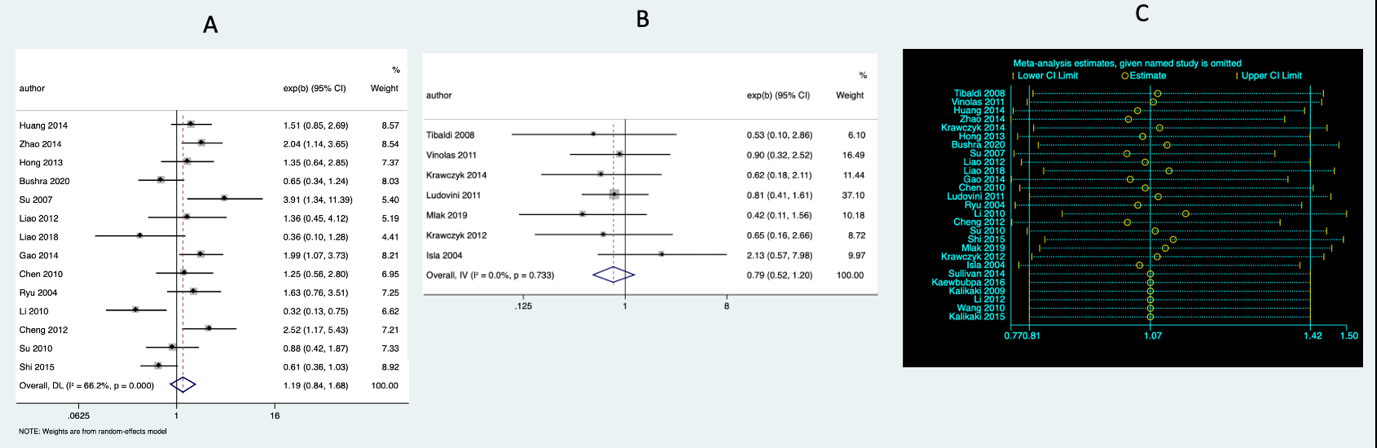


*ERCC1* rs11615 recessive model
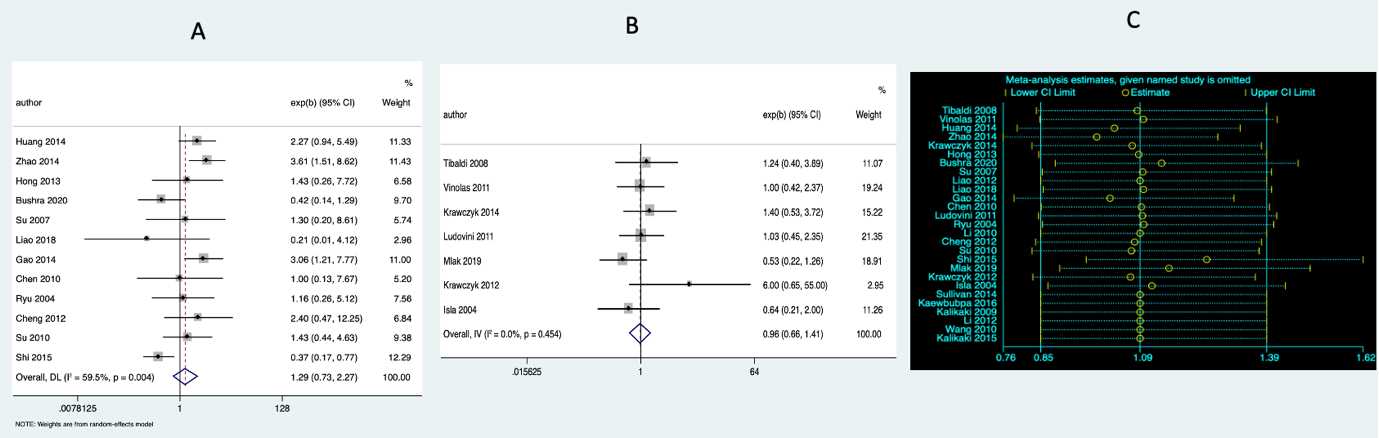


*ERCC1* rs11615 allele model


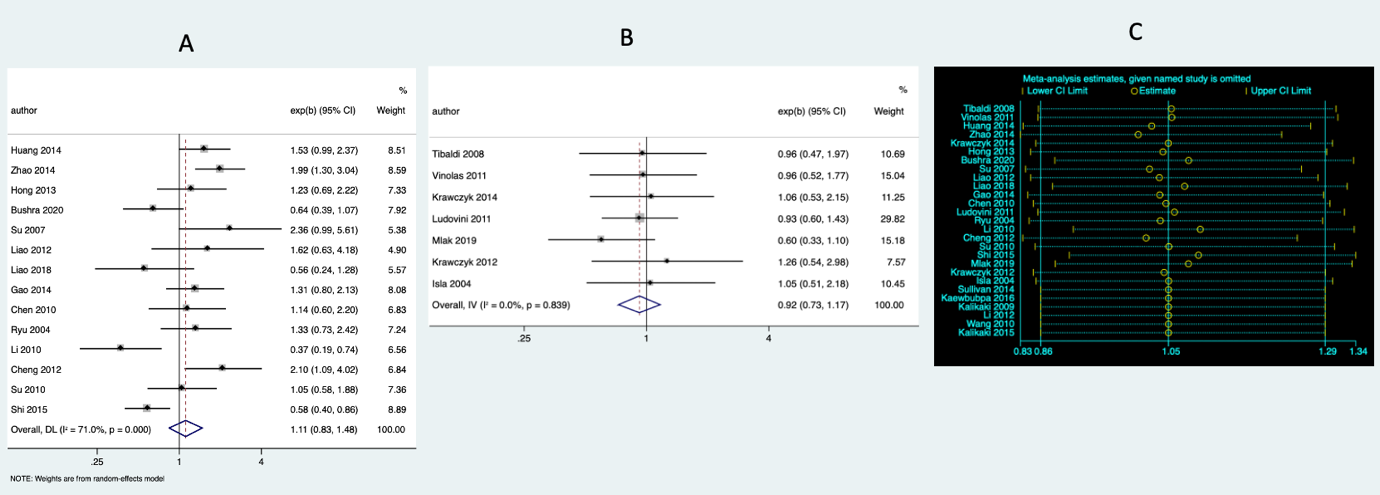


**Figure_SuppInfo_13** Forest plots of the association between ERCC1 rs11615 and response to PBC in Asian (A) and European (B) populations, and (C) the sensitivity analysis for the genetic associations

*ERCC1* rs3212986 homozygous model


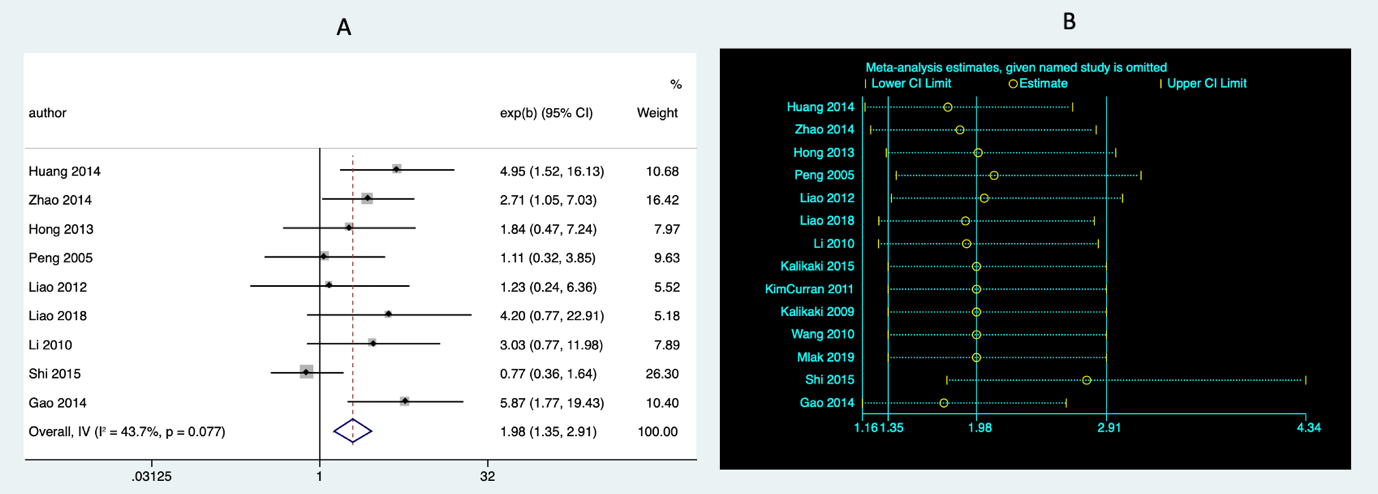


*ERCC1* rs3212986 recessive model


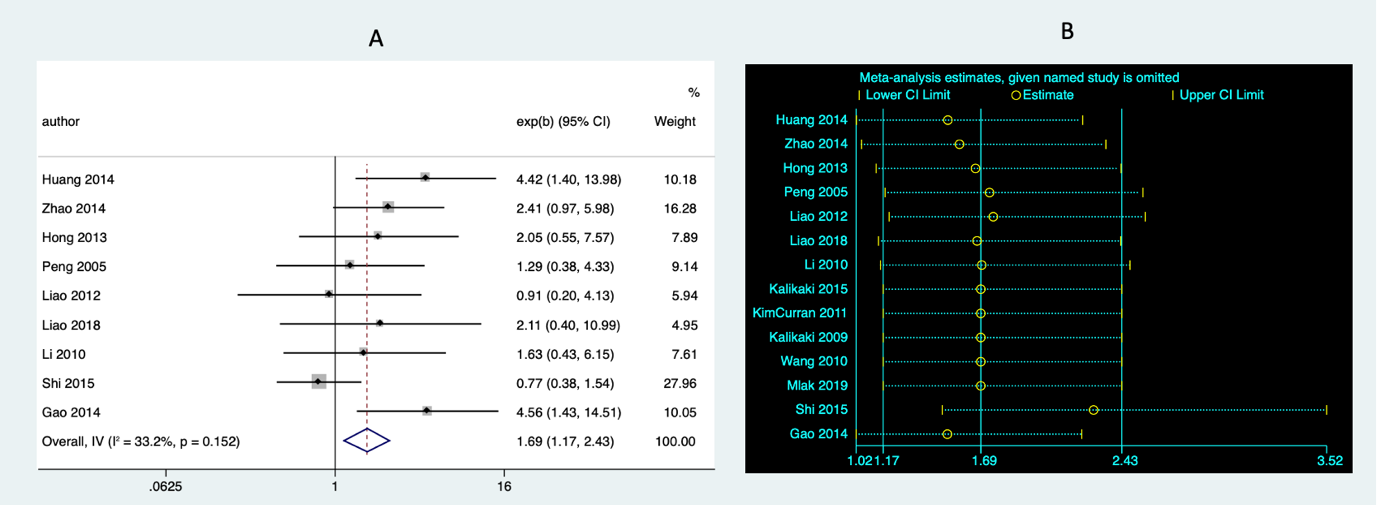


*ERCC1* rs3212986 dominant model (subgroup analysis)


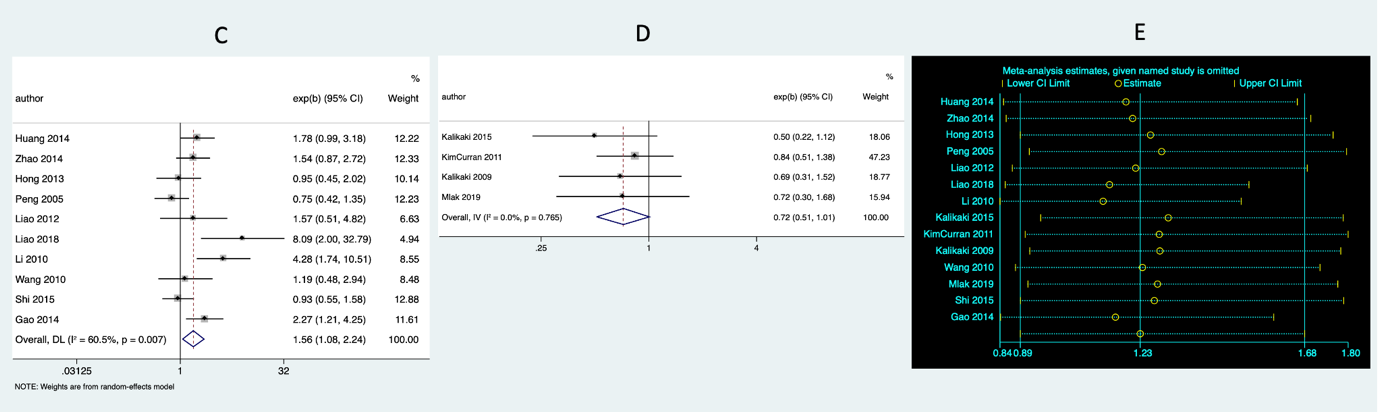


**Figure_SuppInfo_14** Forest plots (A) and the sensitivity analysis (B) of the association between ERCC1 rs3212986 and response to PBC. The subgroup analysis forest plots are classified into (C) Asian and (D) European populations, and (E) shows the sensitivity analysis in the dominant model

ERCC2 rs13181 subgroup analysis heterozygous model


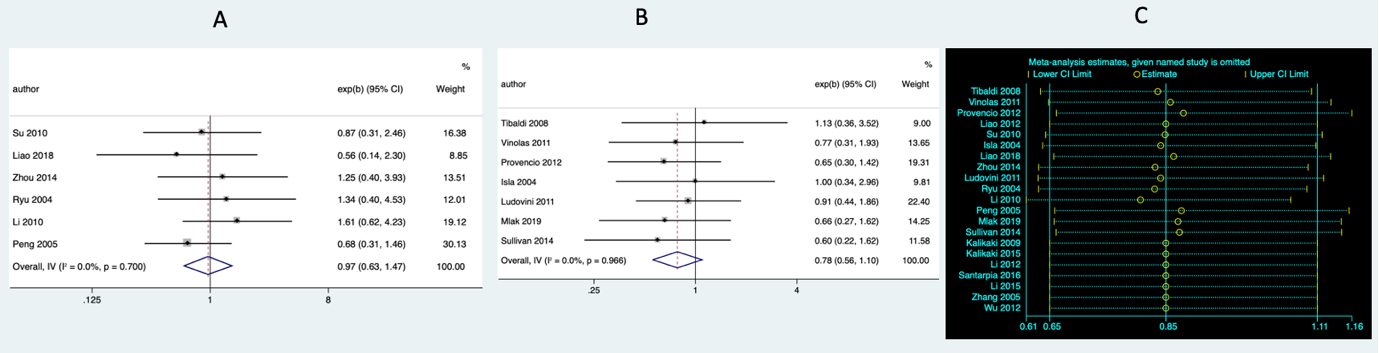


ERCC2 rs13181 subgroup analysis dominant model


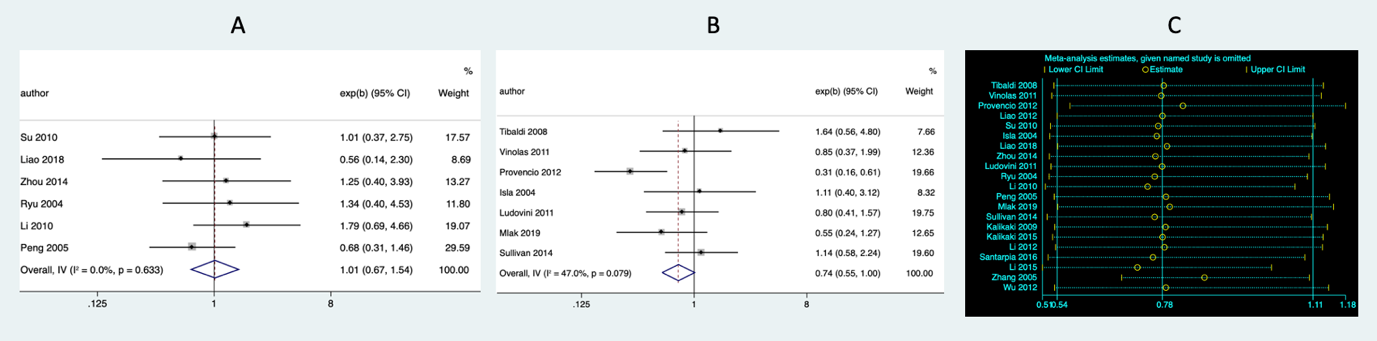


ERCC2 rs13181 subgroup analysis allele model


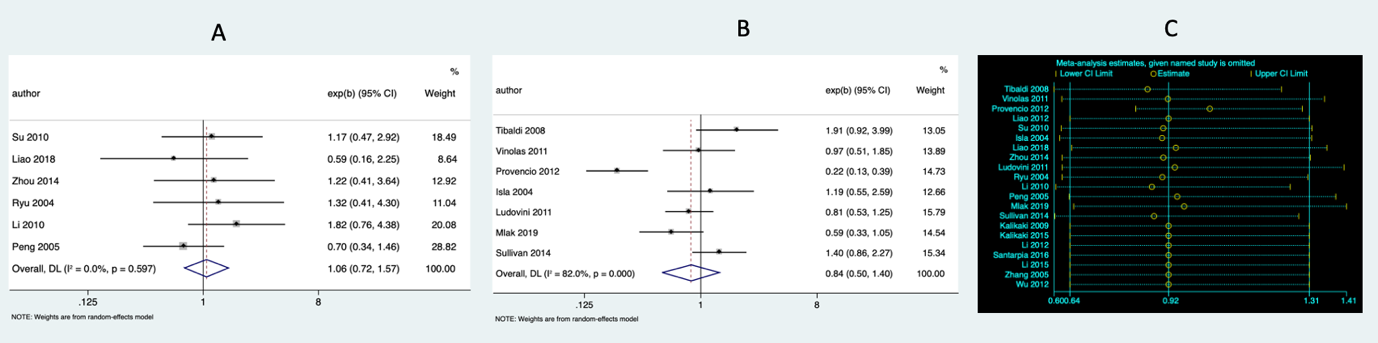


**Figure_SuppInfo_15** Forest plots of the association between ERCC2 rs13181 and response to PBC in Asian (A) and European (B) populations, and the sensitivity analysis for the genetic associations (C)

*ABCC2* rs717620 allele model

**
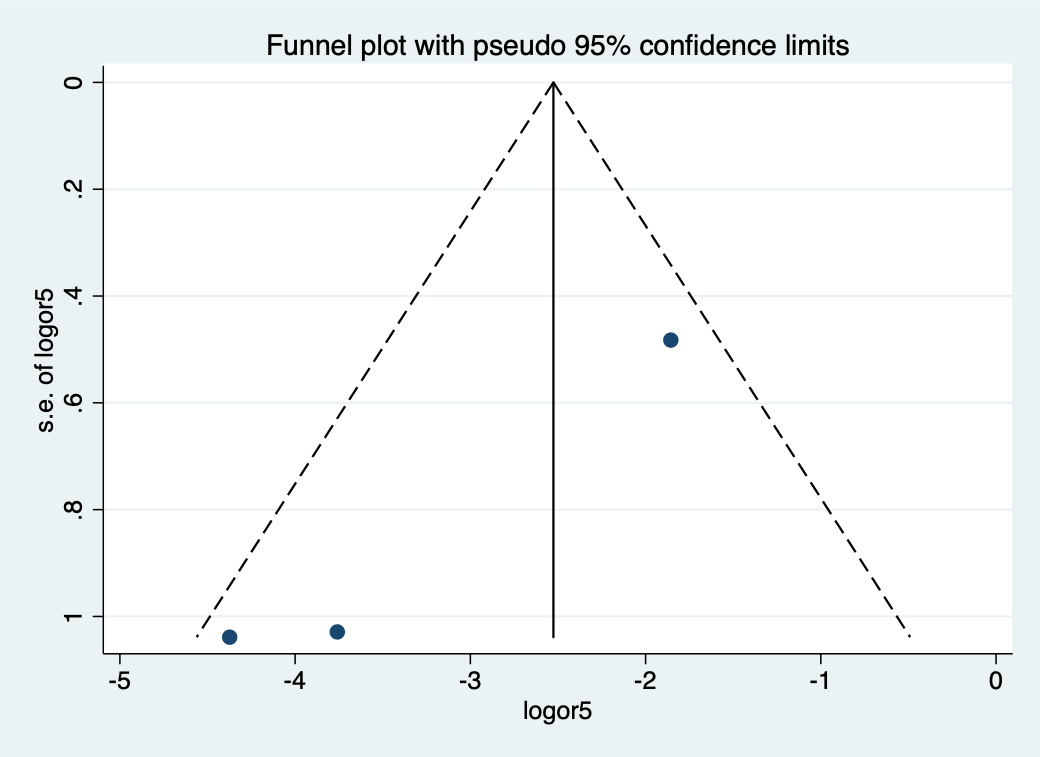
**

**Figure_SuppInfo_16** Funnel plot of the association between *ABCC2* rs717620 and response to PBC

*ABCG2* rs2231142 homozygous model


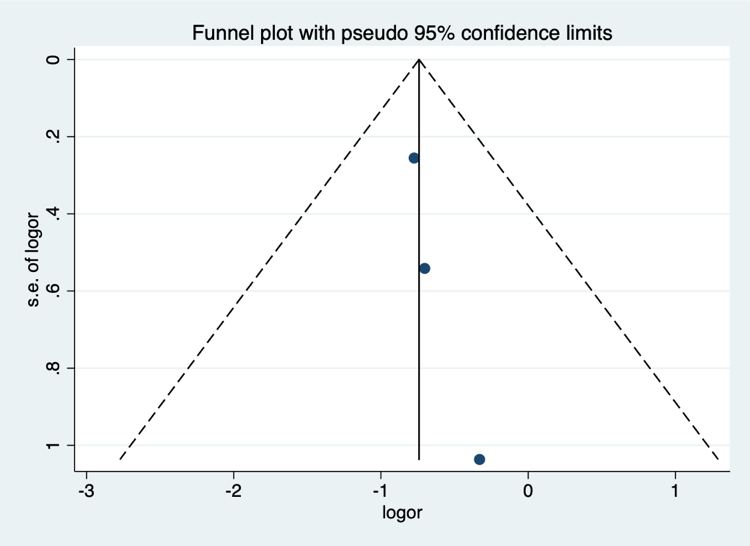


*ABCG2* rs2231142 recessive model


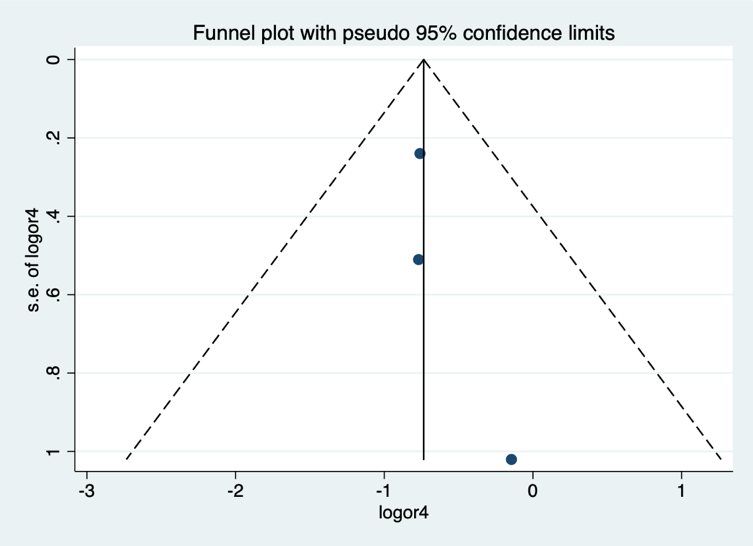


*ABCG2* rs2231142 allele model


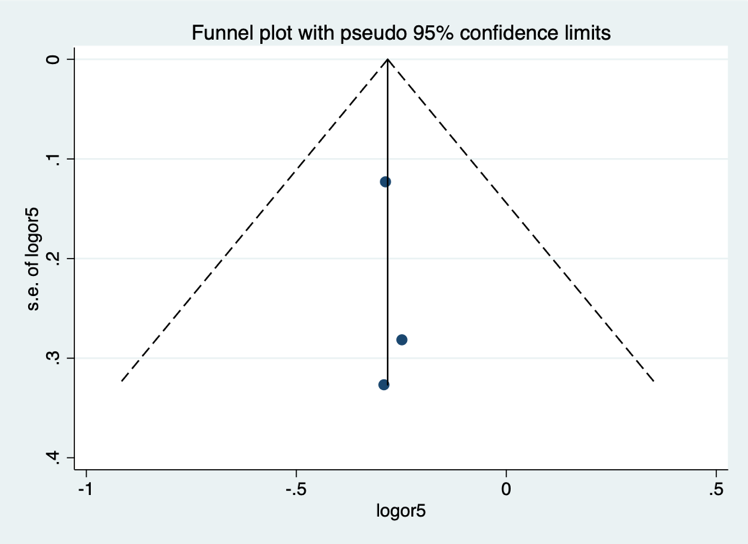


**Figure_SuppInfo_17** Funnel plots of the association between *ABCG2* rs2231142 and response to PBC

*CDA* rs1048977 allele model


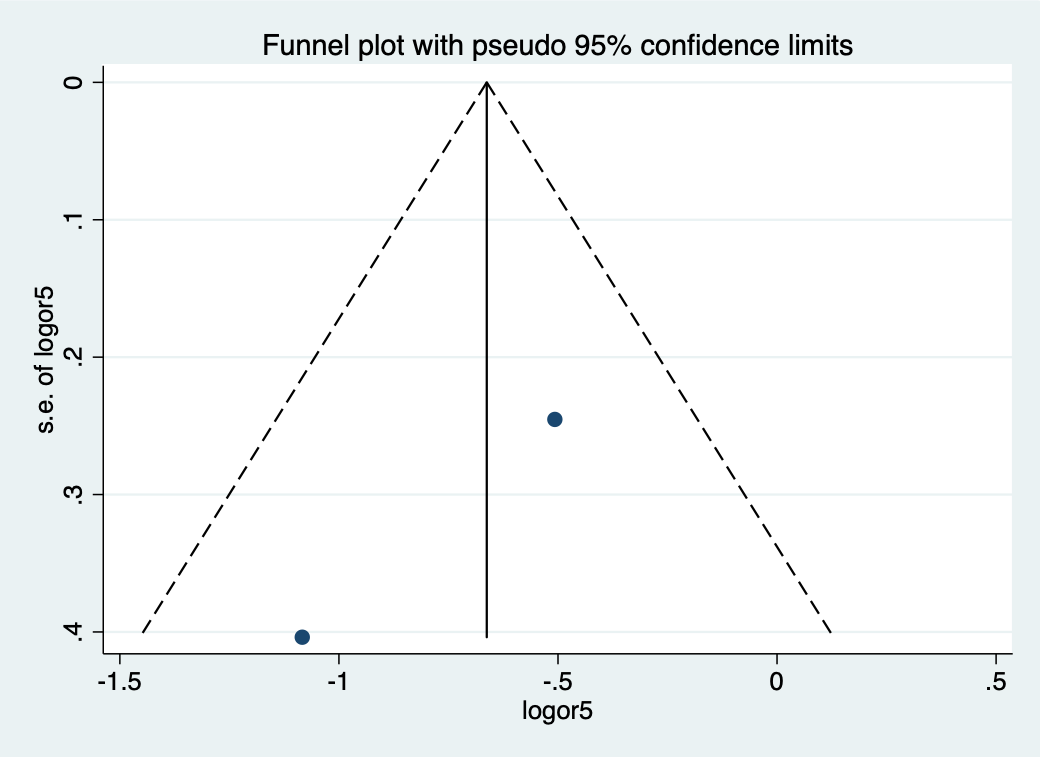


**Figure_SuppInfo_18** Funnel plot of the association between *CDA* rs1048977 and response to PBC

*CYP1A1* rs1048943 heterozygous model


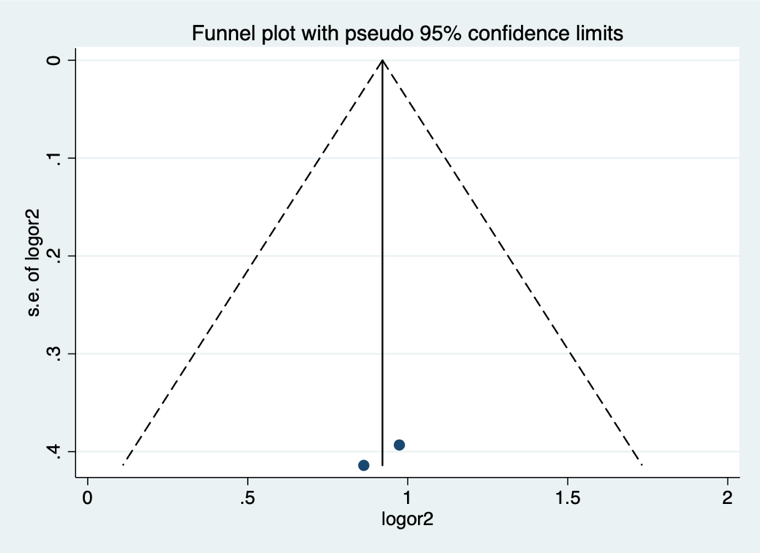


*CYP1A1* rs1048943 dominant model


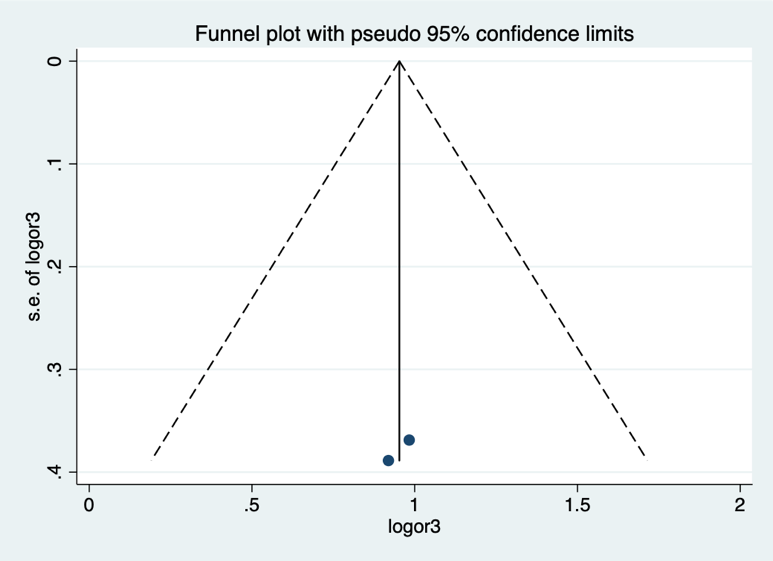


*CYP1A1* rs1048943 allele model


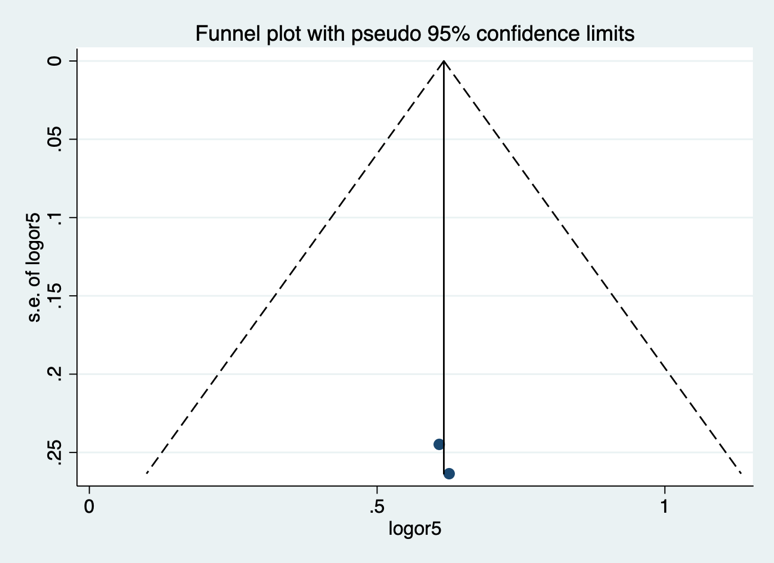


**Figure_SuppInfo_19** Funnel plots of the association between *CYP1A1* rs1048943 and response to PBC

*ERCC1* rs3212986 recessive model


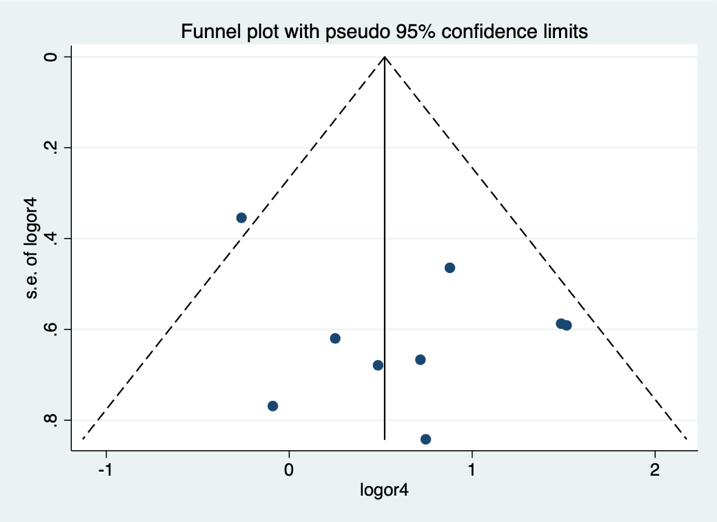


*ERCC1* rs3212986 allele model


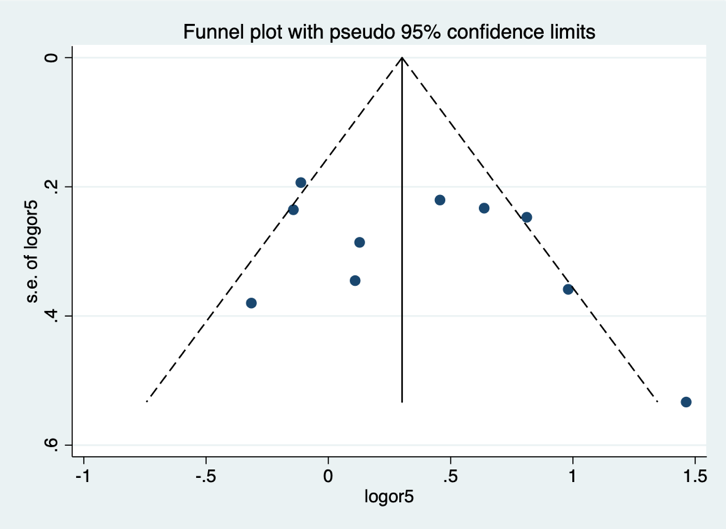


**Figure_SuppInfo_20** Funnel plots of the association between *ERCC1* rs3212986 and response to PBC

*ERCC2* rs1799793 dominant model


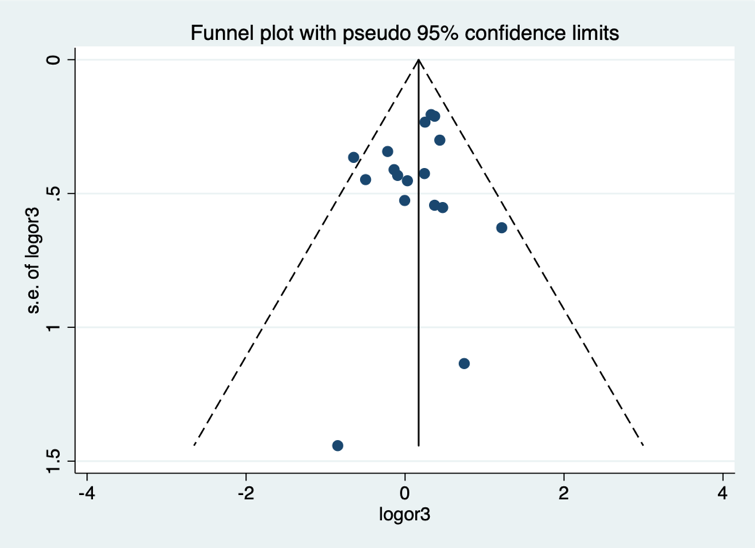


*ERCC2* rs1799793 allele model


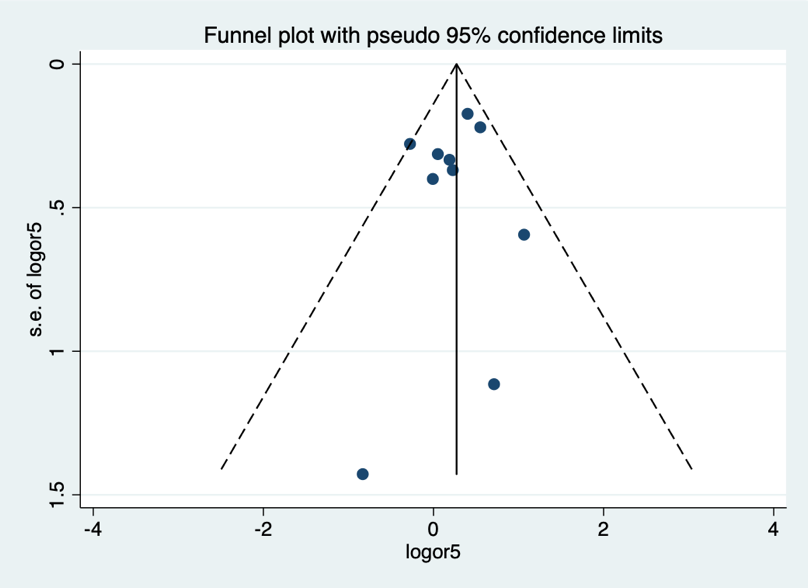


**Figure_SuppInfo_21** Funnel plots of the association between *ERCC2* rs1799793 and response to PBC

*ERCC2*rs1052555 dominant model


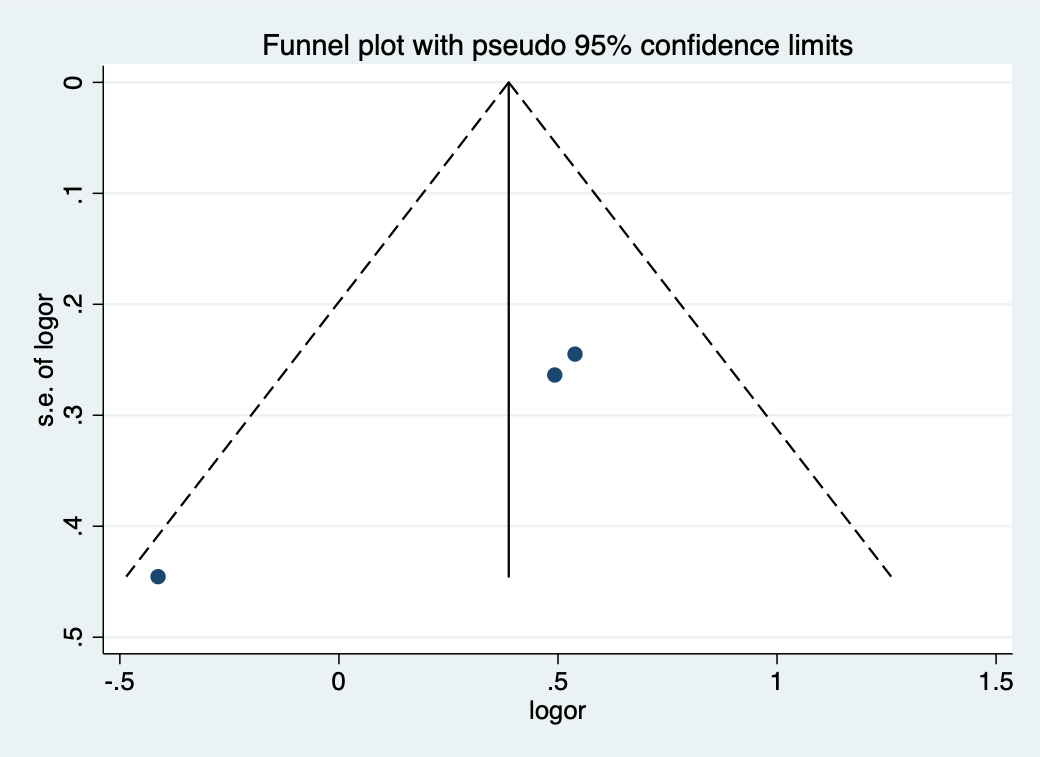


**Figure_SuppInfo_22** Funnel plot of the association between *ERCC2*rs1052555 and response to PBC

*GSTM1* rs36631 allele model


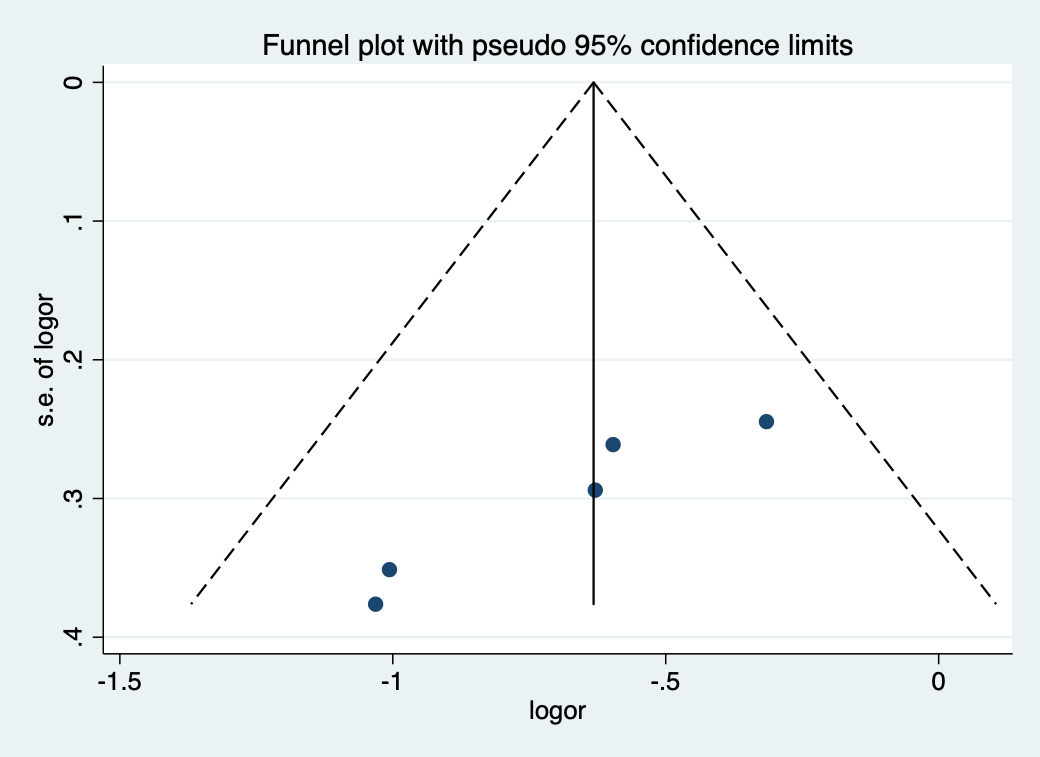


**Figure_SuppInfo_23** Funnel plot of the association between *GSTM1* rs36631 and response to PBC

*XPC* rs77907221 heterozygous model


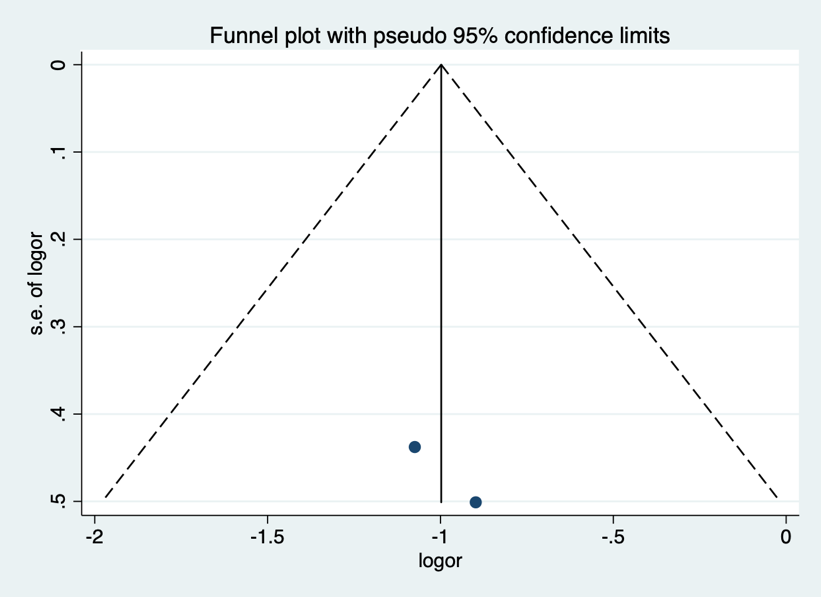


*XPC* rs77907221 allele model


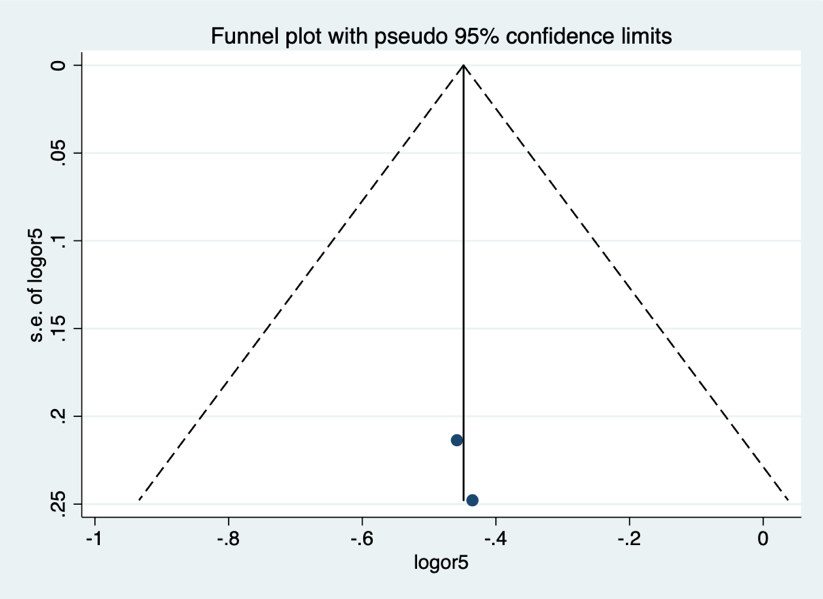


**Figure_SuppInfo_24** Funnel plot of the association between *XPC* rs77907221 and response to PBC

*XRCC1* rs1799782 homozygous model


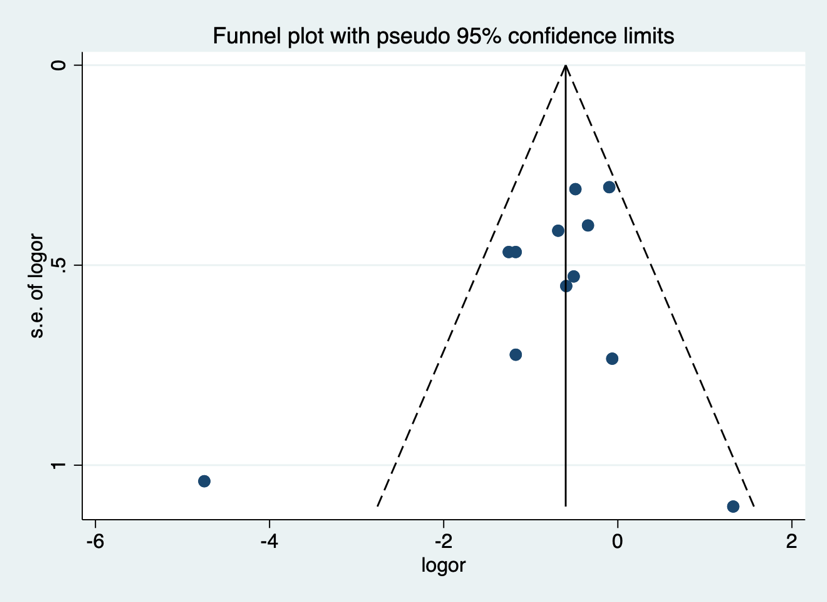


*XRCC1* rs1799782 recessive model


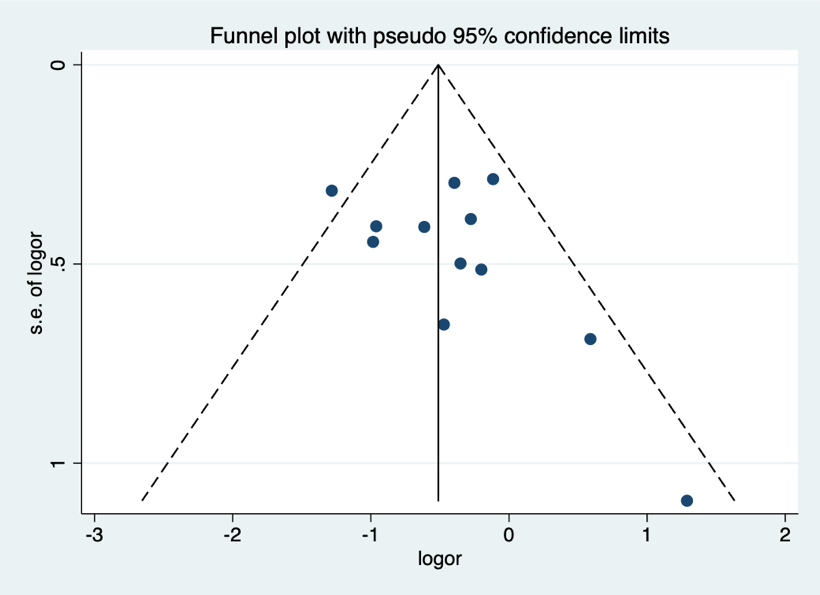


*XRCC1* rs1799782 allele model


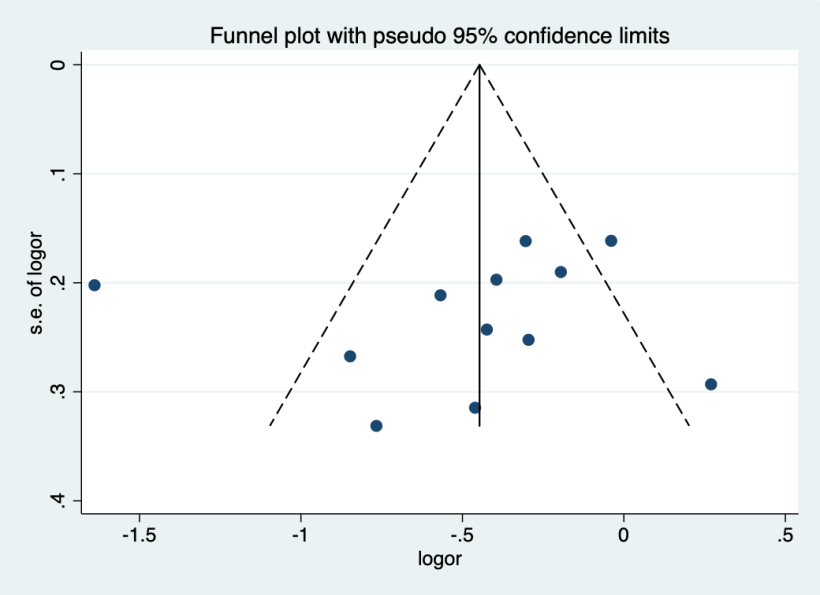


**Figure_SuppInfo_25** Funnel plot of the association between *XRCC1* rs1799782 and response to PBC

*XRCC1* rs25487 homozygous model


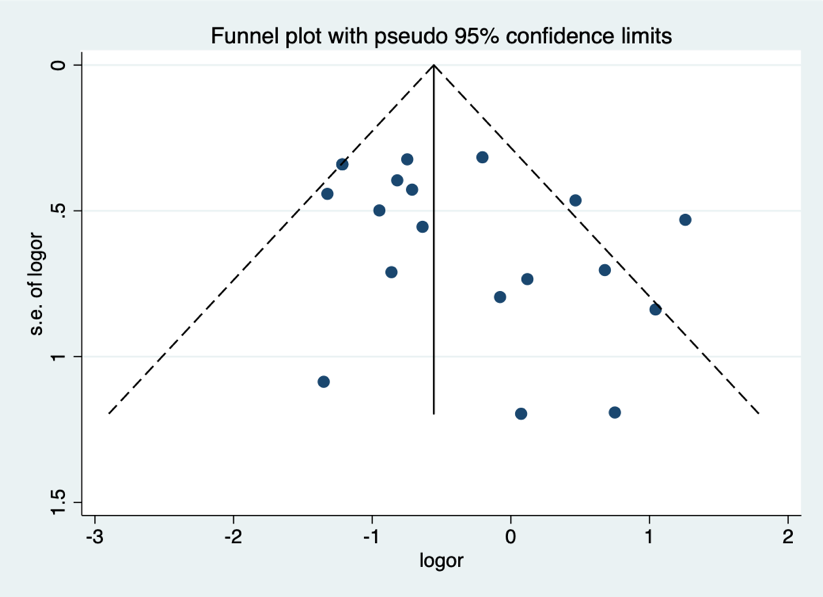


*XRCC1* rs25487 recessive model


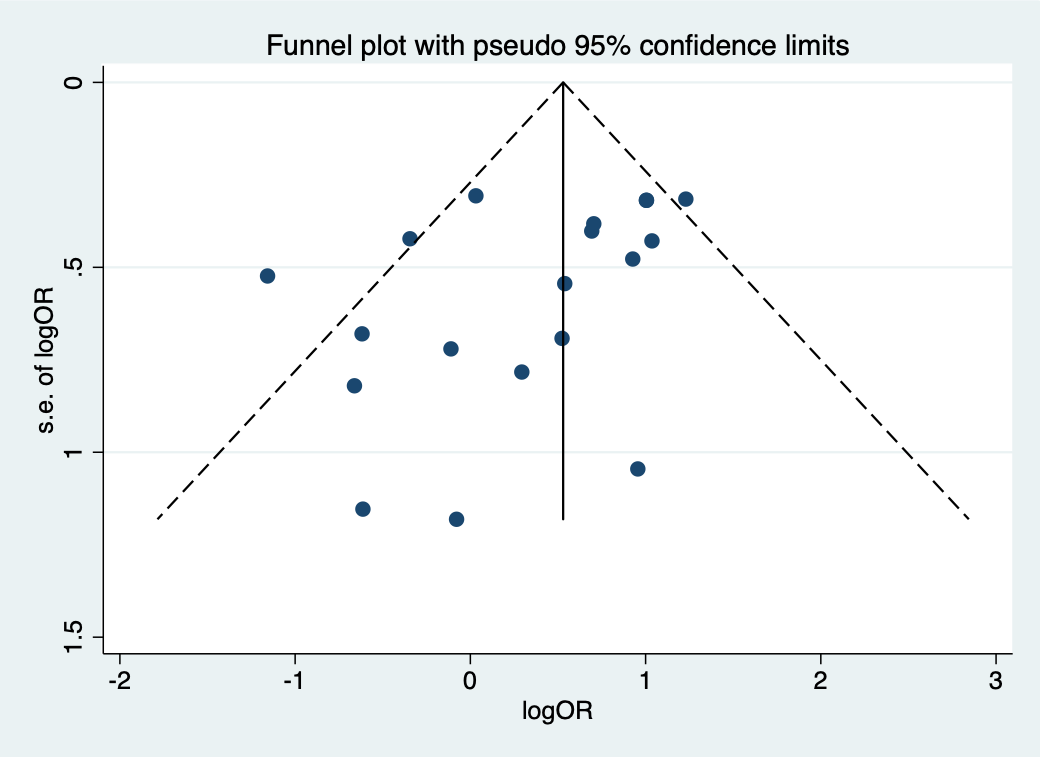


**Figure_SuppInfo_26** Funnel plot of the association between *XRCC1* rs25487 and response to PBC

*XRCC3* rs861539 dominant model


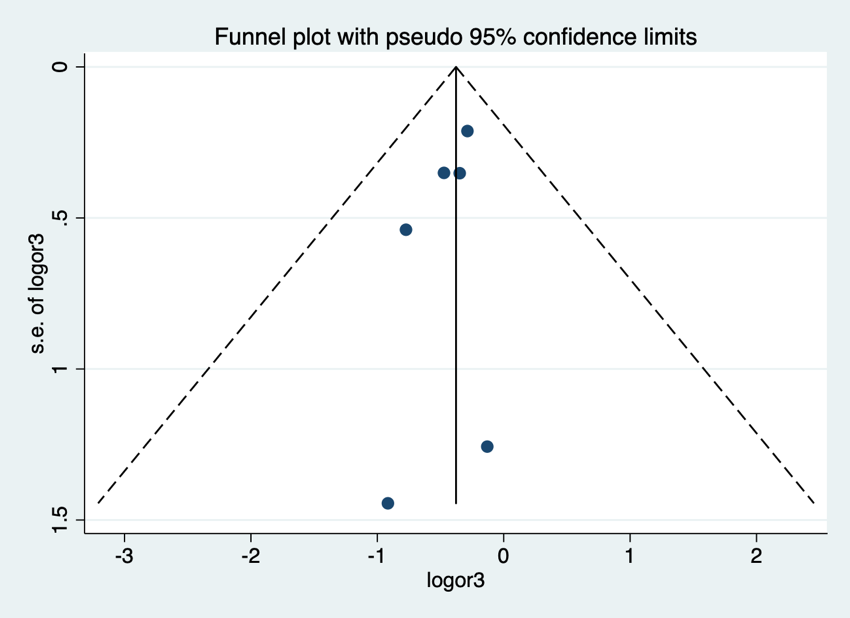


*XRCC3* rs861539 allele model


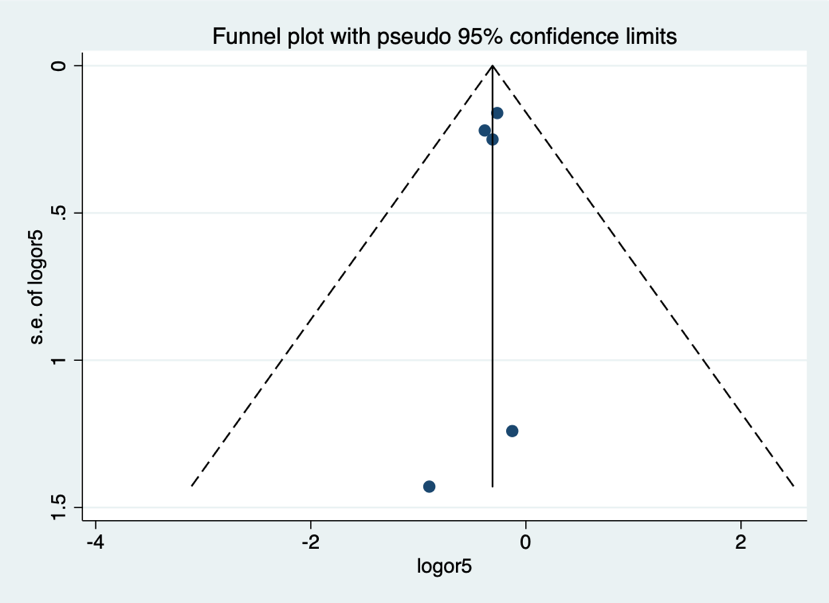


**Figure_SuppInfo_27** Funnel plot of the association between *XRCC3* rs861539 and response to PBC
